# Barley HvBODYGUARD1 controls cuticular specialisations regulated by SHINE transcription factors

**DOI:** 10.1111/nph.71287

**Published:** 2026-05-27

**Authors:** Trisha McAllister, Chiara Campoli, Linsan Liu, Tansy Chia, S. Ronan Fisher, Richard Horsnell, Alan R. Prescott, Jennifer Shoesmith, Mhmoud Eskan, Alasdair Iredale, Mirjam Nuter, Luke Ramsay, Micha M. Bayer, Linda Milne, Miriam Schreiber, Yogeswari Rajarathinam, Vanessa Wahl, Robbie Waugh, James Cockram, Sarah M. McKim

**Affiliations:** ^1^ Division of Plant Sciences, Faculty of Life Sciences University of Dundee at the James Hutton Institute Invergowrie DD2 5DA UK; ^2^ The James Hutton Institute Invergowrie DD2 5DA UK; ^3^ Niab Park Farm, Histon Cambridge CB24 9NZ UK; ^4^ DIF and Cell Signalling and Immunology, Faculty of Life Sciences University of Dundee Dundee DD1 5EH UK

**Keywords:** barley, BODYGUARD, cereal, cuticle, grain, SHINE transcription factors, wax bloom, wheat

## Abstract

- Land plants secrete a protective outer cuticular layer with diverse functions. Barley (*Hordeum vulgare* L.) develops two cuticular specialisations: the β‐diketone rich wax bloom on vegetative tissues and an adherent grain surface which sticks to the hulls, leading to barley's distinctive ‘covered’ grain phenotype. Two barley SHINE transcription factors, HvWIN1 and NUD, promote the wax bloom and covered grain phenotypes, respectively, yet we know little about other genes involved.
- We investigated a barley mutant showing defects in both cuticular specialisations to better understand the networks and processes underlying these traits.
- We identified the barley *BODYGUARD1* (*HvBDG1*) gene encoding an α/β hydrolase as crucial for leaf cuticular integrity and wax‐bloom deposition. Mining tetraploid wheat (*Triticum turgidum* ssp. *durum*) mutant populations demonstrated that BDG1 and WIN1 orthologues also control the wax bloom in wheat. We further reveal that NUD and HvWIN1 retain functional, independent overlap in leaf cuticle integrity and hull adhesion in barley, functions which involve upregulation of *HvBDG1* and other direct or indirect targets, shared and distinct, depending on the stage and tissue.
- Our work greatly expands our knowledge of genetic and developmental mechanisms underlying shared and distinctive cuticular features in plants.

## Graphical abstract

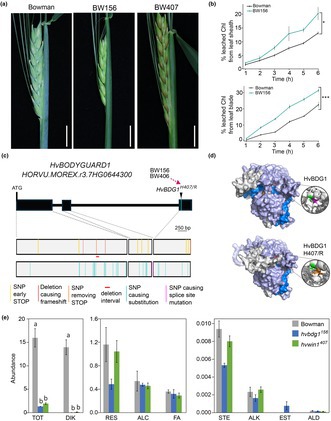

## Introduction

The plant cuticle is a hydrophobic barrier covering most aerial surfaces in land plants, protecting them from desiccation, pathogen attack and tissue fusion (González Valenzuela *et al*., 2023). Cuticles consist of a cutin matrix embedded with polysaccharides and cuticular waxes and overlaid with epicuticular waxes typically made from very long chain fatty acids (VLCFAs) and their derivatives (Yeats & Rose, 2013). However, within this framework, cuticles show remarkable differences. For instance, rather than the alcohol‐rich wax found in wild Triticeae cereal species (Tulloch *et al*., 1980), the leaf sheaths, stems and spikes of barley (*Hordeum vulgare* L.) and wheat (*Triticum* spp.) cereal crops develop glaucous (white‐blue) epicuticular wax blooms dominated by β‐diketones (Mikkelsen, 1979; Adamski *et al*., 2013). Wax blooms are drought responsive and confer improved water use efficiency and ultraviolet light reflectance, possibly explaining their selection in cultivated cereals (Richards *et al*., 1986; Bi *et al*., 2017).

Studying cuticle mutants, particularly the *eceriferum* or *cer* (‘not bearing wax’), fusion or glossy mutants helped identify genes encoding metabolic components, transporters, signalling proteins and transcription factors involved in cuticle formation and maintenance (Samuels *et al*., 2008). Many components appear broadly conserved across species (Kong *et al*., 2020; Liu *et al*., 2025); for instance, the WAX‐INDUCER/SHINE (SHINE)‐like transcription factors (WIN/SHN) are critical for cuticle development in Arabidopsis, rice, wheat and tomato (Kannangara *et al*., 2007; Shi *et al*., 2011, 2013; Wang *et al*., 2012; Zhou *et al*., 2014; Bi *et al*., 2018), while the GDS(L) [Gly, Asp, Ser, (Leu)] motif esterase/lipases (Brick *et al*., 1995) in tomato, barley and rice promote cutin accumulation and water retention (Park *et al*., 2010; Girard *et al*., 2012; Li *et al*., 2017). Other components appear more specialised, such as the *CER‐CQU* metabolic gene cluster encoding a polyketide synthase producing β‐diketones in barley and wheat (Hen‐Avivi *et al*., 2016; Schneider *et al*., 2016). We recently demonstrated in barley that defective alleles in *HvGDSL1* or *HvWIN1* cause reduced wax blooms, likely from lower *CER‐CQU* expression (McAllister *et al*., 2022; Campoli *et al*., 2024). Barley also has a unique cuticular specialisation on its caryopsis (Supporting Information Fig. S1) where the outer pericarp cuticle sticks to the inner face of the floret hulls, leading to barley's ‘covered grain’ phenotype (Gaines *et al*., 1985). Early in postdomestication cultivation, deletion of *NUDUM* (*NUD*), another *WIN/SHN* homologue, caused loss of hull adhesion resulting in naked grain barley subsequently cultivated for human food (Taketa *et al*., 2008). We know little about the steps downstream of NUD which promote adhesion.

While naked barley for human food is a growing market, most barley is grown for animal feed or malting and brewing where a strongly adherent hull protects the grain and increases malting efficiency (Okoro *et al*., 2017). Grain ‘skinning’ (partial hull shed) of covered barley is a worsening problem that causes low quality malt (Okoro *et al*., 2017). We observed that a subset of barley *cer* mutants also show grain skinning (Campoli *et al*., 2024) and speculated that these mutants could reflect disrupted targets downstream of NUD. Here, we reveal that one such *cer* mutant results from defective alleles in the barley *BODYGUARD1* (*HvBDG1*) gene, named due to homology with the BDG α/β hydrolase necessary for cuticular integrity in Arabidopsis (Kurdyukov *et al*., 2006a; Jakobson *et al*., 2016). Our evidence suggests that HvWIN1, HvBDG1 and NUD have unique and overlapping roles in developing barley grain and leaves, and that BDG1 and WIN1‐mediated promotion of wax blooms is conserved in the related crop species durum wheat (*T. turgidum* ssp. *durum* (Desf.) Hunsn.). Collectively, this work advances understanding of the networks and genetic mechanisms controlling cuticular integrity and specialisations in plants.

## Materials and Methods

### Germplasm and growth conditions

Barley (*Hordeum vulgare* L.) germplasm details are provided in Table S1. Barley cultivar (cv) Bowman and the Bowman near‐isogenic lines (BW‐NILs) for introgressed *cer* alleles (BW156, BW406, BW407 and BW126; Druka *et al*., 2011) were obtained from the James Hutton Institute (JHI), UK. Original barley mutant alleles and parent cvs were obtained from the Nordic Genetic Resource Center (NordGen), the US National Plant Germplasm Service (NPGS) and Okayama University, Japan. Barley plants were grown in glasshouses under long day photoperiod conditions (16 h : 8 h, 18°C : 14°C, light : dark) in standard cereal compost mix: 1.2 m^3^ Peat, 100 l sand, 1.5 kg osmocote exact start, 3.5 kg osmocote exact, 2.5 kg lime, Ca and Mg, 0.5 kg Celcote, 100 l perlite and 280 g intercept. Double mutants were generated by crossing and confirmed by genotyping F_2_ populations. Plants used in grain skinning and debranner assays were grown in pots in an outdoor polytunnel at JHI Dundee, with automated watering (3 min thrice daily) and disease control (Opus Team and Hallmark sprays). Polytunnel potting mix was composed of the following: 70% peat/30% wood fibre, lime, Nutricote 70 d (16‐10‐10), Base (15‐10‐20+TE), Nitrochalk, Mircomax, Aquasorb 3005, a wetting agent and perlite.

### Grain skinning and debranner assays

Mature grain from plants grown during spring/summer of 2014 and 2018 was assessed using manual skinning and debranner assays, respectively. Grains from a single plant were manually threshed and examined for skinning (Bowman, *n* = 1 pooling three plants; BW156, BW406, *n* = 4; BW126, *n* = 3). The debranner assay used 25 g of grain from a single plant (Bowman, *n* = 3; BW156, BW406 and BW126, *n* = 4; BW407, *n* = 5) processed in a Lab Scale Debranner TM05 (Satake Seed Mill) for 15 s, as described in Campoli *et al*. (2024). Double mutants were assessed by manual threshing with Bowman and mutant parent controls (*n* = 5). Significance was tested using ANOVA and Tukey's HSD.

### Cuticle integrity

Sections of fresh tissue (10 cm) from the second fully expanded leaf blade at growth stage 12 (GS12; Zadoks *et al*., 1974; *n* = 4) and flag leaf sheaths at GS55 (Bowman, *n* = 3; BW156, *n* = 2; double mutant experiment, *n* = 4) were assessed for Chl leaching as in McAllister *et al*. (2022), except total Chl leached was measured after up to 72 h; measurements were plotted as a percentage of this total. Data evaluated by ANOVA and Tukey's HSD using area under the curve. Mid‐sections of second leaf blades from 2‐wk‐old wheat plants were stained with 0.05% Toluidine Blue for 5 h (*n* = 4).

### Electron microscopy

Barley caryopses were harvested at 7 and 11 d post‐anthesis (DPA; *n* = 5). Sections from the central dorsal surface were examined by scanning electron microscopy (SEM) and transmission electron microscopy (TEM) as described in Campoli *et al*. (2024).

### Lipid chemistry

Soluble surface lipids were extracted from five and 11 DPA caryopses and hulls, derivatised and analysed using gas chromatography‐mass spectrometry (GC‐MS) as in Campoli *et al*. (2024) with statistics called with Tukey's HSD multiple comparison following one‐way ANOVA (*n* = 3). After surface lipid extractions, samples were returned to DCM for exhaustive delipidation over 2–3 wk with frequent solvent changes and fully dried before cutin extraction and analysis as in Campoli *et al*. (2024). Soluble surface lipids were extracted from 10 cm sections of flag leaf sheaths at ear emergence (GS51‐GS55) as above except they were extracted in 15 ml of DCM and derivatised in 10 μl BSTFA, 10 μl pyridine and 50 μl chloroform (*n* = 4, except *bdg* ^*156*^ and *nud* ^*638*^ where *n* = 3). Further run details are described in Notes S1. For both wax and cutin analysis, compound abundance was calculated relative to internal standard abundance and normalised by sample size (caryopses number for caryopses and hulls, and fresh weight for leaf sheaths).

### Gene mapping, candidate gene sequencing and whole‐genome survey sequencing

DNA from Bowman, cv Bonus and BW156 was genotyped with the 50 K iSelect single‐nucleotide polymorphism (SNP) array (Bayer *et al*., 2017) to identify donor introgressions in BW156. High‐confidence gene models within the introgression were identified using cv Morex V1 (GCA_901482405.1; Colmsee *et al*., 2015; Mascher *et al*., 2017) and V2 (GCA_902498975.1; Monat *et al*., 2019) genome assemblies and gene predictions in BARLEX. Coding sequences (CDS) were confirmed by comparing against cv Golden Promise genome assembly gene models (GCA_902500625.1; Schreiber *et al*., 2020) and homologous gene models in NCBI (Genes, 2004) and TAIR (Berardini *et al*., 2015). CDSs were amplified from BW156, Bowman and Bonus DNA (primers and reaction conditions; Table S2) and Sanger sequenced. *HvBDG1* (*HORVU.MOREX.r3.7HG0644300*) was sequenced in the original donor, *cer‐z.52* and eight other *cer‐z* alleles, as well as BW156 lines obtained from four sources (JHI seed store, USDA stocks, NordGen stocks and a JHI Lab stock from 2019), the *cer‐a* BW‐NIL BW406 and 65 *cer‐a* alleles, along with parental lines (Table S1). Whole‐genome sequencing of genomic DNA was performed on the Illumina NovaSeq platform. Raw reads were mapped and variants called as described in Liu *et al*. (2022). We filtered and retained high‐confidence models (Morex V2) containing predicted missense mutations in BW156.

### RNA isolation and gene expression analyses

All primers and reaction conditions are listed in Table S2. We mined the EoRNA barley gene expression database (Milne *et al*., 2021) and exported normalised expression. We analysed gene expression in caryopses by quantitative reverse transcription polymerisation chain reaction (qRT‐PCR) from Bowman caryopses at 3, 5, 7, 9 and 11 DPA (> 3 caryopses per individual, pooling three individuals per biological replicate). Samples were snap‐frozen in liquid nitrogen and stored at −70°C. We isolated RNA using TRIzol reagent (Sigma) as described in Patil *et al*. (2019), synthesised cDNA using the ProtoScript II First Strand cDNA Synthesis Kit (NEB, Ipswich, MA, USA) and performed qRT‐PCR using Roche FastStart Universal Probe Master (ROX) and gene‐specific primers for *HvBDG1 (HORVU.MOREX.r3.7HG0644300*), with *PPA* (*HORVU.MOREX.r3.5HG0522310*) and 26S (*HORVU.MOREX.r3.7HG0714100*) as controls.

RNA was isolated from tissue sections dissected from the second leaf at GS11; each leaf was dissected into three sections: the emerged portion of the leaf, a mid‐section 3 cm section immediately below the point of leaf emergence and a remaining section at the base of the leaf. Each section was harvested into four biological replicates each containing a pool of five individuals. RNA was also isolated from leaf sheaths at GS39 (*n* = 3) as in Liu *et al*. (2022) and caryopses (*n* = 3) at 5 and 9 DPA. RNA was isolated using the QIAGEN RNeasy Kit and cDNA synthesised using SuperScript IV VILO Master Mix (Invitrogen). qRT‐PCR was performed using SYBR Green PowerUp (Thermo Fisher Scientific) and gene‐specific primers for *HvBDG1*, with *PDPK1* (*HORVU7Hr1G096480*) and *Actin* (*HORVU5Hr1G039850*) as endogenous controls for leaf blade samples, and *PDPK1* and *TBL25* (*HORVU1Hr1G029950; HORVU.MOREX.r3.1HG0031980*) for grain samples. We also examined *HvCER‐U* in leaf sheaths with *Actin* as the control and validated RNA‐Seq using gene‐specific primers and *PDPK1* as the control. Relative expression was calculated using the $2^{-\Delta \Delta C_{t}}$ method (Pfaffl, 2001).

For *in situ* hybridisation, 5 and 9 DPA caryopses were fixed and sectioned as described in Campoli *et al*. (2024). Probe synthesis, slide processing and *in situ* hybridisation followed Gramma & Wahl (2023). Regions of *HvBDG1* (370 bp) and *HvWIN1* (400 bp) transcripts were amplified from Bowman cDNA and cloned into pCR‐Blunt (Thermo Fisher Scientific, Waltham, MA, USA) and pMini‐T 2.0 (NEB), respectively, in antisense orientation from the T7 promoter. Antisense templates including the T7 promoter were amplified from plasmid DNA for *HvBDG1* and *HvWIN1*. A *NUD* fragment (291 bp) was PCR amplified from Bowman cDNA using primers with an added T7 binding site. Sense templates were made as in Campoli *et al*. (2024). Templates were phenol‐chloroform purified and then digoxigenin‐labelled sense and antisense probes synthesised using T7 polymerase using the DIG RNA Labelling Kit (SP6/T7; Roche). Slides were imaged using a Zeiss Axioscan 7 Slide Scanner at 20× magnification.

### Phylogenetic analyses

HvBDG1 was used as a query for Blastp searches in Ensembl Plants (https://plants.ensembl.org/), FernBase (https://www.fernbase.org/), SolGenomics (https://solgenomics.net/), Phytozome (https://phytozome‐next.jgi.doe.gov/) and PLAZA Gymnosperms (https://bioinformatics.psb.ugent.be/plaza/versions/gymno‐plaza/). Full protein sequences from *Hordeum vulgare*, *Zea mays*, *Sorghum bicolor*, *Oryza sativa*, *Brachypodium distachyon*, *Triticum aestivum*, *Arabidopsis thaliana*, *Brassica rapa*, *Solanum tuberosum* and *Solanum lycopersicum* were aligned using Geneious 10.2.6 (https://www.geneious.com). Sequences were designated as motifs if at least two residues were 100% conserved within the alignment. Using Muscle (Edgar, 2004) to align sequences in Molecular Evolutionary Genetics Analysis 11 (Mega11; Tamura *et al*., 2021), evolutionary history was inferred using the maximum likelihood method and JTT matrix‐based model (Jones *et al*., 1992) with 500 bootstrap replications. BDG sequences from *Chlamydomonas reinhardtii*, *Chara braunii*, *Marchantia polymorpha*, *Physcomitrium patens* and *Amborella trichopoda* were extracted from Ensembl Plants and *Klebsormidium nitens* from the *Klebsormidium nitens* NIES‐2285 genome project (www.plantmorphogenesis.bio.titech.ac.jp/).

### Protein structure analyses

HvBDG1 protein secondary structure was initially predicted using Phyre2 in intensive mode (Kelley *et al*., 2015). Protein sequences for HvBDG1 (GenBank accession: KAI4977554.1) and the H407R mutant were submitted to the NCBI Conserved Domain Database (Marchler‐Bauer *et al*., 2015, 2017), InterProScan v.98.0 (Paysan‐Lafosse *et al*., 2023) and CDVist (Adebali *et al*., 2015) for the identification of conserved domains. Secondary and tertiary structural predictions were conducted using the Integrated Fold Recognition‐Tertiary Structure (IntFOLD‐TS) tool hosted by the IntFOLD7 server (McGuffin *et al*., 2019, 2023) utilising LocalColabFold 1.0.0 (Jumper *et al*., 2021; Mirdita *et al*., 2022) and trRosetta2 (Anishchenko *et al*., 2021), further refined using ReFOLD3 (Adiyaman & McGuffin, 2021) and assessed using ModFOLD9 (McGuffin *et al*., 2021). Structure quality was assessed with DeepUMQA‐X (Guo *et al*., 2022), stereochemistry by ProCheck (Laskowski *et al*., 2018) and structural flexibility determined using the CABS‐flex 2.0 server (Kuriata *et al*., 2018). Conservation scores were calculated with ConSurf (Ashkenazy *et al*., 2016) based on multiple sequence alignments of homologues identified via HMMER against UniRef90 (E‐value 1e^−3^; identity threshold: 35–95%), aligned using Mafft. Structural homologues were identified using FoldSeek (Van Kempen *et al*., 2023) and validated through TM‐alignment via the RCSB‐PDB pairwise structure alignment tool (Bittrich *et al*., 2024). All structural visualisations were rendered in PyMOL (Schrödinger LLC).

### Cellular localisation

*HvBDG1* CDS from Bowman was cloned into pK7WGR2 and pK7RWG2 to generate p35S‐driven constructs fused with red fluorescent protein (RFP) at the N‐terminus and C‐terminus, respectively. *Agrobacterium tumefaciens* strain GV3101 was transformed with the recombinant binary vector and cultures carrying each construct with p19 silencing suppressor at an OD_600_ of 0.01 in infiltration buffer (10 mM MES, 10 mM MgCl_2_ and 0.2 mM acetosyringone) used to infiltrate the youngest fully expanded leaves of *c*. 4‐wk‐old *Nicotiana benthamiana*. *Nicotiana benthamiana* plants were grown at 20°C under long day photoperiod (16 h : 8 h, light : dark). Leaves were harvested 48 or 72 h post infiltration, infiltrated with water and mounted on microscope slides. Abaxial epidermal cells were imaged using a Nikon A1R confocal laser scanning microscope (GFP and RFP excited at 488 nm and 561 nm, with emissions at 500–530 nm and 570–620 nm, respectively). RFP‐HvBDG1 was transiently expressed in *N*. *benthamiana* lines expressing CB28 (ER‐HDEL‐GFP, Haseloff *et al*., 1997; Ruiz *et al*., 1998) and Lti‐PM‐GFP (Kurup *et al*., 2005). RFP‐HvBDG1 was also co‐infiltrated with constructs expressing a Golgi body marker (ST‐YFP, Boevink *et al*., 1998) and two endosome markers (Ara6‐YFP and Ara7‐YFP, Ueda *et al*., 2001).

### Transcriptomics

RNA was assessed using a Bioanalyzer 2100 (Agilent Technologies). Library construction, sequencing, data preprocessing and differential expression analysis using the 3D RNA‐Seq App were performed as in Liu *et al*. (2022) with fold change cut‐off of log_2_ 0.5 defining differentially expressed genes (DEGs; *n* = 4, except Bowman 9 DPA, where *n* = 3). Gene Ontology (GO) enrichment analysis was performed using g:Profiler with a custom barley GO annotation, as described in Liu *et al*. (2022). Ontology categories retrieved using the Bioconductor package GO.db (Huber *et al*., 2015) and data plotted using ggplot2 (Wickham, 2016) in RStudio (Posit Team, 2025). Mapman enrichment analysis was performed using Mercator4 (Schwacke *et al*., 2019).

### Wheat *BDG1* and *WIN1* mutant identification, germplasm, genetic marker development and phenotyping

To identify wheat orthologues, the barley CDS from gene models *HORVU.MOREX.r3.7HG0644300* (*HvBDG1*) and *HORVU.MOREX.r3.6HG0578240* (*HvWIN1*) were used for Blastn searches of the reference genome of durum wheat (*T. turgidum* ssp. *durum* cv Svevo, assembly Svevo.v1; Maccaferri *et al*., 2019) using Ensembl Plants release 60 (Harrison *et al*., 2024). Exploiting the ethyl methanesulfonate (EMS) mutated Targeting Induced Local Lesions IN Genomes (TILLING) library and associated exome capture sequence data previously generated for tetraploid durum wheat cv Kronos (2*n* = 4*X* = 28, consisting of A and B subgenomes; Krasileva *et al*., 2017), mutants with premature stop or splice site mutations predicted to severely affect TdBDG1 or TdWIN1 homoeologue protein function were identified bioinformatically using Ensembl Plants, followed by manual inspection. Seed of TILLING lines were obtained from the SeedStor GeneBank (https://www.seedstor.ac.uk/) and grown using ‘Niab cereals soil mix’ supplied by ICL (https://icl‐growingsolutions.com/) in a naturally lit glasshouse supplemented with Sunblast LED lights (https://kroptek.com/) under a 16 h : 8 h, 20°C : 16°C, day : night regime. DNA was extracted from seedling leaves as in Fulton *et al*. (1995). Targeted mutations were confirmed and tracked using codominant PCR‐based Kompetitive allele‐specific (KASP) assays (https://3crbio.com/) using primers listed in Table S2. For each target gene, TILLING mutations on the A and B subgenome homoeologues were brought together via crosses, with resulting F_1_ grains selfed to generate F_2_ seed segregating for wild‐type (WT) vs mutant genotype at both homoeologues. Using the KASP assays, F_2_ individuals were identified as follows: (1) homozygous for the mutation in both homoeologues, (2) homozygous for the mutation in one homoeologue and homozygous WT for the other homoeologue, or (3) homozygous WT at both homoeologues. Leaf waxiness was phenotyped on F_2_ plants at the grain filling stage. Selected genotypes were grown from F_3_ seed in glasshouses at Dundee and wax extracted and quantified from leaf sheaths as described for barley. Photographs of leaf sheaths were taken from the same population 1 wk later *c*. 60 d after sowing.

## Results

### Variation in *HvBDG1* causes defective wax blooms

**Fig. 1:**
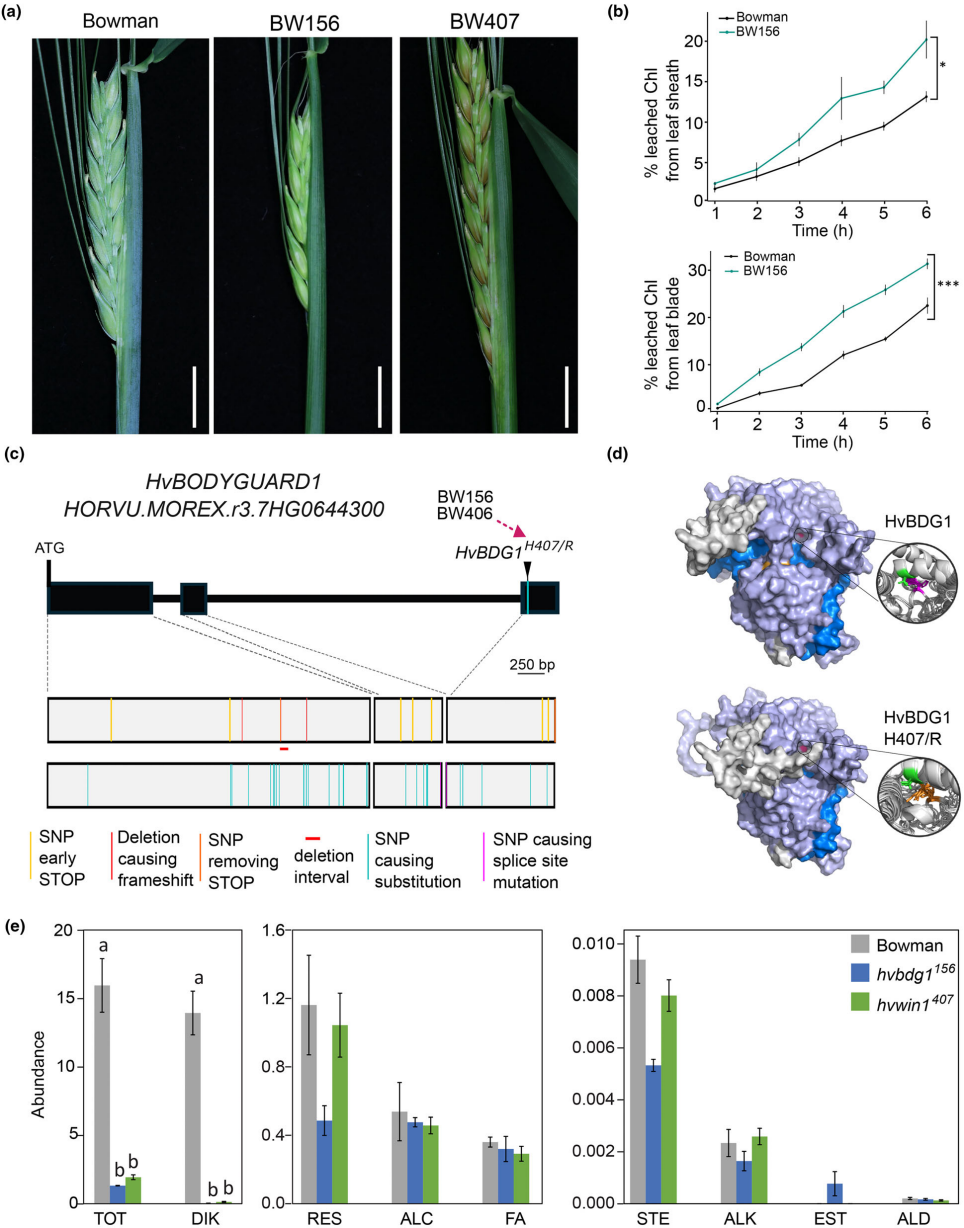
Cuticle defects result from defective *HvBDG1* alleles. (a) Barley (*Hordeum vulgare* L.) Bowman, BW156 and BW407 leaf sheaths and spikes. Bars, 1 cm. (b) Chl leaching of Bowman and BW156 detached leaf sheaths (Bowman, *n* = 3; BW156, *n* = 2) and leaf blades (*n* = 4/genotype). Error bars show SE. *, *P* < 0.05; ***, *P* < 0.001 (ANOVA followed by Tukey's HSD *post hoc* test). (c) *HvBDG1* gene model and allelic variation. Boxes represent exons, and horizontal lines represent introns. Gene model represents the full genomic sequence from the start codon to the stop codon. Vertical turquoise line represents A>G single‐nucleotide polymorphism (SNP) found in *hvbdg1* ^*156*^ and *hvbdg1* ^*406*^ and original mutants which leads to a H407/R amino acid substitution. Boxes underneath represent *HvBODYGUARD1* (*HvBDG1*) exons with coloured vertical lines representing changes to the HvBDG1 protein predicted from *cer‐a* alleles. Upper panel: vertical yellow lines indicate SNPs generating early stop codons, vertical red lines indicate deletions causing frameshifts, the horizontal red line indicates a deletion interval, and a vertical orange line refers to a SNP removing the stop codon. Lower panel: vertical turquoise lines indicate SNPs causing amino acid substitutions and vertical pink lines indicate SNPs causing splice site mutations. Bar, 250 bp. (d) HvBDG1 protein models where regions and domains are overlaid with an opaque surface. Top image is Bonus cultivar (parental wild‐type) and bottom image is HvBDG1 H407/R as found in *hvbdg1* ^*156*^. The alpha–beta hydrolase_1 domain shown in dark blue within a larger BDG1 domain containing hydrolase in pale blue. Four active site residues are situated at H225, S299, D448 and H476, shown in orange where S299, D448 and H476 form a catalytic triad and H225 is predicted to stabilise the interaction. Circle insets show H407 interacting with S368 in Bonus HvBDG1 while R407 does not interact with S368 in the H407/R mutant version preventing interactions between α‐helix 18 and 21. (e) Bar graphs showing soluble surface lipid classes extracted from leaf sheaths. Letters indicate significant differences within genotypes (*P* < 0.05; Tukey's HSD multiple comparison following one‐way ANOVA). *Y*‐axes indicate compound relative abundance mg^−1^ of sample fresh weight. Bars show the average with SD (*n* = 4/genotype). ALC, alcohols; ALD, aldehydes; ALK, alkanes; DIK, diketones; EST, esters; FA, fatty acids; RES, resorcinols; STE, sterols; TOT, total soluble lipid extract.

To learn more about cuticular specialisations in barley, we studied several *cer‐z* alleles described to have reduced wax blooms (Lundqvist & von Wettstein‐Knowles, 1962). We confirmed that BW‐NIL156 (BW156), a Bowman near‐isogenic line reportedly derived from the neutron radiation‐induced *cer‐z.52* allele in cv Bonus (Lundqvist & Franckowiak, 1997a), exhibited glossy leaf sheaths and spikes (Fig. 1a). BW156 leaf blades (*P* < 0.001) and sheaths (*P* < 0.05) leached Chl faster compared with Bowman (Fig. 1b). We genotyped BW156, Bowman and Bonus with the 50 k iSelect SNP array (Bayer *et al*., 2017), revealing an 898 Kbp Bonus introgression on the short arm of chromosome 7 (7HS) in BW156 (Fig. S2; Table S3). Comparing the Morex V1 genome assembly (Mascher *et al*., 2017) with the genome assemblies of cv Golden Promise (Schreiber *et al*., 2020) and Morex V2 (Monat *et al*., 2019) identified 16 high‐confidence gene models within the interval. Sequencing all 16 genes detected variation unique to BW156 in a single gene, *HORVU.MOREX.r3.7HG0644300*, which we name *HvBODYGUARD1* (*HvBDG1*) due to homology with *AtBDG1* (AT1G64670) which controls cuticular features in Arabidopsis (Kurdyukov *et al*., 2006a,b Jakobson *et al*., 2016). Compared with Bonus, *HvBDG1* in BW156 has a single nonsynonymous A to G DNA polymorphism predicted to change residue 407 in the 501 amino acid HvBDG1 protein from a histidine to an arginine (H407/R; Fig. 1c). Mapping whole‐genome sequencing reads from BW156, Bowman and Bonus to the Morex V2 genome assembly, followed by small (< 50 bp) and structural (≥ 50 bp) variant calling, identified the same A to G SNP in the *HvBDG1* gene (Table S4). BW406, another *cer* BW‐NIL derived from the radiation‐induced *cer‐a* mutant *glossy sheath3.i* (*gsh3.i*) generated in cv Mars (Lundqvist & Franckowiak, 1997b), phenocopied the reduced wax blooms of BW156 (Table S4) and showed an overlapping introgression with BW156 based on 50 k iSelect SNP genotyping of BW406 and Mars (Table S3). Sequencing *HvBDG1* in BW406 identified the same A to G SNP (Fig. 1c) as in BW156. Of 65 original *cer‐a* alleles available, 61 had mutations in *HvBDG1* (Fig. 1c; Table S1): 52 with severe disruptions on the predicted protein associated with glossy phenotypes, and nine with less disruptive effects associated with intermediate phenotypes (Table S4). Altogether, we identified 61 independent *cer‐a* alleles representing 42 unique variants within *HvBDG1*, suggesting that variation at *HvBDG1* underlies *cer‐a*. Surprisingly, the H407/R detected in BW156/BW406 mutation was observed in *cer‐a.1* generated in Maja Abed but not in the reported BW406 donor *gsh3.i*, which instead had a 8‐bp deletion leading to a frameshift at H407. Maja Abed is derived from Bonus (Zakhrabekova *et al*., 2023) and shares the same *HvBDG1* exon sequence as Bonus. We detected no changes in the *HvBDG1* CDS or 1400‐bp upstream of *HvBDG1* in the nine *cer‐z* allelic lines, including the original *cer‐z.52*, compared with parental backgrounds (data not shown). Independent stocks (in‐house, JHI, NordGen and USDA) showed identical *HvBDG1* variation in BW156 (Table S4). Altogether, these results indicate that BW156 contains an introgressed *cer‐a.1* allele, rather than *cer‐z.52*, and variation in *HvBDG1* underlies *cer‐a* alleles. Thus, we subsequently refer to BW156 as *hvbdg1* ^*156*^ and BW406 as *hvbdg1* ^*406*^.

BW407 closely resembled the glossy appearance of *hvbdg1* ^*156*^ (Fig. 1a). Our earlier work showed that leaf blades show increased Chl leaching in this line and that BW407 derives from an M54/R amino acid substitution in the predicted AP2 domain within *HvWIN1* (McAllister *et al*., 2022); we refer to this line as *hvwin1* ^*407*^. Total soluble surface lipids and wax were lower in *hvbdg1* ^*156*^ and *hvwin1* ^*407*^ leaf sheaths compared with Bowman, mostly due to reductions in β‐diketones (99.8 and 99.1%, respectively, *P* = 4.48 E^−12^; Fig. 1e,f; Table S5), while fatty acids also decreased in *hvbdg1* ^*156*^ and *hvwin1* ^*407*^, with most chain lengths reduced by 18.8–93.3% and the strongest reductions (70.0–93.3%) in longer chain fatty acids, C28 and C30 (*P* < 0.05; Fig. S3). By contrast, C26 alcohols increased in *hvbdg1* ^*156*^ and *hvwin1* ^*407*^ by 109 and 36%, respectively (*P* = 2.08 E^−04^). Leaf sheaths in both *hvbdg1* ^*156*^ and *hvwin1* ^*407*^ expressed less *CER‐U* compared with Bowman (Fig. S4), consistent with defective wax‐bloom metabolism. Taken together, both HvBDG1 and HvWIN1 promote deposition of β‐diketone wax blooms in barley.

### BDG gene family and protein modelling

To construct a BDG protein phylogeny, we identified BDG proteins in all selected angiosperms and a BDG protein in the terrestrial filamentous charophyte *Klebsormidium nitens*, but not in the chlorophyte algae *Chlamydomonas reinhardtii* or the charophyte *Chara braunii* (Braun's stonewort; Fig. S5; Table S6). We detected two other *BDG*‐like genes in barley, *HORVU.MOREX.r3.1HG0051940* and *HORVU.MOREX.r3.2HG0111190*, named here *HvBDG2* and *HvBDG4*, respectively (Fig. S5). HvBDG1 falls in a grass‐specific clade while HvBDG2 sits in a separate grass‐specific clade (Fig. S5), which may indicate that duplications generating BDG1‐like and BDG2‐like proteins occurred after the monocot and dicot divergence. By contrast, HvBDG4 groups in a clade containing monocots and dicots sequences (Fig. S5). The EoRNA barley gene expression database (Milne *et al*., 2021) revealed distinct *HvBDG* gene expression patterns with *HvBDG1* was most highly and broadly expressed, especially in epidermal cells and expanding floral organs (Fig. S6).

To identify conserved regions within BDG proteins, we aligned selected BDG homologues (Fig. S7). All BDG proteins have five highly conserved sequence motifs in the C‐terminal α/β hydrolase domain, a core structural architecture characteristic of the α/β hydrolase superfamily (Shaw *et al*., 2002): the oxyanion hole, type II β‐turn, a nucleophile elbow containing a catalytic serine and two additional catalytic residues – an aspartate (acidic) and a histidine (basic; Fig. S7; Motifs 6, 7, 8, 15 and 16, respectively; Table S7). BDG proteins also contain six completely conserved, novel sequence motifs within the α/β hydrolase domain: two corresponded to the structurally conserved α‐helix (Motif 9) and β‐sheet (Motif 10) following the nucleophilic elbow, and four situated in a potential lid domain (Motifs 11–14). Four motifs appear within the N‐terminal BDG domain (Fig. S7; Motifs 2–5; Kurdyukov *et al.*, 2006a), including a transmembrane domain predicted through Phyre2 (Powell *et al*., 2025) positioning an N‐terminus signal peptide in the apoplast. Many *cer‐a* substitution mutations occurred within the α/β hydrolase domain or the BDG domain, including the *hvbdg1* ^*156*^ allele H407/R substitution (Fig. S7; Table S4). Interestingly, HvBDG1 and all its cereal‐specific clade members have a highly conserved 11–14 amino acid N‐terminal motif (Motif 1) absent in other BDGs (Fig. S7). We generated structural models of the Bonus *HvBDG1* and *HvBDG1* ^*156*^ H407/R variants (Fig. 1d; model metrics in Notes S2 and Fig. S8). The cv Bonus HvBDG1 model exhibited a canonical α/β hydrolase core containing a central β‐sheet flanked by α‐helices and a catalytic triad, comprising S299, D448 and H476, as well as a V‐shaped lid domain made up of four α‐helices arranged over a moderately positively charged active site nestled within a hydrophobic pocket. The H407 residue resides beyond the N' terminus of α‐helix 21 and is stabilised with α‐helix 18 by an interaction between H407 and S368 (Fig. S8). The HvBDG1^156^ H407/R substitution replaces the last histidine in a conserved histidine‐threonine‐histidine sequence (Fig. S8) with an arginine not predicted to interact with S368. Consequently, α‐helices 18 and 21 would not stabilise each other, causing a local conformational rearrangement (Fig. 1d) with increased flexibility in the preceding 150 aa and decreased flexibility between 375 and 400 aa (Fig. S8). This may cause a compressed local area and lid conformation in HvBDG1^156^ H407/R, which could obstruct substrate access to the active site, consistent with *hvbdg1* ^*156*^ phenocopying deletion alleles (Table S4).

### Cellular localisation of HvBDG1

**Fig. 2:**
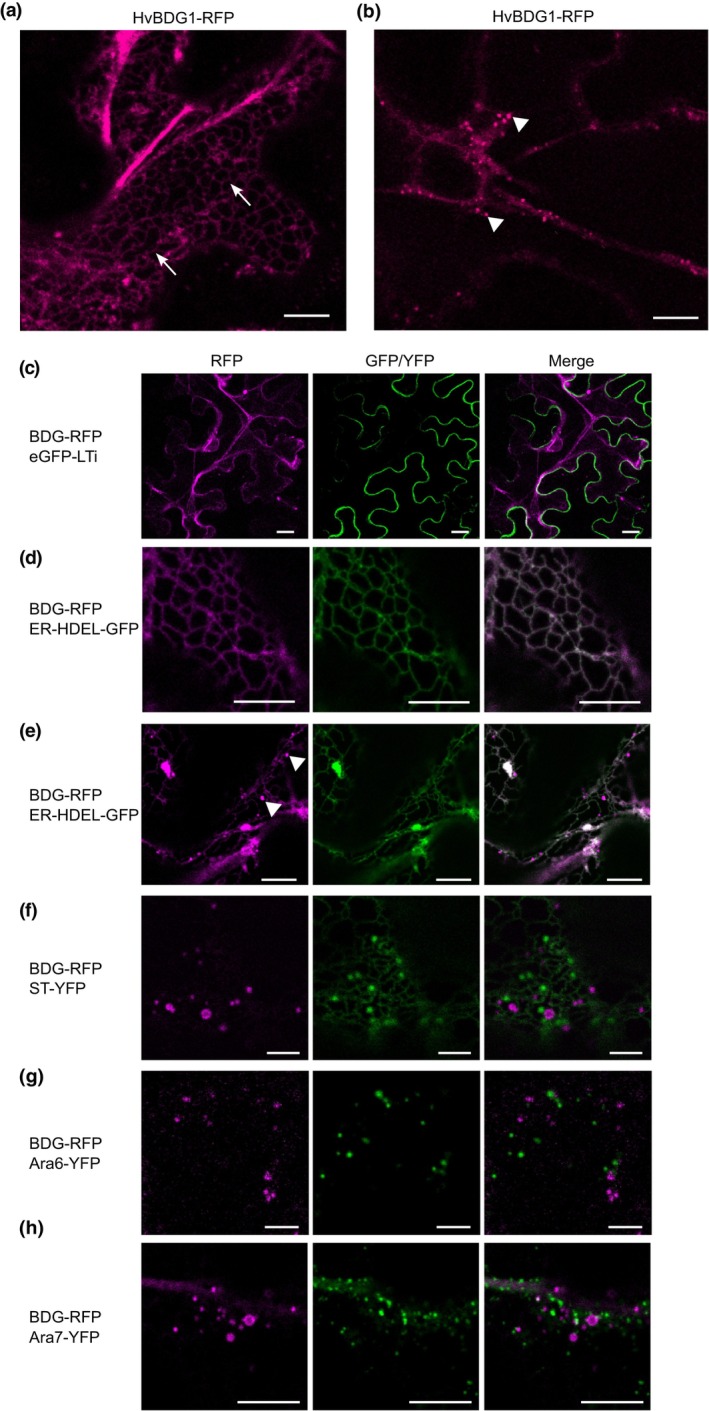
Tagged HvBDG1 protein localises to the endoplasmic reticulum and spherical mobile bodies. Localisation in infiltrated *Nicotiana benthamiana* leaves transiently expressing constructs as indicated. (a) shows leaves 2 d postinfiltration (DPI); all other panels show samples are 3 DPI. (a, b) Red fluorescent protein (RFP)‐HvBODYGUARD1 (HvBDG1). Reticulate structures characteristic of the endoplasmic reticulum (ER) indicated by arrows and spherical bodies by arrowheads. Bars, 10 μm. (c) RFP‐HvBDG1 shows limited overlap with the plasma membrane Lti‐PM‐GFP marker. Bars, 20 μm. (d) RFP‐HvBDG1 shows overlap with Green fluorescent protein (GFP) fused to an ER retention motif (ER‐HDEL‐GFP) in reticulate structures. Bars, 10 μm. (e) RFP‐HvBDG1 signal in mobile bodies (arrowheads) does not overlap with ER‐HDEL‐GFP. (f) Co‐transformation of RFP‐HvBDG1 and the Golgi marker ST‐YFP. Bars, 5 μm. (g) Co‐transformation of RFP‐HvBDG1 and the endosomal marker Ara6. Bars, 5 μm. (h) RFP‐HvBDG1 and the endosomal marker Ara7. Bars, 10 μm.

To explore the localisation pattern of HvBDG1, we transiently expressed an N‐terminally fused RFP‐HvBDG1 protein in *N*. *benthamiana*, detecting fluorescence within reticulate structures, characteristic of the endoplasmic reticulum (ER), and in mobile spherical bodies (Fig. 2a,b; Videos S1 and S2). HvBDG1 without the grass‐specific N‐terminus (RFP‐tHvBDG1) and C‐terminal fusions for each showed the same localisation (Fig. S9). Expression of RFP‐HvBDG1 in a line expressing the plasma membrane marker Lti‐PM‐GFP had overlap in small areas which may represent ER‐plasma membrane contact points (Fig. 2c), although the resolution cannot exclude ER or cytosol. Transient expression of RFP‐HvBDG1 in a line expressing an ER‐GFP lumen marker showed overlap in RFP and GFP signals (Fig. 2d), although not in the mobile bodies (Fig. 2e). The Golgi body marker ST‐YFP and YFP‐fused endosome markers, Ara6 and Ara7 did not co‐localise with RFP‐HvBDG1 (Fig. 2f–h), but since these only label a subset of endomembrane compartments, RFP‐HvBDG1 could localise to different endosomes. While we do not rule out overexpression effects, our data suggest that HvBDG1 travels through an unconventional secretory pathway associated with the ER and mobile bodies. We did not detect a signal in the outer cell wall as reported for AtBDG1 (Kurdyukov *et al*., 2006a).

### BDG1 and WIN1 promotion of the wax bloom is conserved in wheat

**Fig. 3:**
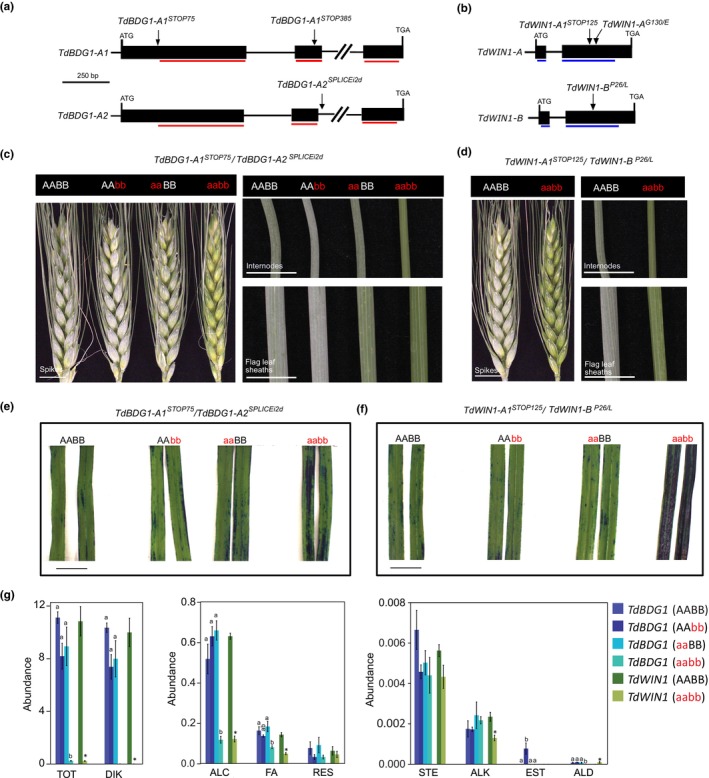
Durum wheat *TdBDG1* and *TdWIN1* TILLING mutants confirm conserved role in the control of epidermal wax bloom between wheat and barley. Exon/intron structure of durum wheat (*Triticum turgidum ssp. durum*) A‐ and B‐subgenome homoeologues of (a) *TdBODYGUARD1* (*HvBDG1*) and (b) *TdWAX‐INDUCER1* (*TdWIN1*). The locations of the selected TILLING mutants are indicated. The exon regions that encode for predicted protein domains in *TdBDG1* (alpha/beta hydrolase fold‐1 domain, pfam ID PF00561) and *TdWIN1* (AP2/ERF domain, Panther ID PTHR31194) are shown using red and blue horizontal bars, respectively. Examples of wax‐bloom phenotype observed in durum wheat spikes from F_2_ individuals carrying different wild‐type (WT) and mutant alleles at homoeologues of (c) *TdBDG1* and (d) *TdWIN1*. Bars, 1 cm. For *TdBDG1*, the allelic state at the A‐ and B‐subgenome homoeologue is indicated via uppercase (WT) or lowercase (mutant) letters, such that homozygous WT (AA or BB), homozygous mutant (aa or bb), and combinations of homozygous and WT (e.g. AAbb) alleles are indicated. For *TdWIN1*, only the double homozygous WT and double homozygous mutants are shown. (e, f) Detached second leaf blades from WT and mutant alleles at homoeologues of (e) *TdBDG1* and (f) *TdWIN1* were submerged in 0.05% Toluidine Blue (TB) for 5 h. Bars, 1 cm. For each genotype, allelic state at the A‐ and B‐subgenome homoeologue is indicated via uppercase (WT) or lowercase (mutant) letters, such that homozygous WT (AA or BB), homozygous mutant (aa or bb) and combinations of homozygous and WT (e.g. AAbb) alleles are indicated. Four biological replicates of each genotype were stained, with two representatives photographed to show adaxial and abaxial sides, respectively. (g) Bar graphs showing soluble surface lipid classes extracted from leaf sheaths. Letters (*TdBDG1*) and asterisks (*TdWIN1*) indicate significant differences within genotypes (*P* < 0.05; Tukey's HSD multiple comparison following one‐way ANOVA). *Y*‐axes indicate compound relative abundance/mg sample fresh weight. Bars show the average with SD (*n* = 4/genotype). ALC, alcohols; ALD, aldehydes; ALK, alkanes; DIK, diketones; EST, esters; FA, fatty acids; RES, resorcinols; STE, sterols; TOT, total soluble lipid extract.

We tested whether *BDG* and *WIN* genes promote wax blooms in other cereal species by developing durum wheat mutant lines. Durum is an allotetraploid species consisting of A and B subgenomes. For *HvBDG1* (located on the short arm of chromosome 7H at 18.1 Mb), collinearity between barley and wheat (e.g. Wang *et al*., 2015) expects the orthologous durum wheat homoeologues on the short arms of chromosomes 7A and 7B. Analysis of the durum reference genome found that while *TdBDG1‐A1* (*TRITD7Av1G017740*) is located at the expected location (chromosome 7A at 32.1 Mb), the expected 7B homoeologue actually resided on the long arm of chromosome 4A at 685.5 Mb (*TRITD4Av1G244120*, termed here *TdBDG1‐A2*), due to the chromosomal translocation T(4AL;7BS)1 present in both tetraploid (Dvorak *et al*., 2018) and hexaploid (Zhou *et al*., 2020) wheat (Notes S3). Thus, from an evolutionary perspective, *TdBDG1‐A2* represents the durum B subgenome homoeologue, rather than a *BDG1* paralogue. For *HvWIN1* (chromosome 6H at 187.9 Mb), orthologous durum homoeologues were identified at the expected collinear locations on the short arms of Chromosomes 6A (*TRITD6Av1G082070* at 204.1 Mb; *TdWIN1‐A*) and 6B (*TRITD6Bv1G086320* at 262.3 Mb; *TdWIN1‐B*). Next, we searched for TILLING mutants that disrupted the *TdBDG1* homoeologues. Two independent premature stop mutants were identified for *TdBDG1‐A1*: the *TdBDG1‐A1* ^*STOP75*^ allele, found in TILLING line Kronos3179, had a G225/A mutation in the CDS within exon‐1, resulting in a stop codon at amino acid residue 75 in the predicted protein, W75/*. The *TdBDG1‐A1* ^*STOP385*^ allele, found in TILLING line Kronos0599, had a mutation at position G1154/A in the CDS, leading to a stop codon at amino acid 385 in the predicted protein, W385/* (Fig. 3a). We also identified another premature stop mutant in *TdBDG1‐A2* (*TdBDG1‐A2* ^*SPLICEi2d*^) that was predicted to result from a GT/AT mutation in the intron‐2 canonical splice donor motif, resulting in a nonsense mutation in the subsequent codon that terminated the predicted protein at amino acid residue 320. All three mutants resulted in either complete (*TdBDG1‐A1* ^*STOP75*^ and *TdBDG1‐A1* ^*STOP385*^) or partial (*TdBDG1‐A2* ^*SPLICEi2d*^) loss of the alpha/beta hydrolase fold‐1 predicted protein domain (pfam ID PF00561; Fig. 3a). For *TdWIN1*, three TILLING mutants were selected, all containing mutations in the AP2/ERF predicted protein domain (Panther ID PTHR31194): premature stop mutant *TdWIN1‐A* ^*STOP125*^ (Kronos3416) and two missense mutations with SIFT scores = 0 at conserved amino acid residues on the A (*TdWIN1‐A* ^*G130/E*^, Kronos2646) and B (*TdWIN1‐B* ^*P26/A*^, Kronos2314) homoeologues (Fig. 3b). Mutated homoeologues were combined by crossing to generate F_1_ hybrids, followed by marker‐assisted selection in F_2_ progeny derived from selfed F_1_ plants. For *TdBDG1*, homozygous *TdBDG1‐A1* and *TdBDG1‐A2* double mutants (combinations *TdBDG1‐A1* ^*STOP75*^ */TdBDG1‐A2* ^*SPLICEi2d*^ and *TdBDG1‐A1* ^*STOP385*^ */TdBDG1‐A2* ^*SPLICEi2d*^) lacked the wax blooms on the spike, stem and leaf sheath observed in WT segregants (Figs 3c, S10). Leaf blades from both *TdBDG1* and *TdWIN1* double mutants showed increased permeability to Toluidine Blue (Fig. 3e,f). The ability to generate a more quantitative effect was shown in different BDG mutant homoeologue combinations. The single *TdBDG1‐A2* ^*SPLICEi2d*^ mutant did not show visible defects, the single *TdBDG1‐A1* ^*STOP75*^ mutant's appearance suggested possible reduced wax, while combining both mutated homoeologues caused a visible waxless phenotype (Fig. 3c). Quantification of soluble surface lipids revealed severe reductions in β‐diketones (−99.9%, *P* = 7.54E^−06^) and hydroxy β‐diketones (−100%, *P* < 0.05) while only hydroxy β‐diketones were statistically decreased in both *TdBDG1* single mutant combinations (−10 and −24%, respectively, for *TdBDG1‐A1* and *TdBDG1‐A2* single mutants, *P* < 0.05), which may underlie the intermediate phenotypes (Figs 3g, S11; Table S5). Like *TdBDG1*, all *TdWIN1* double mutants lacked wax blooms (Figs 3d, S9) with corresponding strong decreases in β‐diketones (−99.9%, *P* < 0.0001) and hydroxy β‐diketones (−99.9%, *P* < 0.05; Fig. 3g,h; Table S5). Similar to barley, loss of TdBDG1 and TdWIN1 reduced fatty acids by 56% (*P* < 0.001) and 69% (*P* < 0.001), respectively, with most chain lengths between C20 and C30 decreased. Mutant lines also showed reduced alcohols, with C24 particularly affected (−92%, *P* < 0.0001 and −99%, *P* < 0.0001, respectively in *TdBDG* and *TdWIN1* double mutants) in contrast to the barley mutants where alcohols increased (Figs 1f, S11). Taken together, *BDG1* and *WIN1* control of surface lipid composition in leaf sheaths appears mostly conserved, with some species‐specific differences.

### Variation in *HvBDG1* or *HvWIN1* causes grain skinning in barley and regulates surface features

**Fig. 4:**
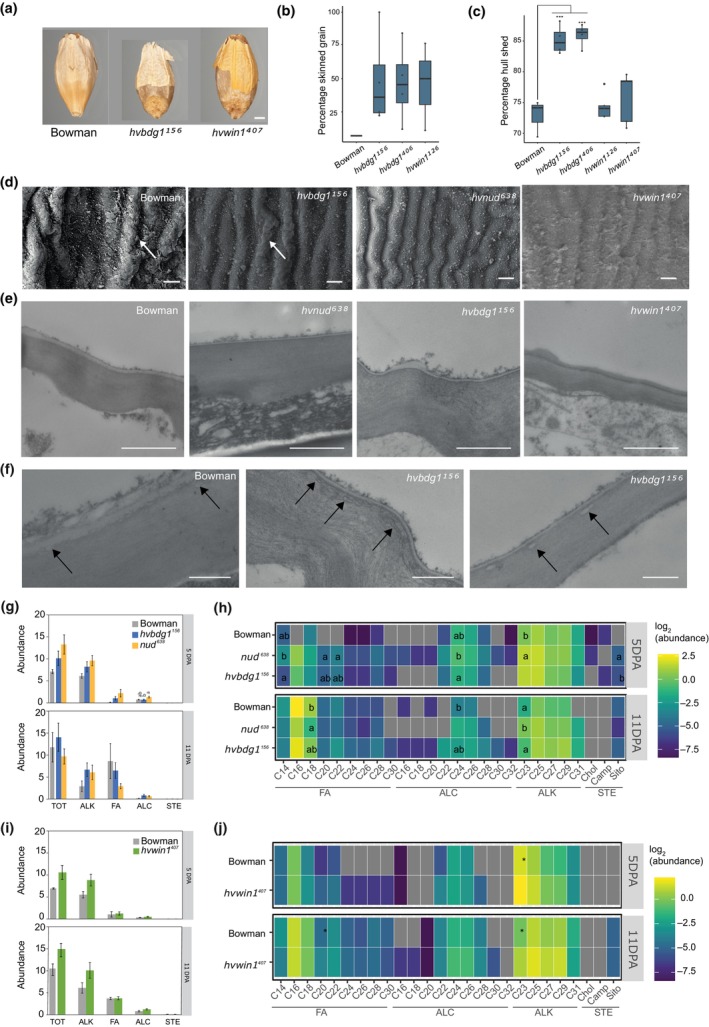
Barley *HvBDG1*, *HvWIN1* and *NUD* regulate grain skinning and features of the caryopsis pericarp cuticle. (a) Photos of hull adhesion in in barley (*Hordeum vulgare* L.) Bowman, *hvbdg1* ^*156*^ and *hvwin1* ^*407*^ manually threshed grain. Dorsal side. Bars, 0.05 cm. (b) Manual assay indicates percentage of grain showing any skinning. Bowman (*n* = 1 grain pooled from three individuals), *hvbdg1* ^*156*^ (*n* = 4), *hvbdg* ^*406*^ (*n* = 4) and *hvwin1* ^*126*^ (*n* = 3). (c) Debranner assay showing extent of hull separation in Bowman (*n* = 3), *hvbdg1* ^*156*^ (*n* = 4), *hvbdg1* ^*406*^ (*n* = 4) and *hvwin1* ^*126*^ (*n* = 4), *hvwin1* ^*407*^ (*n* = 5). Boxes represent the interquartile range with the horizontal line representing the median. Whiskers represent the upper and lower quartiles plus 1.5 times the interquartile range. Points represent individual replicates. ***, *P* < 0.001 (ANOVA followed by Tukey's HSD *post hoc* test). (d) Scanning electron microscopy (SEM) of 11 d postanthesis (DPA) caryopsis (hulls removed) pericarp surfaces in Bowman, *nud* ^*638*^, *hvbdg1* ^*156*^ and *hvwin1* ^*407*^. Arrows indicate accumulating cuticular material on ridges. Bars, 1 μm. (e) Transmission electron microscopy (TEM) of the outer pericarp in sections made from 11 DPA caryopses (hulls removed) in Bowman, *nud* ^*638*^, *hvbdg1* ^*156*^ and *hvwin1* ^*407*^. Bars, 1 μm. (f) Higher magnification TEM of (e) samples showing the pericarp cell wall–cuticle interface. White arrows indicate electron‐lucent globules merging between cell wall and cuticle in Bowman while black arrows indicate electron‐lucent globules remaining within the cell wall of *hvbdg1* ^*156*^. Bars, 500 nm. Images are representative of at least three biological replicates. (g–j) Bar graphs (g, h) showing soluble surface lipid classes and heatmaps (i, j) showing chain lengths extracted from 5 and 11 DPA caryopses (hulls removed). (*n* = 3/genotype). Letters (*P* < 0.05; Tukey's HSD multiple comparison following one‐way ANOVA) and asterisks (*, *P* < 0.05; one‐way ANOVA) indicate significant differences within genotypes within one stage *Y*‐axes (g, h) indicate compound relative abundance/caryopsis. Bars show the average with SD. Bar (h, j) indicates compound relative abundance/caryopsis on a log_2_ scale. ALC, alcohols; ALK, alkanes; FA, fatty acids; STE, sterols; TOT, total soluble lipid extract.

We noticed that *hvbdg1* ^*156*^ and *hvwin1* ^*407*^ grain showed skinning following manual threshing. 49, 47 and 46% of *hvbdg1* ^*156*^, *hvbdg1* ^*406*^ and *hvwin1* ^*126*^ grain, respectively, shed hulls compared with only 7% of Bowman (Fig. 4a,b). Debranning led to *hvbdg1* ^*156*^ and *hvbdg1* ^*406*^ grain losing 85 and 86% more hulls by weight, respectively, compared with 73% in Bowman (*P* < 0.001; Fig. 4c). Neither *hvwin1* ^*126*^ nor *hvwin1* ^*407*^ showed statistical differences in hull weight lost compared with Bowman after debranning, suggesting that rougher abrasion may mask differences observed by manual threshing (Fig. 4c). Together, HvBDG1 appears essential for strong hull to caryopsis adhesion while HvWIN1 may make a smaller contribution.

Deletion of *NUD* results in complete loss of hull adhesion, attributed to a loss of an adherent cementing layer on the pericarp cuticle (Gaines *et al*., 1985; Taketa *et al*., 2008). We hypothesised that *HvWIN1* and *HvBDG1* function downstream of NUD so that the intermediate hull adhesion phenotype (skinning) reflects a partially defective NUD‐driven pathway(s). To explore this, we examined pericarp surfaces in *hvbdg1* ^*156*^ and *hvwin1* ^*407*^ skinning alleles, the naked BW638 (*nud* ^*638*^) allele and Bowman. Before hull adhesion at 7 DPA, SEM of Bowman pericarp epidermal cells showed a mixture of smooth surfaces, narrow perpendicular nanoridges and wider longitudinal ridges. Surfaces in *hvwin1* ^*407*^ resembled Bowman, *hvbdg1* ^*156*^ had some instances of crooked longitudinal ridges and *nud* ^*638*^ had cells with perpendicular striations (Fig. S12). During hull adhesion at 11 DPA, the waxy plaques decorated Bowman were infrequent and smaller in *hvbdg1* ^*156*^ and *hvwin1* ^*407*^, and not observed in *nud* ^*638*^ (Fig. 4d). TEM revealed a thick cuticle in the 7 DPA Bowman pericarp epidermis with underlying, darkly stained electron‐lucent globules and fibrillar extrusions extending from the cell wall, features not present in the thinner cuticle of *nud* ^*638*^ (Fig. S13). By 11 DPA, Bowman's thickened pericarp cuticles showed outer lamellations and a merged cell wall–cuticle interface containing electron‐lucent globules (Fig. 4e,f). By contrast, pericarp cuticles in 11 DPA *nud* ^*638*^ samples were thin with a sharp, smooth electron‐dense boundary to the outer cell wall (Fig. 4e). Pericarp cuticles in *hvbdg1* ^*156*^ and to a lesser extent *hvwin1* ^*407*^ were thin with thickened regions and smooth, electron‐dense cell wall–cuticle interfaces (Fig. 4e). Interestingly, the *hvbdg1* ^*156*^ pericarp cell wall showed electron‐lucent globules, which did not merge with the cuticle (Fig. 4e,f). Together, NUD, HvBDG1 and HvWIN1 may regulate cuticular thickening, epicuticular plaques and the cuticle–cell wall interface. Consistent with our hypothesis, these features were most disrupted in *nud* ^*638*^ and less so in *hvbdg1* ^*156*^ and *hvwin1* ^*407*^.

### Roles of *HvBDG1* , *HvWIN1* and *NUD* in pericarp surface composition

We extracted soluble surface lipids and cutin monomers from developing caryopses and hulls of Bowman, *nud* ^*638*^ and *hvbdg1* ^*156*^ and of Bowman and *hvwin1* ^*407*^. Because these extractions were performed in separate experiments, the resulting data were analyzed independently (Fig. 4g–j; Table S8a–d). Data from Bowman and *nud* ^*638*^ (Campoli *et al*., 2024) are used here for comparison. In all genotypes, alkanes dominated soluble surface lipids extracted from caryopses at 5 DPA, followed by fatty acids and alcohols, with only small amounts of sterols (Fig. 4g,i). We detected increased shorter length alkanes and fatty acids in NILs compared with Bowman. In 5 DPA caryopses, C23 alkanes accumulated 177% more in *nud* ^*638*^ (*P* < 0.001), 122% more in *hvbdg1* ^*156*^ (*P* < 0.001) and 58% more in *hvwin1* ^*407*^ than Bowman (*P <* 0.05; Fig. 4h; Table S8). Although decreased in all genotypes by 11 DPA, C23 alkanes remained 279% higher in *nud* ^*638*^ (*P* = 0.003) and 113% higher in *hvwin1* ^*407*^ (*P <* 0.05) compared with Bowman (Fig. 4j; Table S8). Also, at 5 DPA, C14 fatty acids were increased 333% in *nud* ^*638*^ compared with Bowman (*P <* 0.05; Fig. 4h; Table S8a), and C16, C20 and C22 fatty acids, undetected at 5 DPA in Bowman caryopses, were evident in *nud* ^*638*^ and *hvbdg1* ^*156*^ (Fig. 4h; Table S8a). We compared 5 DPA to 11 DPA for changes coincident with hull adhesion (Table S8a). Bowman, *hvbdg1* ^*156*^ and *hvwin1* ^*407*^ samples showed increased total fatty acids from 5 DPA to 11 DPA (+244, +486 and +192%, respectively, *P* < 0.05), a change not observed in *nud* ^638^ (Table S8a), while total soluble surface lipids were unchanged across genotypes. Alcohols, in particular C24, decreased in Bowman (−82%, *P* < 0.01), much less so in *nud* ^*638*^ (−47%, *P* < 0.05), and increased in *hvwin1* ^*407*^ (+123%, *P* < 0.05) between 5 and 11 DPA, although C24 was increased 338% in *nud* ^*638*^ compared with Bowman at 11DPA (*P <* 0.05; Table S8a; Fig. 4h). Sitosterol also increased by 215% in *nud* ^*638*^ compared with Bowman (*P =* 0.037; Table S8a). Consistent with Campoli *et al*. (2024), the major cutin components were mono and di‐hydroxy C16 and C18 acids, with a prevalence of C16 moiety, followed by the C18 mono and di‐hydroxy acids (Fig. S14; Table S8c). Other than more ωOH C16 (ω‐hydroxyhexadecanoic acid) in *hvbdg1* ^*156*^ at 5 DPA compared with Bowman (+44%, *P <* 0.05), all lines showed a similar composition and increased these components between 5 and 11 DPA (Fig. S14; Table S8c). As expected from their glossy phenotypes, soluble surface lipids from hull extracts were decreased, mostly due to loss of β‐diketones and hydroxy β‐diketones in *hvbdg1* ^*156*^ and *hvwin1* ^*407*^ compared with Bowman (Fig. S15; Table S8b). Compared with Bowman, alcohols, alkanes and fatty acids in *hvbdg1* ^*156*^ and *hvwin1* ^*407*^ shifted from longer to shorter chain lengths, also seen in *nud* ^*638*^ (Fig. S15; Table S8b). Major cutin components in hulls at 11 DPA were aromatics followed by ωOH C16, with no differences between genotypes (Fig. S14; Table S8d). Taken together, NUD, HvBDG1 and HvWIN1 control the deposition and wax chain length profiles on the pericarp while HvBDG1 and HvWIN1 promote β‐diketone deposition on hulls.

### HvWIN1 and NUD function regulates *HvBDG* expression

**Fig. 5:**
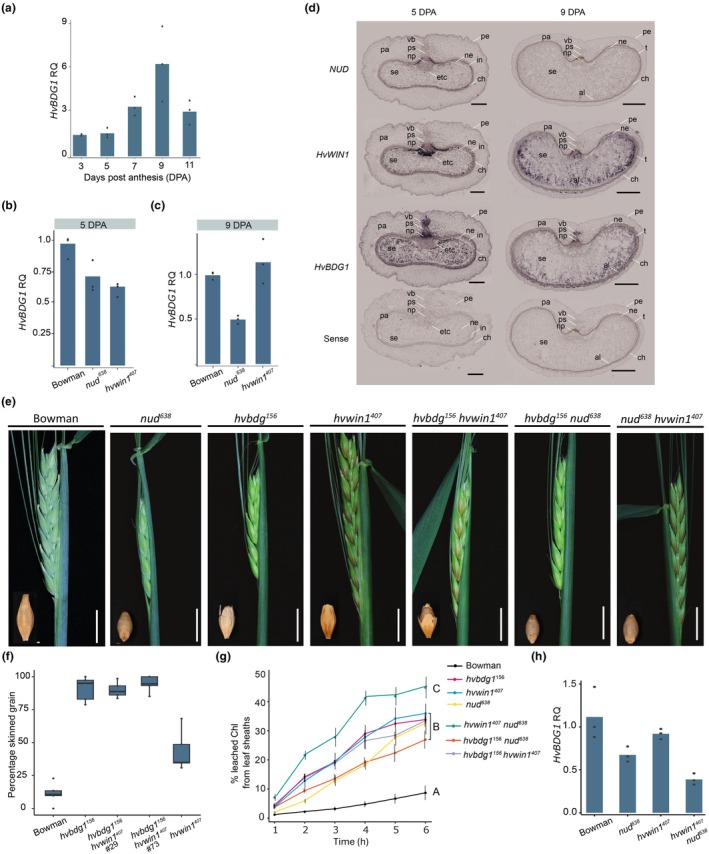
*HvBDG1*, *HvWIN1* and *NUD* expression and interaction. (a–c) Quantitative reverse transcription polymerisation chain reaction (qRT‐PCR) of *HvBODYGUARD1* (*HvBDG1*) transcripts in barley (*Hordeum vulgare* L.). Bar indicates average and individual replicates plotted by points. (a) Developmental time course of Bowman caryopses 3–11 d postanthesis (DPA). (b) 5 DPA in Bowman, *hvwin1* ^*407*^ and *nud* ^*638*^ caryopses, and (c) 9 DPA in Bowman, *hvwin1* ^*407*^ and *nud* ^*638*^ caryopses. *n* = 3. (d) RNA *in situ* hybridisation of caryopses sectioned from Bowman at 5 and 9 DPA. Sections hybridised with antisense probes specific for *NUDUM* (*NUD*), *HvWAX‐INDUCER1* (*HvWIN1*) and *HvBDG1* transcripts, or a nonspecific sense probe as indicated. Labels are al, aleurone; ch, pericarp chlorenchyma; etc, endosperm transfer cells; n, nucellus; np, nucellar projection; pa, pericarp parenchyma; pe, pericarp epidermis; ps, pigment strand; t, testa. Bars, 200 μm (5 DPA) and 500 μm (9 DPA). (e) Leaf sheaths, spikes and manually threshed grain (inset) shown for Bowman, *nud* ^*638*^, *hvbdg1* ^*156*^, *hvwin1* ^*407*^, *hvbdg1* ^*156*^ *hvwin1* ^*407*^ double mutant, *hvbdg1* ^*156*^ *nud* ^*638*^ double mutant and *nud* ^*638*^ *hvwin1* ^*407*^ double mutant. Bar, 1 cm. Dorsal side shown. Bar, 0.05 cm. (f) Skinning index of Bowman, single (*hvbdg1* ^*156*^, *hvwin1* ^*407*^) and double mutants (*hvbdg1* ^*156*^ *hvwin1* ^*407*^). Boxes represent the interquartile range with the horizontal line representing the median. Whiskers represent the upper and lower quartiles plus 1.5 times the interquartile range. Points represent individual replicates, *P* < 0.001 (ANOVA and Tukey's HSD), *n* = 5. (g) Chl leaching assays of leaf sheaths from Bowman, single mutants (*hvbdg1* ^*156*^, *hvwin1* ^*407*^, *nud* ^*638*^) and double mutants (*hvwin1* ^*407*^ *nud* ^*638*^, *hvbdg1* ^*156*^ *nud* ^*638*^ and *hvbdg1* ^*156*^ *hvwin1* ^*407*^). Different letters indicate significantly different groups, *P* < 0.0001 (ANOVA and Tukey's HSD). Error bars indicate SE (*n* = 4/genotype). (h) qRT‐PCR of *HvBDG1* transcripts in Bowman, *hvwin1* ^*407*^, *nud* ^*638*^ and *hvwin1* ^*407*^ *nud* ^*638*^ from second leaf blade base. Bar indicates average and individual replicates plotted by points. *n* = 3/genotype.

We examined *HvBDG1* expression in developing caryopses by qRT‐PCR. Bowman caryopses show peak *HvBDG1* expression coincident with the first signs of hull adhesion at 9 DPA (Fig. 5a). Selecting 5 DPA caryopses, we detected 70 and 60% *HvBDG1* expression in *nud* ^*638*^ and *hvwin1* ^*407*^ compared with Bowman, respectively (Fig. 5b), while at 9 DPA, *HvBDG1* expression declined 50% in *nud* ^*638*^, with little change in *hvwin1* ^*407*^ (Fig. 5c), suggesting both HvWIN1 and NUD influence *HvBDG1* expression early in grain development with NUD more important later. We next examined spatial expression using RNA *in situ* hybridisation and detected *NUD* transcripts in integuments and the nucellar epidermis, most strongly next to the nucellar projection, as well as faintly in the outer pericarp, vascular bundle and pigment strand at 5 DPA (Fig. 5d). *HvWIN1* and *HvBDG1* transcripts also accumulated in integuments and nucellar tissues at 5 DPA, with additional strong signals for *HvWIN1* in the lower and *HvBDG1* in the upper nucellar projection as well as in the pigment strand and vascular bundle (Fig. 5d). By 9 DPA, the nucellar epidermis compresses against the testa formed through integument maturation. At 9 DPA, *HvWIN1* and *HvBDG1* transcripts accumulated in endosperm and the nucellar projection with *HvBDG1* still detected in the pigment strand as well as the aleurone; *NUD* was not detected (Fig. 5d). We did not detect testa signal at 9 DPA for any probe, potentially due to testa pigmentation. Thus, *HvWIN1*, *NUD* and *HvBDG1* expression overlaps in the integuments, nucellar epidermis and the pericarp epidermis.

### *HvWIN1* and *NUD* independently control cuticle properties

We generated double mutants to investigate genetic relationships amongst *HvBDG1*, *HvWIN1* and *NUD*. *hvbdg1* ^*156*^ *nud* ^*638*^ and *nud* ^*638*^ *hvwin1* ^*407*^ double mutants formed naked grain resembling *nud* ^*638*^, while leaf sheaths in *hvbdg1* ^*156*^ *nud* ^*638*^ and *nud* ^*638*^ *hvwin1* ^*407*^ were similarly glossy to *hvbdg1* ^*156*^ and *hvwin1* ^*407*^ parents, supported by equivalent reductions in β‐diketones (Figs 5e, S16; Table S5). The *hvbdg1* ^*156*^ *hvwin1* ^*407*^ double mutant skinned equivalently to *hvbdg1* ^*156*^ while *hvwin1* ^*407*^ skinned intermediate to *hvbdg1* ^*156*^ and Bowman grain (*P* < 0.001; Fig. 5f), suggesting that HvBDG1 may act downstream but still functions to some degree in *hvwin1* ^*407*^, consistent with qRT‐PCR (Fig. 5b). While *nud* ^*638*^ does not show a wax‐bloom phenotype or reduced β‐diketones (Figs 5e, S16; Table S5), *nud* ^*638*^ leaf sheaths showed increased permeability (Fig. 5g). Combining *nud* ^*638*^ with *hvwin1* ^*407*^ increased sheath permeability significantly compared with either parent (*P* < 0.0001, Fig. 5g), suggesting additive effects. The double *nud* ^*638*^ *hvwin1* ^*407*^ mutant showed increased leaf blade permeability, while single mutants showed no change compared with Bowman (*P =* 0.038; Fig. S17). *HvWIN1* expression levels were unchanged in *nud* ^*638*^ leaf blades and vice versa, so NUD and HvWIN1 likely do not regulate each other's expression (Fig. S18). We separated second leaf blades into basal younger sections and middle maturing sections. *HvBDG1* transcripts accumulated to 80 and 70% of Bowman levels in *hvwin1* ^*407*^ and *nud* ^*638*^ basal leaves, respectively (Fig. 5h), while mid‐sections accumulated *HvBDG1* mRNA to 60% of Bowman levels in *hvwin1* ^*407*^ with no change in *nud* ^*638*^ (Fig. S19). We observed greater suppression of *HvBDG1* in the *nud* ^*638*^ *hvwin1* ^*407*^ double mutant leaf bases (Fig. 5h), suggesting that both factors may directly or indirectly control *HvBDG1* expression independently. In sum, expression and genetic analyses suggest that HvWIN1 and NUD may independently control hull adhesion and leaf cuticular integrity, with HvBDG1 working downstream.

### HvWIN1 and NUD control overlapping and distinct transcriptomes in developing grain

**Fig. 6:**
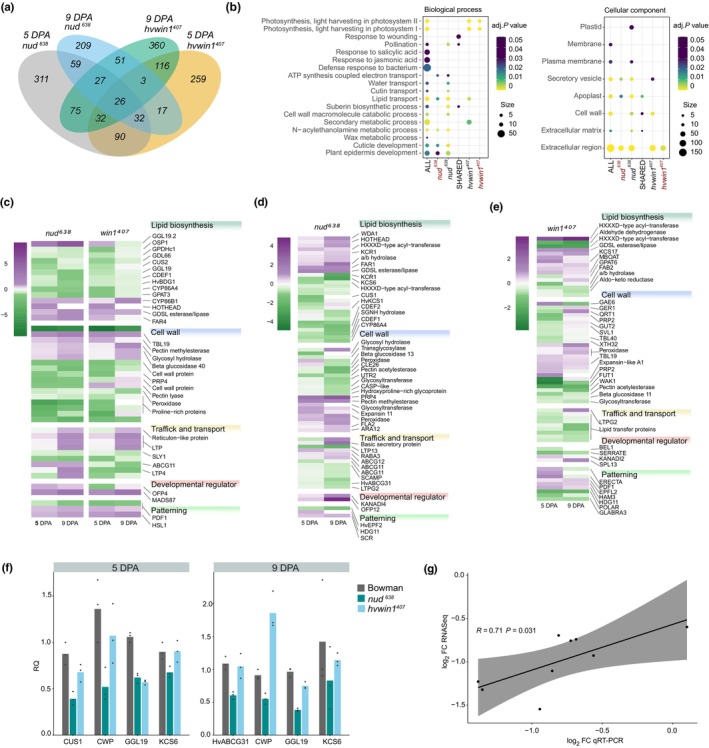
RNA‐Seq reveals shared and distinct gene expression patterns associated with HvWIN1 and NUD function. (a) Venn diagram describing the overlap between differentially expressed genes (DEGs) in barley (*Hordeum vulgare* L.) between 5 and 9 d postanthesis (DPA) and between in *nud* ^*638*^ and *hvwin1* ^*407*^ compared with Bowman. (b) Gene Ontology (GO) enrichment analyses. Left panel shows bubble plot for enriched GO biological processes and right panel shows enriched GO cellular components. The black font *nud* ^*638*^ and *hvwin1* ^*407*^ shows GO terms enriched in the entire DEG list for that genotype while the red font *nud* ^*638*^ and *hvwin1* ^*407*^ shows GO terms enriched in DEGs unique to that genotype. Shared reflects GO enrichment for DEGs found in both genotypes at any stage while the All category reflects the entire dataset. (c–e) Heat maps showing log^2^ fold change in DEG expression grouped by association with lipid biosynthesis, cell wall, traffic and transport, developmental regulator or epidermal development using a colour scale from upregulated (purple) to downregulated (green). (c) shared DEGs in *nud* ^*638*^ and *hvwin1* ^*407*^, (d) unique to *nud* ^*638*^ compared with Bowman and (e) unique *hvwin1* ^*407*^ compared with Bowman. *n* = 4 for all genotypes except for Bowman at 9 DPA, where *n* = 3. (f, g) RNA‐Seq validation by quantitative reverse transcription polymerisation chain reaction (qRT‐PCR) of representative DEGs in *nud* ^*638*^ and *hvwin1* ^*407*^ shown as (f) relative quantity (RQ) compared with Bowman (*n* = 3; bar indicates average and individual replicates plotted by points) and (g) Pearson's correlation coeficient between log_2_ FC values from RNAseq vs qRT‐PCR. Shaded regions represent the 95% confidence intervals.

To learn how NUD and HvWIN1 influence global gene expression during grain development, we performed RNA‐Seq on Bowman, *nud* ^*638*^ and *hvwin1* ^*407*^ caryopses at 5 DPA and 9 DPA. Comparing to Bowman, we identified 579 and 735 DEGs in *nud* ^*638*^ and *hvwin1* ^*407*^, respectively, with 353 DEGs shared between genotypes (Fig. 6a; Table S9). GO enrichment on all DEGs combined revealed enrichment in photosynthesis, defence and hormone responses, as well as cutin and lipid transport, wax metabolism, and cuticle and epidermal development (Fig. 6b; Table S10). DEGs in *nud* ^*638*^ were similarly enriched except for photosynthesis while those unique to *nud* ^*638*^ were enriched for lipid transport, cuticle development and epidermal development. By contrast, DEGs responsive to HvWIN1 loss of function were enriched for lipid transport, secondary metabolic process and photosynthesis, with DEGs specific to *hvwin1* ^*407*^ enriched only for photosynthesis. Shared DEGs between *nud* ^*638*^ and *hvwin1* ^*407*^ were enriched for response to wounding, possibly reflecting feedback due to cuticle defects. DEGs were also enriched for terms relating to extracellular region, apoplast and cell wall, as well as secretory vesicles. Mapman categories (Table S11) supported GO enrichment.

We examined individual DEGs associated with lipid biosynthesis, cell wall remodelling, trafficking and transport, as well as developmental regulation and patterning (Fig. 6c–e; Notes S4). We detected genes associated with wax and cutin biosynthesis, lipid transport and cell wall remodelling commonly misregulated (Fig. 6c). Downregulated genes included *CYP86A4* (*HORVU.MOREX.r3.2HG0188260*) required for cutin biosynthesis (Li‐Beisson *et al*., 2009), *CUTIN SYNTHASE2* (*CUS2*; *HORVU.MOREX.r3.6HG0629070*) required for cutin polymerisation and nanoridges in Arabidopsis and tomato (Yeats *et al*., 2012; Hong *et al*., 2017; Sagado *et al*., 2020), and *GDSL occluded stomatal pore1* (*OSP1*; *HORVU.MOREX.r3.5HG0468830*) required for wax biosynthesis (Tang *et al*., 2020). DEGs unique to *nud* ^*638*^ at either stage (Fig. 6d) included enzymes crucial for fatty acyl elongation, modification and transport, such as downregulated *HvKCS1* (*HORVU.MOREX.r3.4HG0392320*), which encodes a β‐ketoacyl‐CoA synthase whose defective alleles underlie *cer‐zh* (Li *et al*., 2018) and *KCS6* (*HORVU.MOREX.r3.7HG0670360*), consistent with fewer fatty acids in *nud* ^*638*^, upregulated fatty acyl‐CoA reductase *FAR1* (*HORVU.MOREX.r3.7HG0722250*) whose Arabidopsis homologue synthesises primary alcohols (Domergue *et al*., 2010), and which may contribute to C24 alcohol increases in *nud* ^*638*^, and decreased lipid transfer protein *LTPG2* (*HORVU.MOREX.r3.3HG0296370*), linked to cuticular wax accumulation in Arabidopsis (Kim *et al*., 2012). We also detected decreases in *CUS1* (*HORVU.MOREX.r3.5HG0486520*), *HvABCG31/Eibi1* (*HORVU.MOREX.r3.3HG0240110*), encoding a cutin transporter (Chen *et al*., 2011), and cell wall genes, such as peroxidases, glycosyltransferases and pectin acetylesterases. We detected upregulation of two *HOTHEADs* (*HORVU.MOREX.r3.5HG0475790* and *HORVU.MOREX.r3.2HG0186860*) associated with cutin synthesis in other species (Kurdyukov *et al*., 2006b; Xu *et al*., 2017). DEGs unique to *hvwin1* ^*407*^ represented pathways akin to those in *nud* ^*638*^ (Fig. 6e), including *LTPG2* (*HORVU.MOREX.r3.4HG0352050*) and acyltransferase *GPAT6* (*HORVU.MOREX.r3.4HG0340350*) required for cutin synthesis in tomato fruit cuticles (Petit *et al*., 2016). Also downregulated were *FAB2* (*HORVU.MOREX.r3.5HG0457600*), a gene important for embryonic cuticle in Arabidopsis (Kazaz *et al*., 2020), and *SERRATE*, important for RNA signalling and enhancing the *atbdg* phenotype (Voisin *et al*., 2009). We detected no increase in *NUD* levels in *hvwin1* ^*407*^ but a modest 1.4‐fold increase in *HvWIN1* in *nud* ^*638*^ at 9 DPA (*P =* 0.005; Table S9). Consistent with qRT‐PCR (Fig. 5b,c), HvBDG1 transcripts were 70% lower in both *hvwin1* ^*407*^ and *nud* ^*638*^ at 5 DPA and reduced 80% and 50% in *hvwin1* ^*407*^ and *nud* ^*638*^ at 9 DPA (Table S7). qRT‐PCR of representative genes from key enriched categories (Fig. 6f,g) correlated with RNA‐Seq (*R* = 0.71, *P* = 0.031). In sum, NUD and HvWIN1 function affects expression of many common genes, including *HvBDG1* and those involved in cell wall and cuticle formation, as well as distinct genes possibly relevant to different spatial expression patterns and/or levels of each SHN.

## Discussion

We report the first characterisation of a *BDG*‐like gene outside the dicots, revealing *HvBDG1* as essential for cuticular specialisations and cuticle integrity in barley, and demonstrate conservation of leaf roles in wheat.

Initially described as essential for cuticular integrity in Arabidopsis, we show defective *BDG1* alleles in barley and wheat cause leaf tissues to leach Chl more quickly or be more readily stained by toluidine blue, normally interpreted as reflecting impaired cuticle integrity (Kurdyukov *et al*., 2006a). While we detected lower wax loads, the first Arabidopsis *bdg* (*atbdg*) alleles were described to accumulate more epicuticular waxes and cutin (Kurdyukov *et al*., 2006a), while another *atbdg* allele was later shown to have no change in wax load, higher cutin load in mature leaves but lower cutin monomer levels in young leaves (Jakobson *et al*., 2016). We did not detect changes in cutin load in caryopses or hulls of the *hvbdg1* ^*156*^ allele, although we do not preclude that *hvbdg1* ^*156*^ could impact leaf cutin load. We also did not detect an extracellular localisation for HvBDG1, as shown for AtBDG1 and relevant to its proposed extracellular role in cutin polymerisation (Kurdyukov *et al*., 2006a; Jakobson *et al*., 2016). Rather, we showed HvBDG1 localisation to the ER and mobile bodies which could suggest an intracellular role modifying cuticular components enroute from the ER to the plasma membrane/cell wall, which if defective impacts cuticular organisation, as suggested by caryopsis TEM where HvBDG1 function appears important for cuticular thickening and merging of electron‐dense globules from the cell wall to cuticle.

While we do not know the exact molecular function executed by HvBDG1, our data suggest both SHNs in barley, HvWIN1 and NUD, act upstream of *HvBDG1* expression to control cuticle integrity. SHN‐responsive *BDG1* expression in multiple species, along with BDG and SHN co‐expression in land plants (Kong *et al*., 2020), suggests the SHN‐BDG module's control of cuticle properties regulating integrity is deeply conserved. However, since *nud* ^*638*^ *hvwin1* ^*407*^ cuticles are more permeable than *hvbdg1* ^*156*^ mutants (Figs 5g, S17), other genes besides *HvBDG1* contribute to SHN‐mediated cuticle integrity. These genes could include other *HvBDG*s (Figs S5, S6). Redundancy between *HvBDG*s may underlie *hvbdg1* ^*156*^'s mild phenotypes compared with the severe fusion, deformation, dwarfism and cutin load changes in *atbdg1* mutants (Kurdyukov *et al*., 2006a; Jakobson *et al*., 2016). Our evidence also suggests that NUD is not strictly sub‐functionalised to grain (as suggested in Taketa *et al*., 2008) with its new role in the leaf possibly relevant to drought tolerance in naked varieties, which all derive from a deletion of *NUD*, a focus of renewed interest as feedstock for malting (Meints & Hayes, 2019).

Our data also support that the SHNs and BDGs are deployed for cuticular specialisations in barley. Altogether, NUD and HvWIN1 are essential regulators for two late stage and agronomically relevant cuticular elaborations, the wax bloom and hull adhesion, respectively, but retain functional, independent overlap in leaf cuticle integrity and hull adhesion, with all functions involving upregulation of *HvBDG1* and other direct or indirect targets, shared and distinct, depending on the stage and tissue (Fig. S20). Coupled with our previous report of skinning in wax‐bloom defective alleles of *HvGDSL1* (Campoli *et al*., 2024), our work demonstrates that multiple loci influence wax bloom and hull adhesion, suggesting that breeders should routinely examine for co‐regulation of these traits.

Identifying both HvWIN1 and HvBDG1 as essential for complete hull adhesion marks the most significant advance in our understanding of this trait since *NUD* was identified (Taketa *et al*., 2008). We showed that *HvWIN1*, *NUD* and *HvBDG1* are co‐expressed in integuments, nucellar epidermis and the pericarp epidermis – all maternal tissues with cuticles (Freeman & Palmer, 1984) that undergo profound changes (Briggs, 1978). We show that the 7 DPA pericarp epidermis had cuticular ridges which by 11 DPA were decorated with thick cuticular plaques or deposits. Cuticular ridges on Arabidopsis petals help these organs slide by neighbouring organs (Li‐Beisson *et al*., 2009; Hong *et al*., 2017). Pericarp ridges in barley could similarly help the caryopsis slide past encasing hulls during grain lengthening and widening but later alter to promote adhesion during grain filling. In Arabidopsis, SHN knockdown reduced ridges and lowered expression of *AtBDG3* (Shi *et al*., 2011), while overexpression caused ectopic ridges and excess cuticular components deposition (Aharoni *et al*., 2004), suggesting that SHN dosage is critical. This may explain why *NUD* overexpression in rice did not cause adherent grain since *NUD* expression was not maintained in maturing rice caryopses (Kakeda *et al*., 2011). The wax bloom is also a late stage cuticular modification of a pre‐existing cuticle. We suggest barley co‐opted the grass‐specific duplication of separate NUD and HvWIN1 clades to generate high, sustained and localised doses of SHN activity to specialise cuticles later in development.

## Competing interests

None declared.

## Author contributions

SMM, CC, LR, RW and JC designed the research. TM, CC, LL, TC, ARP, RH, JS, ME, AI, YR, MN and SRF performed the research. TM, CC, LL, TC, JC and MB analysed experimental data. VW, MB, MS and LM provided tools to help perform the research and analyses. SMM wrote the manuscript with large contributions from TM, JC, TC and SRF. All authors had input into the manuscript. TM and CC contributed equally to this work.

## Disclaimer

The New Phytologist Foundation remains neutral with regard to jurisdictional claims in maps and in any institutional affiliations.

## Supporting information


**Fig. S1** Mature barley grain anatomy.
**Fig. S2** Mapping the introgression locus of BW156/*hvbdg1* ^*156*^.
**Fig. S3** Heat maps showing surface lipid chain lengths extracted from leaf sheaths (*n* = 4/genotype) from cv Bowman, and the Bowman near‐isogenic line mutants *hvbdg* ^*156*^ (BW156) and *hvwin1* ^*407*^ (BW407).
**Fig. S4** *Cer‐U* qRT‐PCR expression in the mid flag leaf sheath region of Bowman near‐isogenic lines (BW‐NILs) *hvbdg* ^*156*^ (BW156) and *hvwin1* ^*407*^ (BW407), expressed as relative quantity (RQ).
**Fig. S5** Phylogenetic relationship of the BDG family.
**Fig. S6** Expression profiles of *HvBDG* genes in barley cultivar Morex.
**Fig. S7** BDG protein motifs.
**Fig. S8** Protein modelling of HvBDG1.
**Fig. S9** Localisation patterns of N‐terminally and C‐terminally tagged HvBDG1 constructs.
**Fig. S10** Durum wheat stem and sheath phenotypes in *TdBDG1* and *TdWIN1* mutants.
**Fig. S11** Heat maps showing surface lipid chain lengths extracted from leaf sheaths.
**Fig. S12** Cuticular ridges on barley caryopses.
**Fig. S13** Bowman caryopsis cuticle is thicker at 7 d postanthesis (DPA).
**Fig. S14** Cutin monomers from barley hull and caryopses during adhesion.
**Fig. S15** Quantitative wax load of barley hulls.
**Fig. S16** Quantitative wax load of barley leaf sheaths.
**Fig. S17** Chl leaching in leaf blades of wild‐type Bowman, *hvbdg1* ^*156*^, *hvwin1* ^*407*^, *nud* ^*638*^ and double mutants.
**Fig. S18** NUD and HvWIN1 expression measured by qRT‐PCR in developing second leaf blades of barley cv Bowman, *hvnud* ^*638*^ and *hvwin1* ^*407*^, expressed as relative quantity (RQ).
**Fig. S19** *HvBDG1* expression measured by qRT‐PCR in mid‐leaf blade sections of barley.
**Fig. S20** Regulatory relationships between barley SHINE transcription factors, HvBDG1 and surface features.
**Notes S1** Detailed lipid compound analyses.
**Notes S2** Detailed validation and characterisation of HvBDG1 protein models.
**Notes S3** Identification of durum wheat BDG1.
**Notse S4** Differentially expressed genes in mutants compared with Bowman.

**Table S1** Barley (*Hordeum vulgare* L.) germplasm.
**Table S2** Primers used in this study.
**Table S3** Genotyping data of Bowman, Bonus, Mars, BW156 and BW406 using barley 50 k iSelect SNP chip.
**Table S4** HvBDG1 allele resequencing in *cer‐a/gsh3* mutants.
**Table S5** Leaf sheath surface lipids extracted from barley and wheat genotypes: Bowman, *hvbdg1* ^*156*^, *nud* ^*638*^, *hvwin1* ^*407*^ and *hvbdg1* ^*156*^ *nud* ^*638*^, *nud* ^*638*^ *hvwin1* ^*407*^, *hvbdg1* ^*156*^ *nud* ^*638*^ and durum wheat TILLING lines.
**Table S6** Gene models used in angiosperm phylogenetic tree.
**Table S7** HvBODYGUARD1 protein motifs.
**Table S8** Caryopsis surface lipids extracted from barley (*Hordeum vulgare* L.) caryopses of Bowman, *hvbdg1* ^*156*^, *nud* ^*638*^ and *hvwin1* ^*407*^; hull surface lipids extracted from barley (*Hordeum vulgare* L.) Bowman, *hvbdg1* ^*156*^, *nud* ^*638*^ and *hvwin1* ^*407*^; cutin components extracted from caryopses of barley (*Hordeum vulgare* L.) Bowman, *hvbdg1* ^*156*^, *nud* ^*638*^ and *hvwin1* ^*407*^; (d) Cutin components extracted from hulls of barley (*Hordeum vulgare* L.) extracted from hulls of Bowman, *hvbdg1* ^*156*^, *nud* ^*638*^ and *hvwin1* ^*407*^.
**Table S9** Differentially expressed genes resolved from RNA‐seq of carysopses from barley (*Hordeum vulgare* L.) Bowman, nud638 and hvwin1407 RNA‐seq of Bowman, *nud* ^*638*^ and *hvwin1* ^*407*^ caryopses.
**Table S10** Gene Ontology (GO) enrichment of differentially expressed genes resolved from RNA‐seq of carysopses from barley (Hordeum vulgare L.) Bowman, *nud* ^*638*^ and *hvwin1* ^*407*^.
**Table S11** MapMan categories of differentially expressed genes resolved from RNA‐seq of carysopses from barley (Hordeum vulgare L.) Bowman, *nud* ^*638*^ and *hvwin1* ^*407*^.

**Video S1** HvBDG1‐RFP localisation.

**Video S2** HvBDG1‐RFP and ER‐HDEL‐GFP localisation.
Please note: Wiley is not responsible for the content or functionality of any Supporting Information supplied by the authors. Any queries (other than missing material) should be directed to the *New Phytologist* Central Office.

## Data Availability

The data that support the findings of this study are available in the Supporting Information of this article described as Figs S1–S20, Notes S1–S4 and Tables S1–S11. All raw RNA‐Seq and WGS data from this study have been deposited at the European Nucleotide Archive (ENA) under project accession PRJEB111569. WGS read data for cultivars Bowman and Bonus were deposited as part of a previous study and are available at the ENA under accessions ERR9880841 and ERR9880842, respectively. Other data supporting the findings of this study can be accessed at https://ics.hutton.ac.uk/eorna/index.html. Sequences for gene models are found in Ensembl using the gene model IDs provided in this paper: barley gene models, Hordeum_vulgare – Ensembl Genomes 62 (https://plants.ensembl.org/Hordeum_vulgare/Info/Index); and durum wheat models, Triticum_turgidum – Ensembl Genomes 62 (https://plants.ensembl.org/Triticum_turgidum/Info/Index).
