# Supplementary material for: Barley HvBODYGUARD1 controls cuticular specialisations regulated by SHINE transcription factors
Source: New Phytol. 2026 May 27;251(3):1188–208. doi: 10.1111/nph.71287 (PMC13326517; doi:10.1111/nph.71287)
Supplement: Supplementary file 1 — Fig. S1 Mature barley grain anatomy. Fig. S2 Mapping the introgression locus of BW156/hvbdg1 156 . Fig. S3 Heat maps showing surface lipid chain lengths extracted from leaf sheaths (n = 4/genotype) from cv Bowman, and the Bowman near‐isogenic line mutants hvbdg 156 (BW156) and hvwin1 407 (BW407). Fig. S4 Cer‐U qRT‐PCR expression in the mid flag leaf sheath region of Bowman near‐isogenic lines (BW‐NILs) hvbdg 156 (BW156) and hvwin1 407 (BW407), expressed as relative quantity (RQ). Fig. S5 Phylogenetic relationship of the BDG family. Fig. S6 Expression profiles of HvBDG genes in barley cultivar Morex. Fig. S7 BDG protein motifs. Fig. S8 Protein modelling of HvBDG1. Fig. S9 Localisation patterns of N‐terminally and C‐terminally tagged HvBDG1 constructs. Fig. S10 Durum wheat stem and sheath phenotypes in TdBDG1 and TdWIN1 mutants. Fig. S11 Heat maps showing surface lipid chain lengths extracted from leaf sheaths. Fig. S12 Cuticular ridges on barley caryopses. Fig. S13 Bowman caryopsis cuticle is thicker at 7 d postanthesis (DPA). Fig. S14 Cutin monomers from barley hull and caryopses during adhesion. Fig. S15 Quantitative wax load of barley hulls. Fig. S16 Quantitative wax load of barley leaf sheaths. Fig. S17 Chl leaching in leaf blades of wild‐type Bowman, hvbdg1 156 , hvwin1 407 , nud 638 and double mutants. Fig. S18 NUD and HvWIN1 expression measured by qRT‐PCR in developing second leaf blades of barley cv Bowman, hvnud 638 and hvwin1 407 , expressed as relative quantity (RQ). Fig. S19 HvBDG1 expression measured by qRT‐PCR in mid‐leaf blade sections of barley. Fig. S20 Regulatory relationships between barley SHINE transcription factors, HvBDG1 and surface features. Notes S1 Detailed lipid compound analyses. Notes S2 Detailed validation and characterisation of HvBDG1 protein models. Notes S3 Identification of durum wheat BDG1. Notse S4 Differentially expressed genes in mutants compared with Bowman. [file NPH-251-1188-s002.docx]

## *New Phytologist* Supporting Information

Article title: **Barley BODYGUARD1 contributes to cuticular specialisations regulated by SHINE transcription factors**

Authors: Trisha McAllister, Chiara Campoli, Linsan Liu**,** Tansy Chia, S. Ronan Fisher, Richard Horsnell, Alan R. Prescott, Jennifer Shoesmith, Mhmoud Eskan, Alasdair Iredale, Mirjam Nuter, Luke Ramsay, Micha M. Bayer, Linda Milne, Miriam Schreiber, Yogeswari Rajarathinam, Vanessa Wahl, Robbie Waugh, James Cockram, and Sarah M. McKim

Article acceptance date: 28 April 2026

The following Supporting Information is available for this article:

**Supporting Information**

**Fig. S1** **Mature barley grain anatomy**

**Fig. S2** **Mapping the introgression locus of BW156/*hvbdg1^156^***

**Fig. S3** **Heat maps showing surface lipid chain lengths extracted from leaf sheaths (n=4/genotype) from *cv.* Bowman, and the Bowman near isogenic line mutants *hvbdg^156^* (BW156) and *hvwin1^407^* (BW407).**

**Fig. S4** ***Cer-U* qRT-PCR expression in the mid flag leaf sheath region of Bowman near-isogenic lines (BW-NILs) *hvbdg^156^* (BW156) and *hvwin1^407^* (BW407), expressed as relative quantity (RQ).**

**Fig. S5** **Phylogenetic relationship of the BDG family.**

**Fig. S6** **Expression profiles of *HvBDG* genes in barley cultivar Morex**

**Fig. S7** **BDG protein motifs**

**Fig. S8** **Protein modelling of HvBDG1**

**Fig. S9** **Localisation patterns of N-terminally and C-terminally tagged HvBDG1 constructs.**

**Fig. S10** **Durum wheat stem and sheath phenotypes in *TdBDG1*and *TdWIN1*mutants.**

**Fig. S11** **Heat maps showing surface lipid chain lengths extracted from leaf sheaths.**

**Fig. S12** **Cuticular ridges on barley caryopses**

**Fig. S13** **Bowman caryopsis cuticle is thicker at 7 days post anthesis (DPA)**

**Fig. S14** **Cutin monomers from barley hull and caryopses during adhesion**

**Fig. S15** **Quantitative wax load of barley hulls**

**Fig. S16** **Quantitative wax load of barley leaf sheaths**

**Fig. S17** **Chlorophyll leaching in leaf blades of wild-type Bowman, *hvbdg1^156^*, *hvwin1^407^*, *nud^638^*, and double mutants**

**Fig. S18** **NUD and HvWIN1** **expression measured by qRT-PCR in developing second leaf blades of barley cv. Bowman, *hvnud^638^* and *hvwin1^407^*, expressed as relative quantity (RQ)**

**Fig. S19** ***HvBDG1* expression measured by qRT-PCR in mid-leaf blade sections of barley**

**Fig. S20** **Regulatory relationships between barley SHINE transcription factors, HvBDG1 and surface features**

**Table S1 Barley (*Hordeum vulgare* L.) germplasm**

**Table S2 Primers used in this study**

**Table S3 Genotyping data of Bowman, Bonus, Mars, BW156 and BW406 using barley 50k iSelect SNP chip**

**Table S4 HvBDG1 allele resequencing in *cer-a/gsh3* mutants**

**Table S5 Leaf sheath surface lipids extracted from barley and wheat genotypes: Bowman, *hvbdg1^156^*, *nud^638^*, *hvwin1^407^*, and *hvbdg1^156^ nud^638^*, *nud^638^ hvwin1^407^*, *hvbdg1^156^ nud^638^* and durum wheat TILLING lines**

**Table S6 Gene models used in angiosperm phylogenetic tree**

**Table S7 HvBODYGUARD1 protein motifs**

**Table S8a Caryopsis surface lipids extracted from barley (*Hordeum vulgare* L.) caryopses of Bowman, *hvbdg1^156^*, *nud^638^* and *hvwin1^407^***

**Table S8b Hull surface lipids extracted from barley (*Hordeum vulgare* L.) Bowman, *hvbdg1^156^*, *nud^638^* and *hvwin1^407^***

**Table S8c Cutin components extracted from caryopses of barley (*Hordeum vulgare* L.) Bowman, *hvbdg1^156^*, *nud^638^* and *hvwin1^407^***

**Table S8d Cutin components extracted from hulls of barley (*Hordeum vulgare* L.) extracted from hulls of Bowman, *hvbdg1^156^*, *nud^638^* and *hvwin1^407^***

**Table S9 Differentially expressed genes resolved from RNAseq of caryopses from barley (*Hordeum vulgare* L.) Bowman, nud638 and hvwin1407 RNAseq of Bowman, *nud^638^* and *hvwin1^407^* caryopses**

**Table S10 Gene Otology (GO) enrichment of differentially expressed genes resolved from RNAseq of caryopses from barley (Hordeum vulgare L.) Bowman, *nud^638^* and *hvwin1^407^***

**Table S11 MapMan Categories of differentially expressed genes resolved from RNAseq of caryopses from barley (Hordeum vulgare L.) Bowman, *nud^638^* and *hvwin1^407^***

**Note S1** **Detailed lipid compound analyses**

**Note S2** **Detailed validation and characterisation of HvBDG1 protein models.**

**Note S3** **Identification of durum wheat BDG1**

**Note S4** **Differentially expressed genes in mutants compared to Bowman**

**Video S1 HvBDG1-RFP localisation**

**Video S2 HvBDG1-RFP and ER-HDEL-GFP** **localisation**

**Fig. S1 Mature barley (*Hordeum vulgare* L.) grain anatomy**. Filial tissues including the aleurone, endosperm and embryo are surrounded by maternal tissue layers. The outer aleurone (peach colour) is a filial layer surrounding the endosperm. Prominent maternal tissues include the protective seed coat or testa (purple colour) which is further surrounded by the pericarp (green colour). Adhesion between the pericarp and floret hulls (brown colour) occurs at a boundary called the cementing layer (black colour). The pericarp epidermis and seed coats each have their own cuticle.


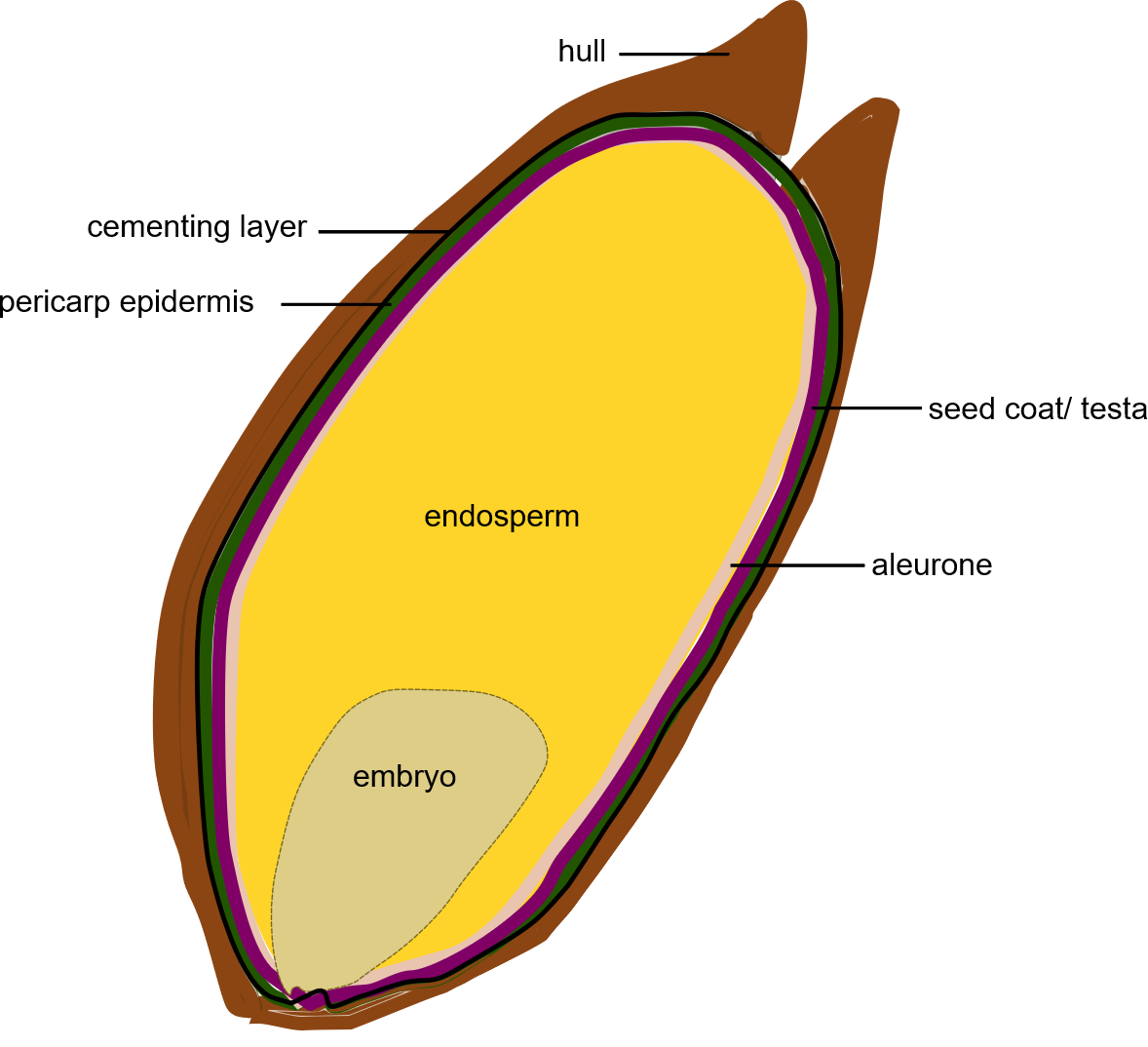


**Fig. S2 Mapping the introgression locus of BW156/*hvbdg1^156^***. Schematic representation of *Hordeum vulgare* L. chromosome 7H of BW156 highlighting the introgression from *cv.* Bonus in red. In the blow-up, polymorphic markers (red for Bonus, black for *cv.* Bowman) from the 50k iSelect SNP chip are shown with their positions based on the Morex v1 genome assembly


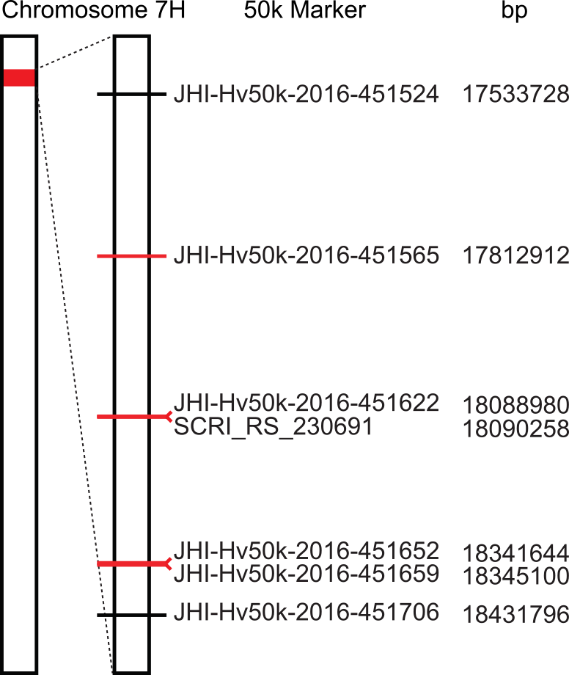


**Fig. S3 Heat maps showing surface lipid chain lengths extracted from leaf sheaths. Results from** *Hordeum vulgare* L. from *cv.* Bowman, and the Bowman near isogenic line mutants *hvbdg^156^* (BW156) and *hvwin1^407^* (BW407). Letters indicate significant differences within genotypes (P < 0.05; Tukey’s HSD multiple comparison following one-way ANOVA). Scale bar indicates compound relative abundance/mg of sample fresh weight on a log_2_ scale. (n = 4/ genotype) FA, fatty acids; ALC, alcohols; ALK, alkanes; RES, resorcinols; ALD, aldehydes; STE, sterols; EST, esters; DIK, diketones.


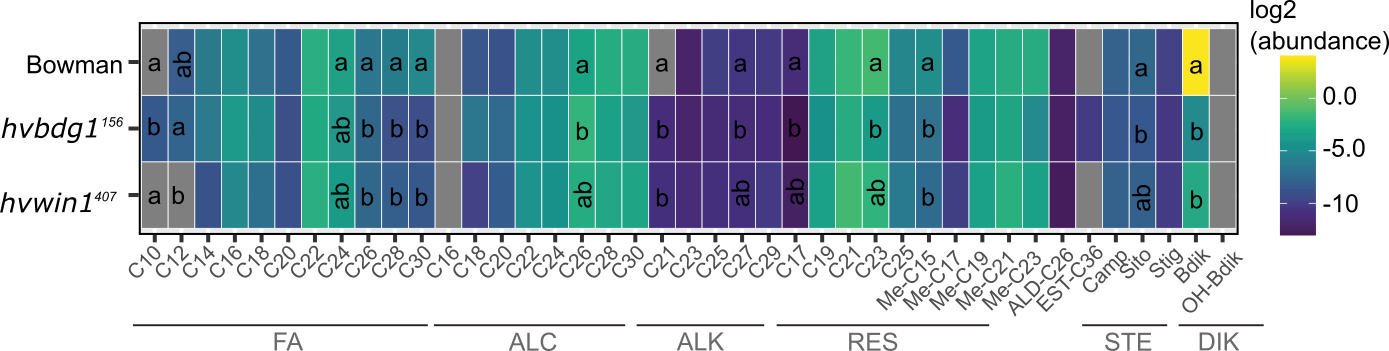


**Fig. S4 *Cer-U* qRT-PCR expression in the mid flag leaf sheath region of *Hordeum vulgare* L. Bowman near-isogenic lines (BW-NILs) *hvbdg^156^* (BW156) and *hvwin1^407^* (BW407), expressed as relative quantity (RQ).** Bars indicate the mean of three biological replicates. Coloured circles show the average of three technical replicates of each independent biological replicate. Flag leaf sheaths were harvested when auricles were 1 to 3 cm above the auricles of the second-to-flag leaf sheaths. (n = 3/ genotype).


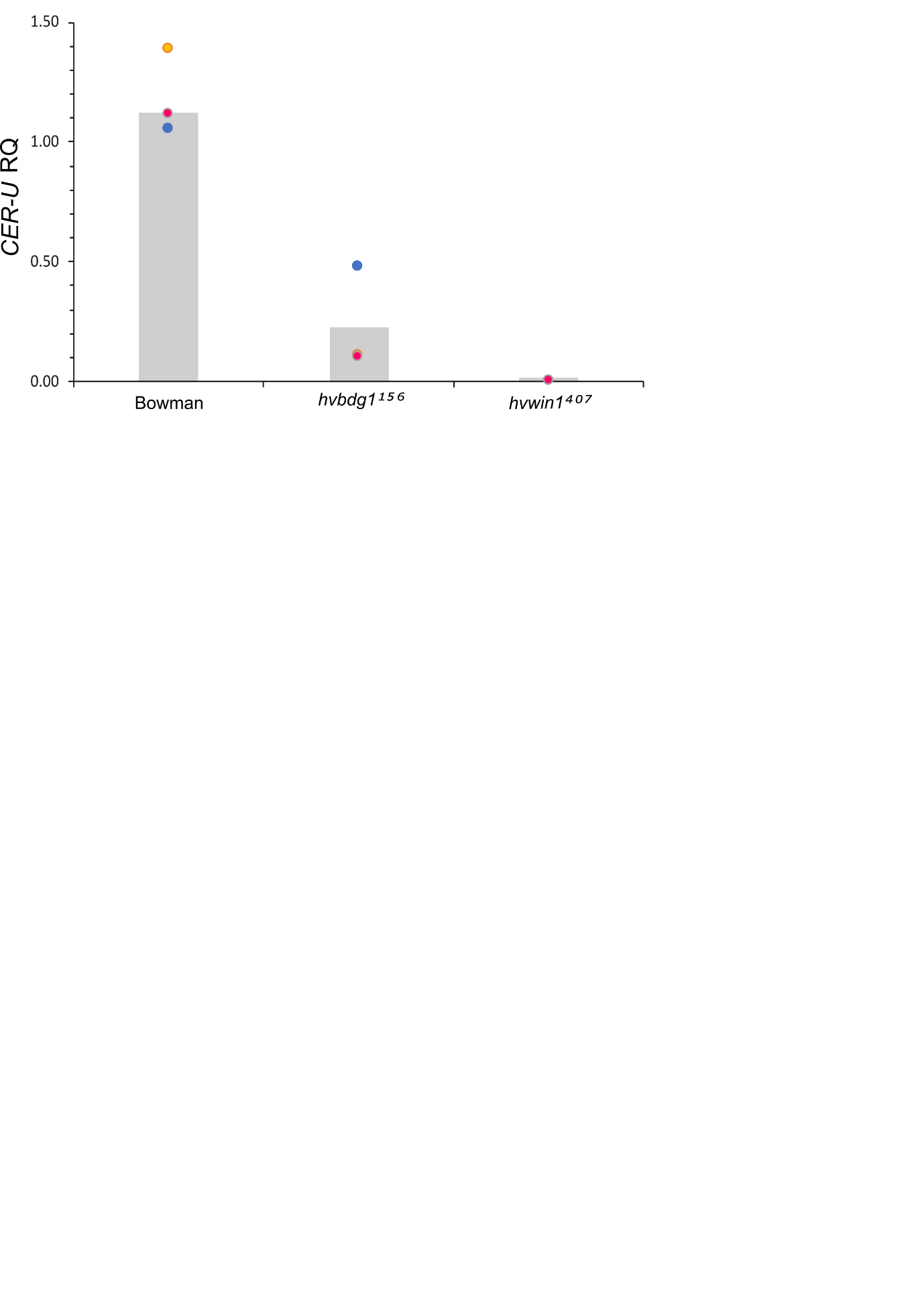


**Fig. S5 Phylogenetic relationship of the BDG family.** (a) Between members of the BODYGUARD family in model angiosperm species. Evolutionary analysis was inferred by using the Maximum Likelihood method and JTT matrix-based model in MEGA X (Jones et al., 1992; Kumar et al., 2018). The tree with the highest log likelihood (-12129.36) is shown. The percentage of trees in which the associated taxa clustered together in 500 bootstrap replications is shown next to the branches. A BDG-like protein from *Physcomitrium patens* was used as an outgroup to root the tree. Green: Cereal specific clade of BDG1 proteins. Blue: BDG4 clade diverged before the emergence of angiosperms. Bold: barley proteins. (b) BDG proteins emerged early in the green plant lineage. Schematic of evolutionary relationships between representative species in the green plant lineage and the number of BDG proteins identified in each species.

**(a)**


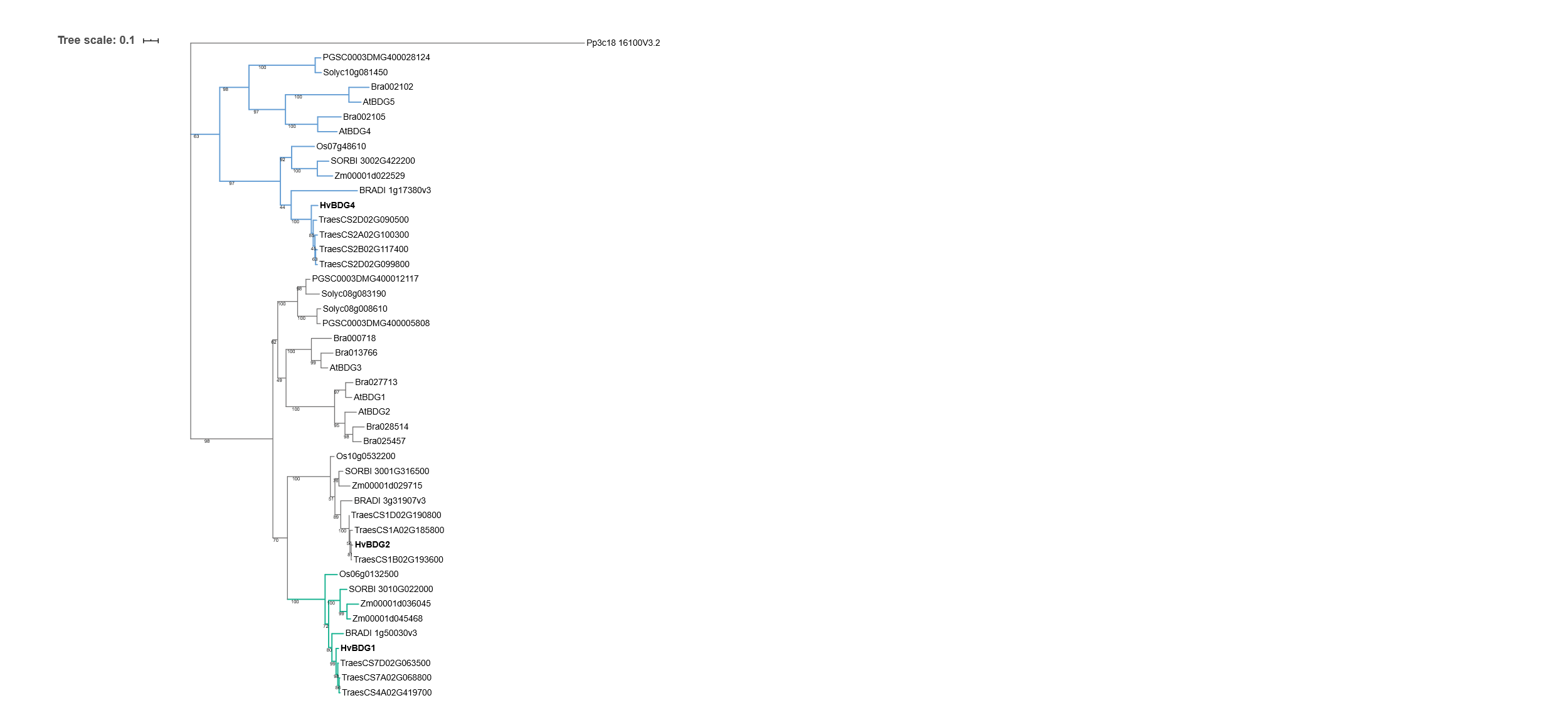

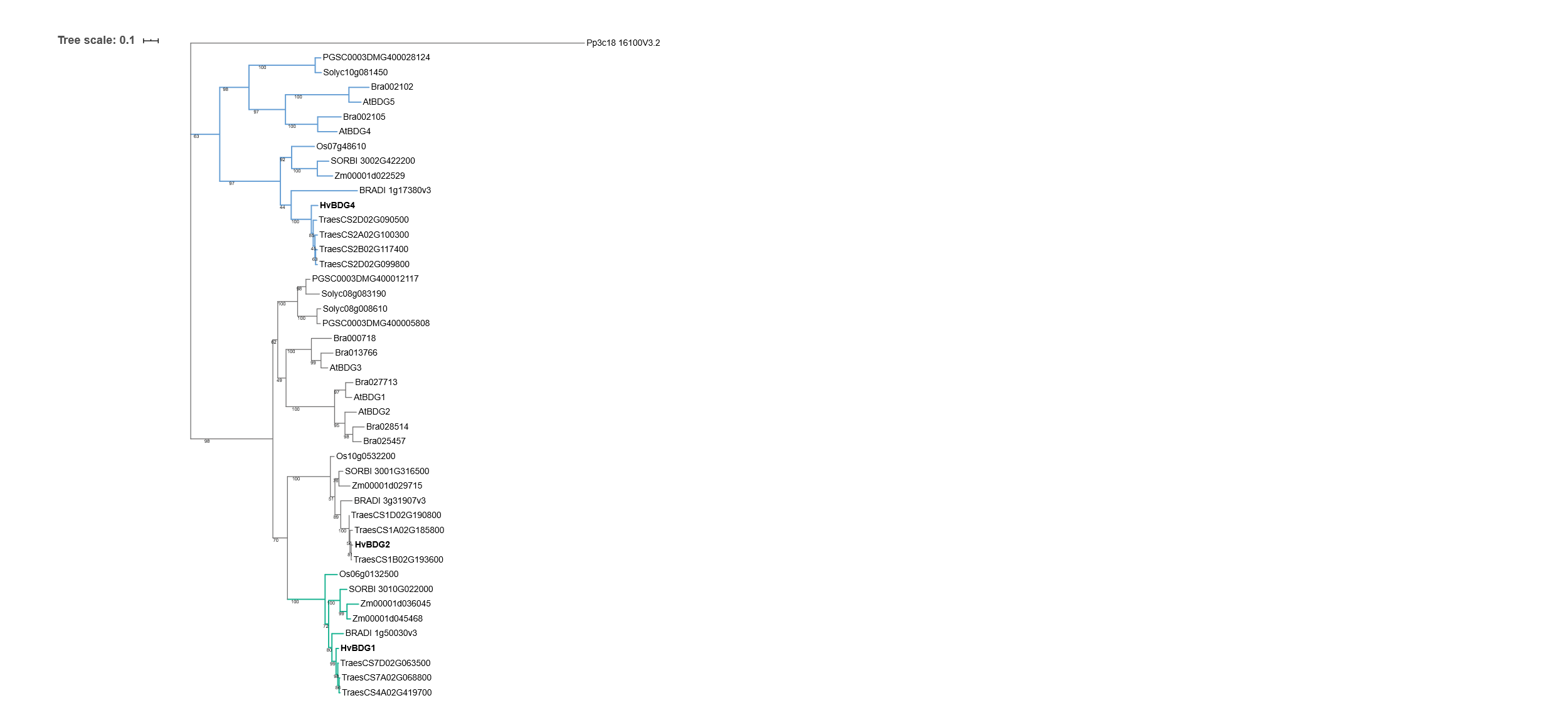


**(b)**


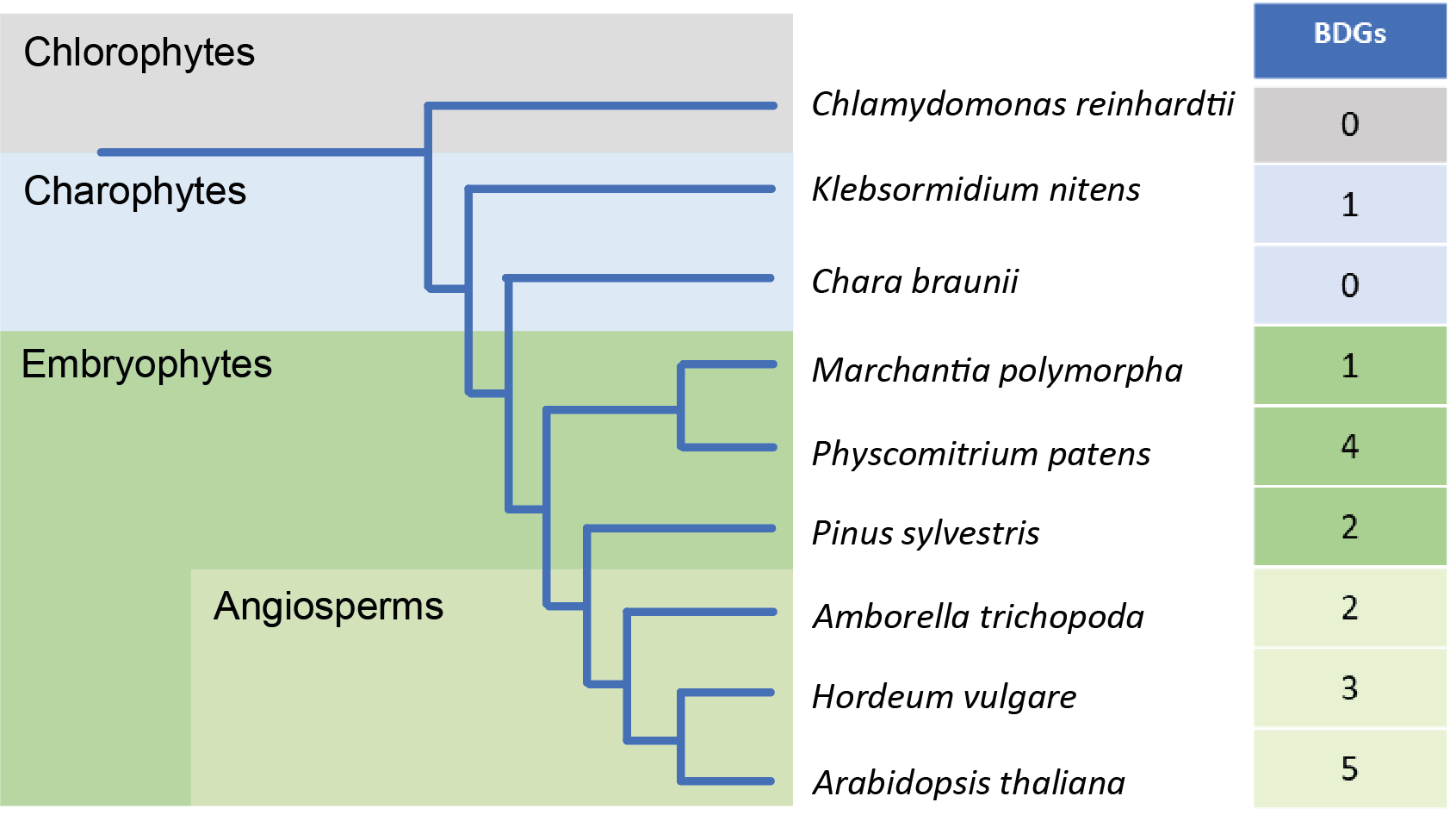


**Fig. S6 Expression profiles of *HvBDG* genes** in *Hordeum vulgare* L. **barley cultivar Morex.** Data extracted from the Barley Expression Database EoRNA (Milne et al., 2021). CAR: caryopsis, EMB: embryo, EPI: epidermis, ETI: etiolated seedling, INF: inflorescence, LEA: leaf, LEM: lemma, LOD: lodicule, NOD: stem, PAL: palea, RAC: rachis, ROO: root, SEN: senescing leaf. Bars represent the mean of three biological replicates. TPM: Transcripts per million.

**
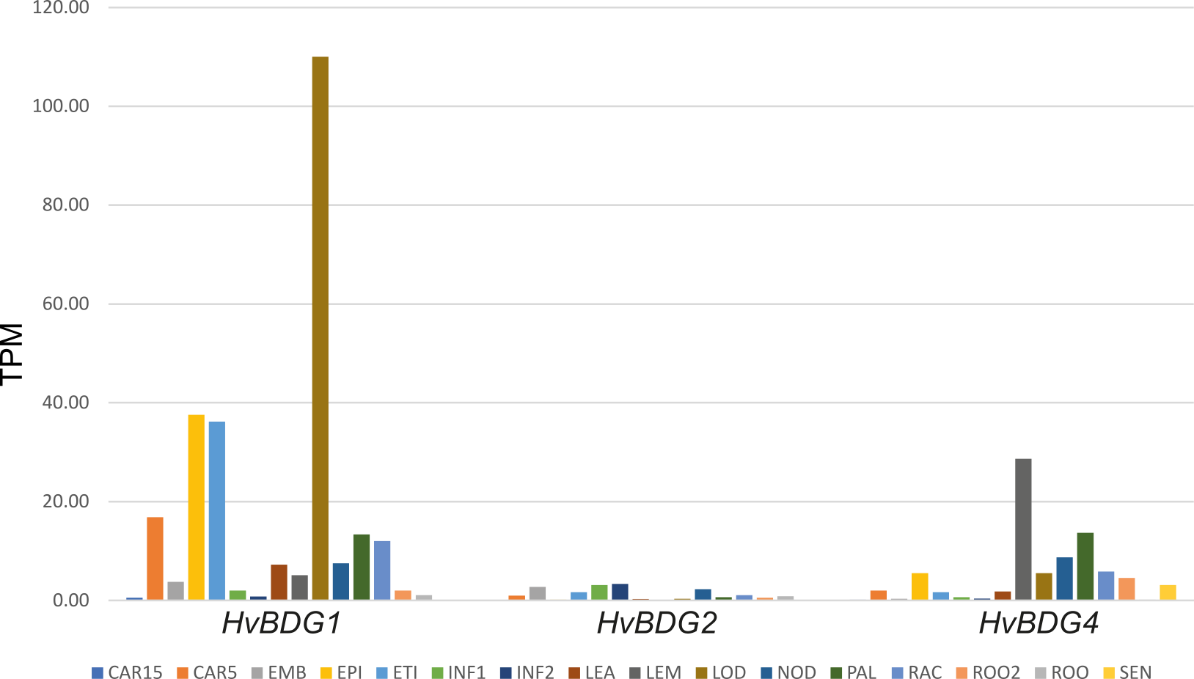
**

**Fig. S7 BDG protein motifs.** Domain alignment and analyses of *Hordeum vulgare* L. HvBODYGUARD1 (HvBDG1) (a) Domains mapped onto the *HvBDG1* gene model. Blue: BDG domain. Green: α/β hydrolase core domain. Red: Histidine 407 (H) to Arginine (R) SNP found in BW156 and BW406. Purple: “Lid” domain. Yellow: Amino acid residues of the catalytic triad (Serine 299, Aspartic acid 448 and Histidine 476). (b) BDG motifs plotted onto the HvBDG1 protein. (c) Alignment of characterised BDG proteins with conserved motifs highlighted above. First 3 sequences in alignment belong to the grass specific BDG1 clade containing the conserved N-terminal sequence (red, motif 1). Blue: BDG domain motifs. Green: known alpha/beta hydrolase domain motifs. Orange: novel alpha/beta hydrolase motifs. Purple: novel motifs in the lid domain. Barley: HvBDG1 (predicted protein corresponding to gene model *HORVU.MOREX.r3.7HG0644300*), HvBDG2 (*HORVU.MOREX.r2.1HG0040690*) and HvBDG4 (*HORVU2Hr1G017080*). Bread wheat: TdBDG1-A1 (*TRITD7Av1G017740*) and TdBDG1-A2 (*TRITD4Av1G244120*). Arabidopsis: AtBDG1 (*At1g64670*), AtBDG2 (*At5g41900*), AtBDG3 (*At4g24140*), AtBDG4 (*At5g17780*), AtBDG5 (*At5g17720*).

**
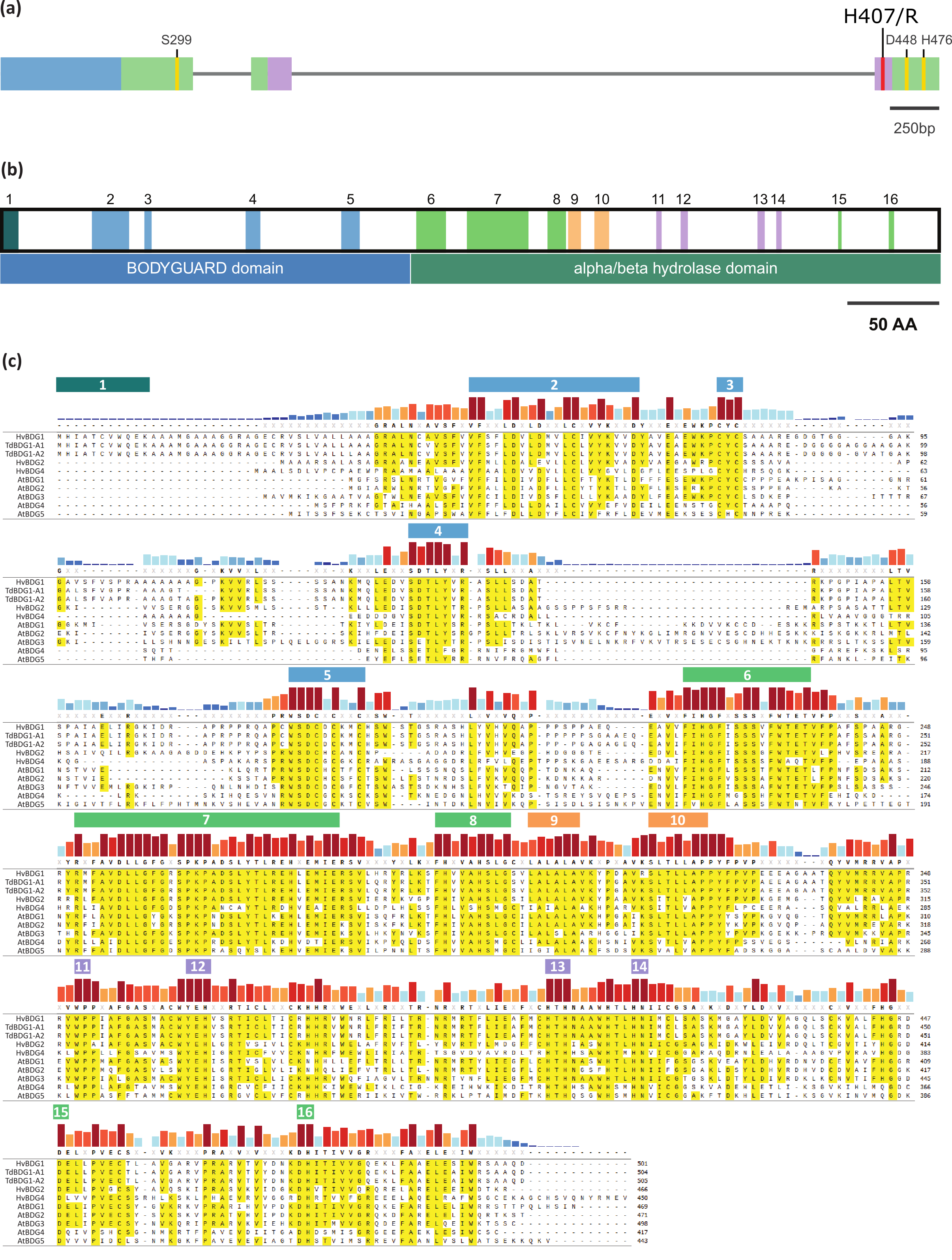
**

**Fig. S8 Protein modelling of HvBDG1.** Modelling and analyses of HvBODYGUARD1 (HvBDG1). (a) HvBDG1 *cv.* Bonus protein model where regions and domains are overlaid with an opaque surface. Model on left coloured by domain: The alpha-beta hydrolase_1 domain (Cdvist ID by hmmer3 and database 30.0, Score: 75.5, region: aa219-347) shown in dark blue within a larger BDG1 domain containing hydrolase in pale blue (NCBI Conserved Domain Database ID, PLN03087, accession: cl30400, region: 42-496, E-value: 0e+00). The catalytic residues S299, D448 and H476 are shown in orange as is the H225, situated in the active site and likely important for stabilising the reaction. Middle panel shows the surface according to Eisenberg's scale of hydrophobicity showing a hydrophobic pocket (red) in which the catalytic residues reside. Right panel presents a qualitative representation of electrostatic potential generated with vacuum electrostatics showing a shallow negatively charged cleft leading to the active site, with a moderately positive charge in the active site pocket. (b) ConSurf Colour-Coded conservation based on MSA of 150 sequences selected using HMMER and UniRef90 with a cutoff of E = 0.0001, a CD-Hit maximum cutoff of 95% and minimum 35% with 10% maximal overlap between homologues, projected on the structural model of Bonus HvBDG1. (c) Ribbon diagrams of HvBDG1 *cv.* Bonus and HvBDG1 H470/R protein models. HvBDG1 *cv.* Bonus model on left shows regions and domains identified by multiple tools. Conserved domain analysis revealed a central Abhydrolase_1 domain (CDvist ID; HMMER3, database v30.0; score: 75.5) spanning residues 219-347, depicted in dark blue. This domain lies within a broader Bodyguard 1 hydrolase domain (NCBI Conserved Domain Database ID: PLN03087, accession: cl30400; residues 42-496; E-value: 0.0), shown in orange. Substantial overlap was observed with additional domain annotations, including the UniProt-assigned AB hydrolase-1 domain (residues 221-407), the PANTHER-assigned hydrolase domain (PTHR43689; residues 141-454), and InterPro entries for alpha/beta hydrolases: the superfamily SSF53474 (IPR029058; residues 162-450) and the CATH-Gene3D integrated region (residues 160-461). Overlapping regions are not highlighted. The image on the right shows the approximate region destabilised by site H407/R mutation (turquoise), corresponding with the “lid” domain, and the region attached that is predicted to structurally rely upon the position of this (purple), corresponding with most of the BDG domain. However, this region includes α-helix 2. We are not confident that this helix interacts with the region in turquoise as we predicted a transmembrane domain within this structure using Phyre2. (d) Protein structural flexibility by coarse-grained protein modelling with CABS-flex 2.0 using ReFOLD-refined Intfold7 structural predictions. Top panel shows the flexibility predictions of the HvBDG1 cv Bonus where histidine aa407 beyond the Nʹ terminus of α-helix 21 is held in place by an interaction internal to α-helix 18 with serine aa368. Bottom panel the same region in the HvBDG1 H407/R mutant, where the arginine residue no longer interacts with S368 leading to no interaction between α-helix 18 and 21 and a change in conformational state in the local areas area. (e) By residue structural flexibility in HvBDG1 *cv.* Bonus and HvBDG1 H407/R. Increased flexibility in the preceding ≈ 150 aa and decrease in flexibility in the immediate area between ≈ 375-400 aa due to apparent compression of the local environment.

**
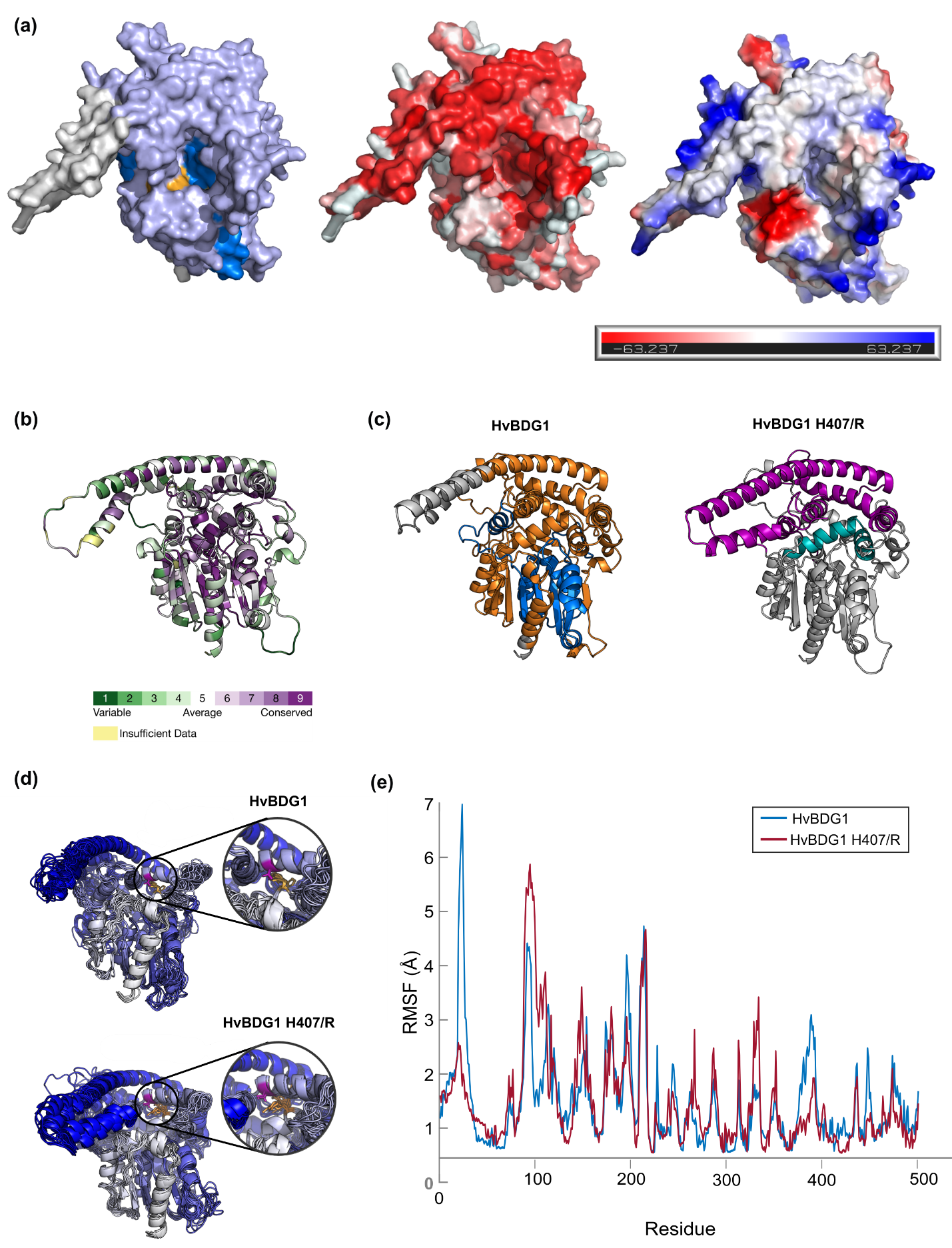
**

**Fig. S9 Localisation patterns of N-terminally and C-terminally tagged HvBDG1 constructs.** Localisation in infiltrated *Nicotiana benthamiana* leaves transiently expressing constructs as indicated. All images were captured in the same session and using the same capture parameters. tHvBDG1 represents a truncated version which lacks the 14 amino acid grass-specific N-terminus. Arrows indicate mobile bodies. Reticulate patterns were also observed for each construct but low signal made it challenging to capture this clearly above background noise. Scale bars: 5 µm.


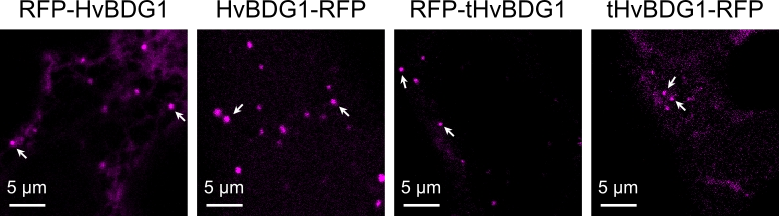


**Fig. S10 Durum wheat stem and sheath phenotypes in *TdBDG1*and *TdWIN1*mutants.** Examples of wax bloom phenotype observed in durum wheat (*Triticum turgidum ssp. durum*) leaf sheath (top row) and stems (bottom row) from F_2_ individuals carrying different wild-type and mutant alleles at homoeologues of (a,b) *TdBDG1*and (c,d) *TdWIN1*. Two combinations of mutants are shown for both *TdBDG1*and *TdWIN1*. For each combination, allelic state at the A- and B- sub-genome homoeologue is indicated via uppercase (wild-type) or lowercase (mutant) letters, such that homozygous wild-type (AA or BB), homozygous mutant (aa or bb), and combinations of homozygous and wild type (e.g., AABb) alleles are indicated.


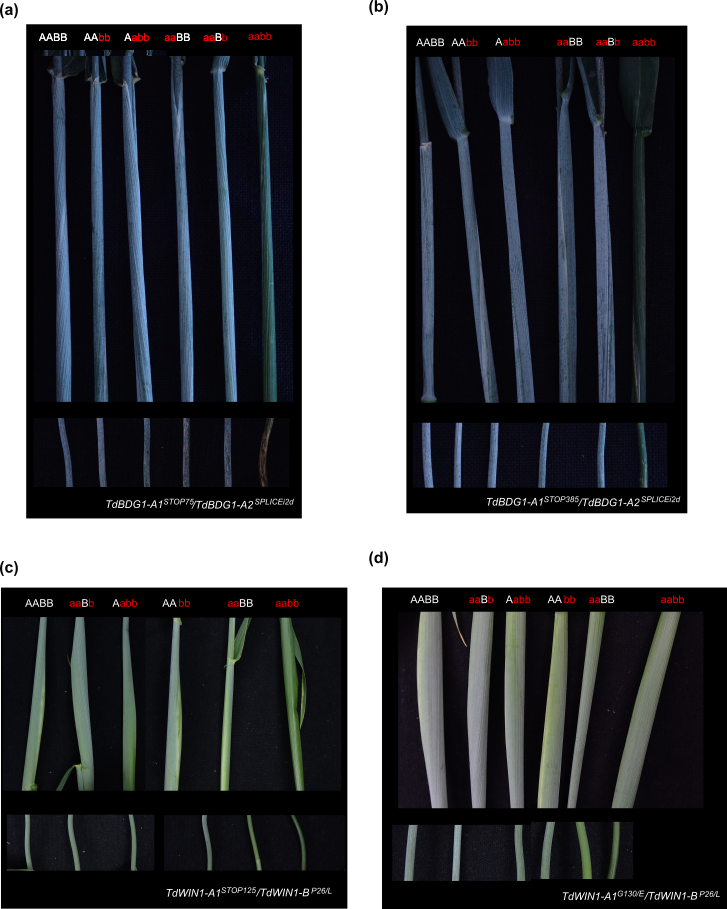


**Fig. S11 Heat maps showing surface lipid chain lengths extracted from leaf sheaths from** (*Triticum turgidum ssp. durum*) durum wheat**.** Asterisks (*TdWIN1*) and letters (*TdBDG1*) indicate significant differences within genotypes (P < 0.05; Tukey’s HSD multiple comparison following one-way ANOVA). (n = 4/ genotype). Scale bar indicates compound relative abundance/mg sample fresh weight on a log_2_ scale. FA, fatty acids; ALC, alcohols; ALK, alkanes; RES, resorcinols; ALD, aldehydes; STE, sterols; EST, esters; DIK, diketones.


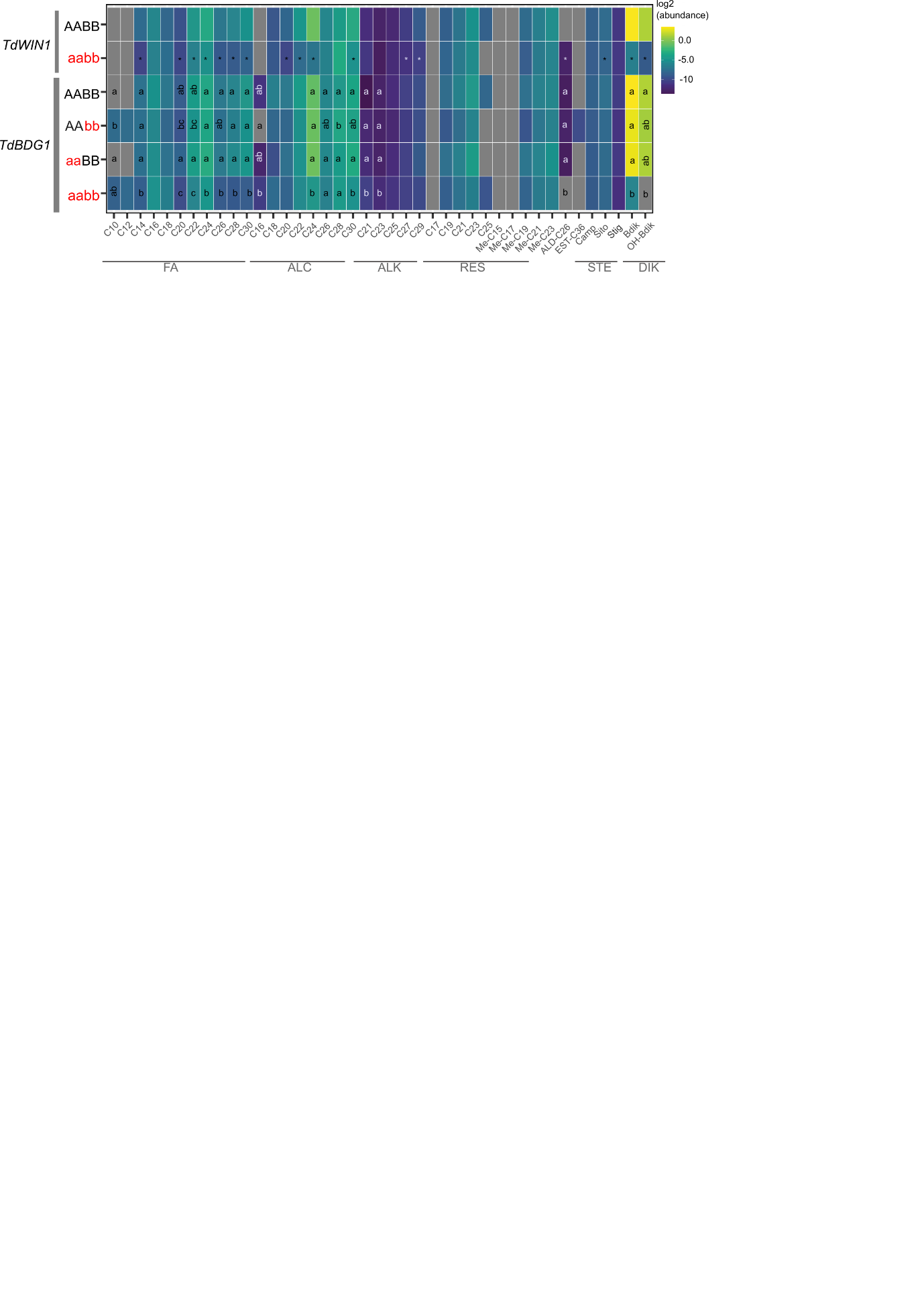


**Fig. S12 Cuticular ridges on barley caryopses**. Scanning electron microscopy (SEM) of 7 days post anthesis (DPA) caryopses (hulls removed) pericarp surfaces in *Hordeum vulgare* L. (a) Bowman, *nud^638^* and *hvbdg1^156^*. (b) Bowman and *hvwin1^407^* compared in a separate experiment to (a). Scale bars, 10 μm. White arrowheads show longitudinal cuticular ridges, black arrowheads perpendicular striations and black arrows perpendicular nanoridges. Striations were larger than nanoridges.


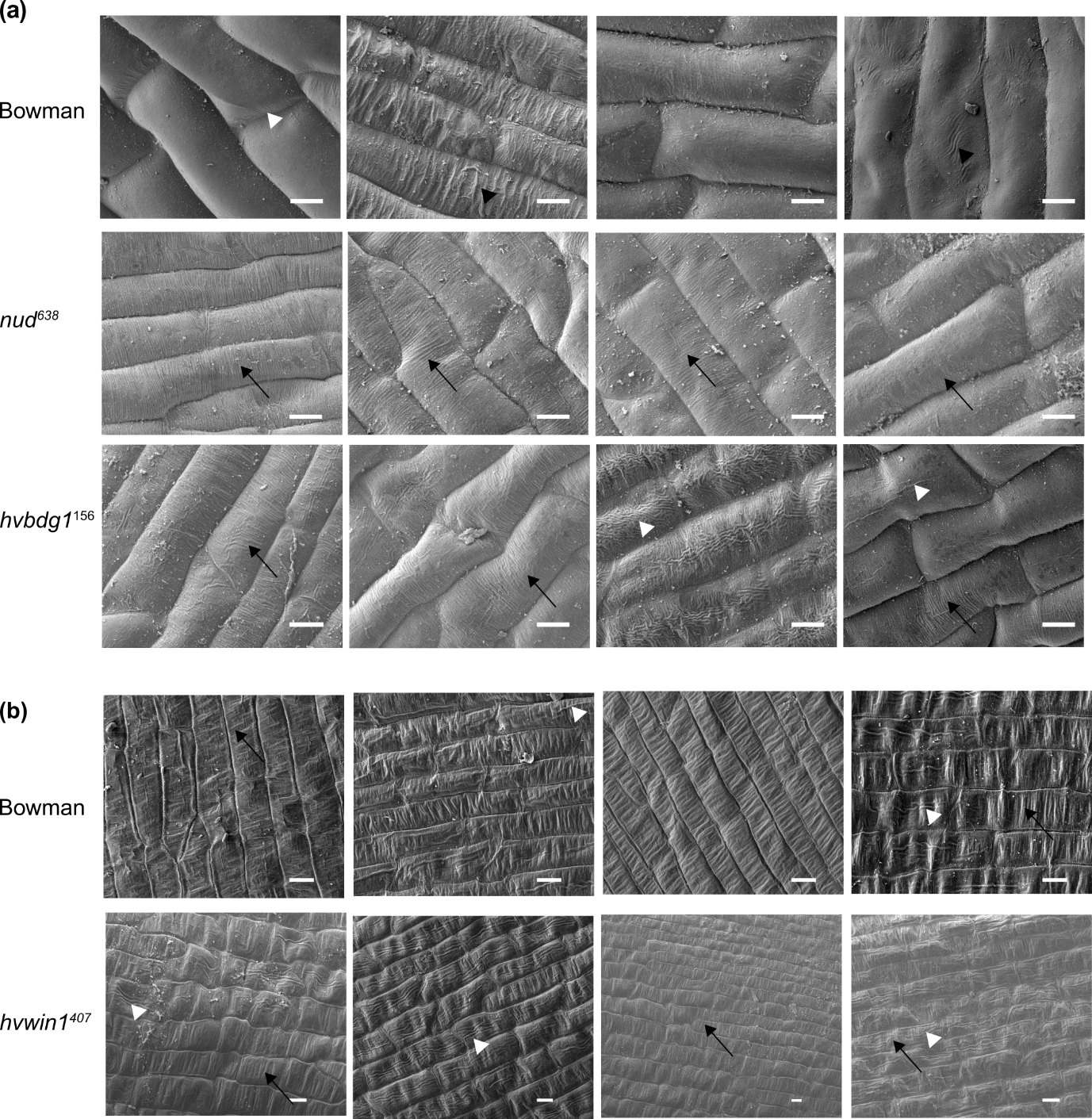


**Fig. S13 Bowman caryopsis cuticle 7 days post anthesis (DPA)**. Transmission electron micrographs of barley in *Hordeum vulgare* L. pericarps of (a) Bowman. (b) *nud^638^*. Scale bars, 500 nm. Images are representative of at least three biological replicates.

*
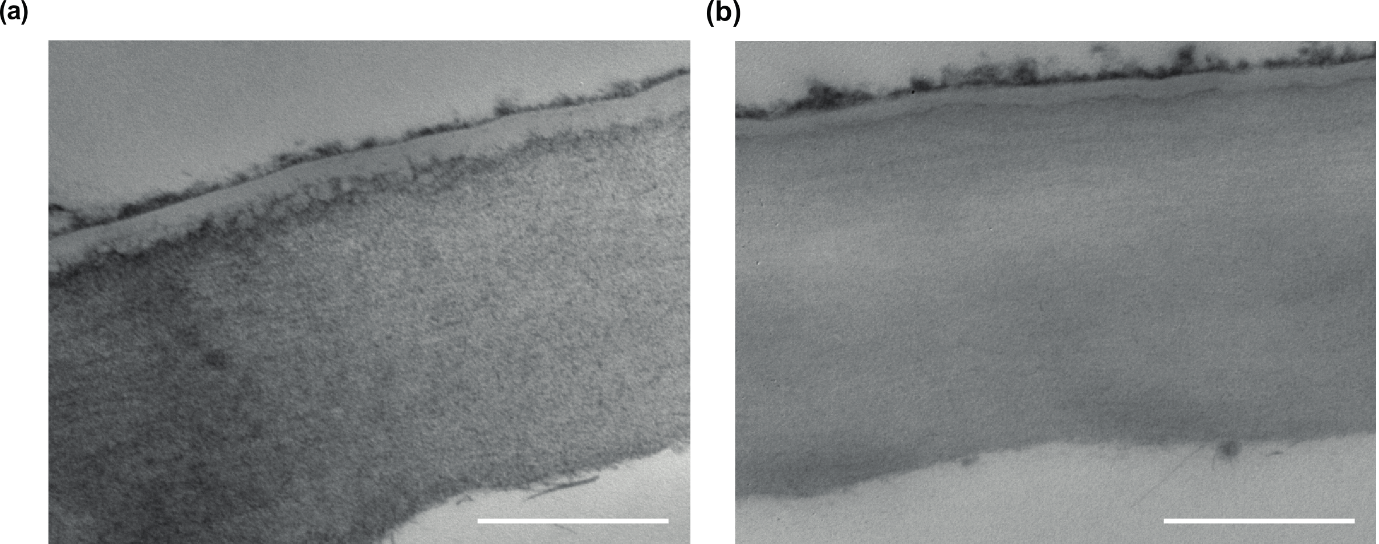
*

**Fig. S14 Cutin monomers from barley hull and caryopses during adhesion**. Bar graphs showing (a) Cutin monomers extracted from hulls dissected from 11 days post anthesis (DPA) hulls from in *Hordeum vulgare* L. Bowman, *nud^638,^ hvbdg1^156^* and *hvwin1^407^*; (b) Cutin monomers extracted from 5 DPA and 11 DPA caryopses of Bowman, *nud^638,^ hvbdg1^156^* and *hvwin1^407^*. Three biological replicates, except for Bowman 11 DPA hulls in the experiment with *hvwin1^407^* (right bottom panel) and *hvwin1^407^* 5 DPA caryopses where replicates were two. Letters indicate significant differences within genotypes (P < 0.05; Tukey’s HSD multiple comparison following one-way ANOVA). Scale bar indicates compound relative abundance/caryopsis on a log_2_ scale. FER, ferulic acid; COU, coumaric acid; ω-OH C16, 16-hydroxyhexadecanoic acid; ω-OH C18, 18-hydroxyoctadecanoic acid; ω-OH C18:1, 18-hydroxyoctadecenoic acid; ω-OH 9,10 epoxy C18, 9,10-epoxy-18-hydroxyoctadecanoic acid.


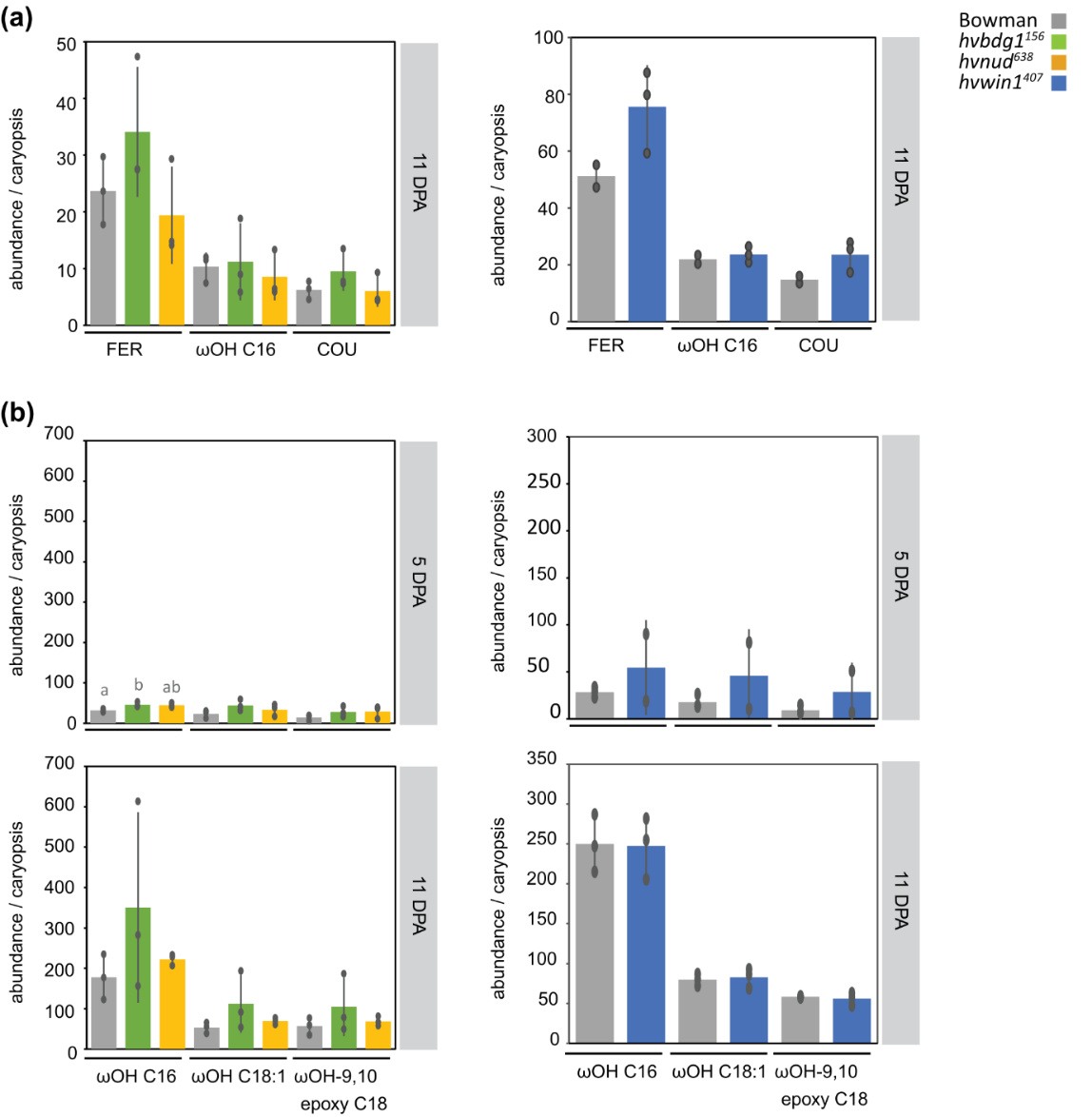


**Fig. S15 Quantitative wax load of barley hulls.** (a,c) Bar graphs showing soluble surface lipid classes and (b,d) heat maps showing chain lengths extracted from in *Hordeum vulgare* L. hulls dissected from 5 and 11 days post anthesis (DPA) caryopses (n = 3/ genotype). Letters (Bowman, *hvbdg1^156^*,*nud^638^*) and asterisks (Bowman and *hvwin1^407^*) indicate significant differences within genotypes within one stage (P < 0.05; P < 0.001 = ***; Tukey’s HSD multiple comparison following one-way ANOVA).  Y axes indicate compound relative abundance/caryopsis and scale bar indicates compound relative abundance/caryopsis on a log_2_ scale. TOT, total extract; DIK, diketones; ALK, alkanes; FA, fatty acids; ALC, alcohols; STE, sterols.


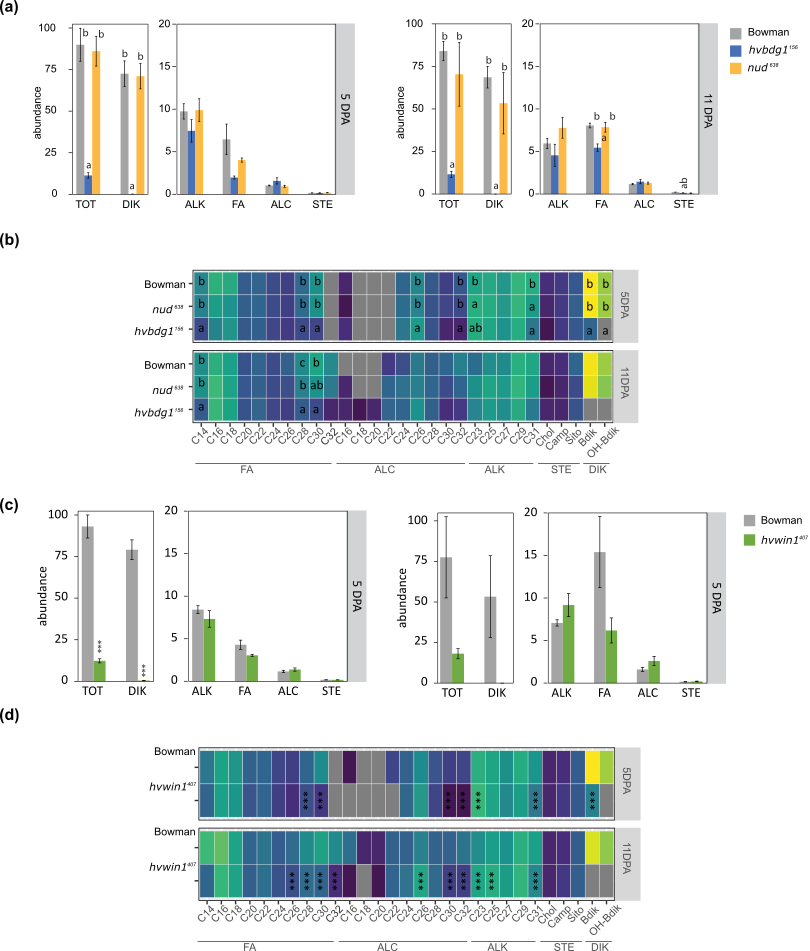


**Fig. S16 Quantitative wax load of barley leaf sheaths.** (a) Bar graphs showing soluble surface lipid classes and (b) heat maps showing chain lengths extracted from in *Hordeum vulgare* L. Bowman, single mutants (*hvbdg1^156^*,*hvwin1^407^*, *nud^638^*), and double mutants (*nud^638^ hvwin1^407^*, *hvbdg1^156^* *nud^638^* and *hvbdg1^156^* *hvwin1^407^*) leaf sheaths (n = 4/ genotype). Letters indicate significant differences within genotypes (P < 0.05; Tukey’s HSD multiple comparison following one-way ANOVA).  Y axes indicate compound relative abundance/mg sample fresh weight and scale bar indicates compound relative abundance/mg sample fresh weight on a log_2_ scale. TOT, total extract; DIK, diketones; RES, resorcinols; ALC, alcohols; FA, fatty acids; STE, sterols; ALK, alkanes; EST, esters; ALD, aldehydes.


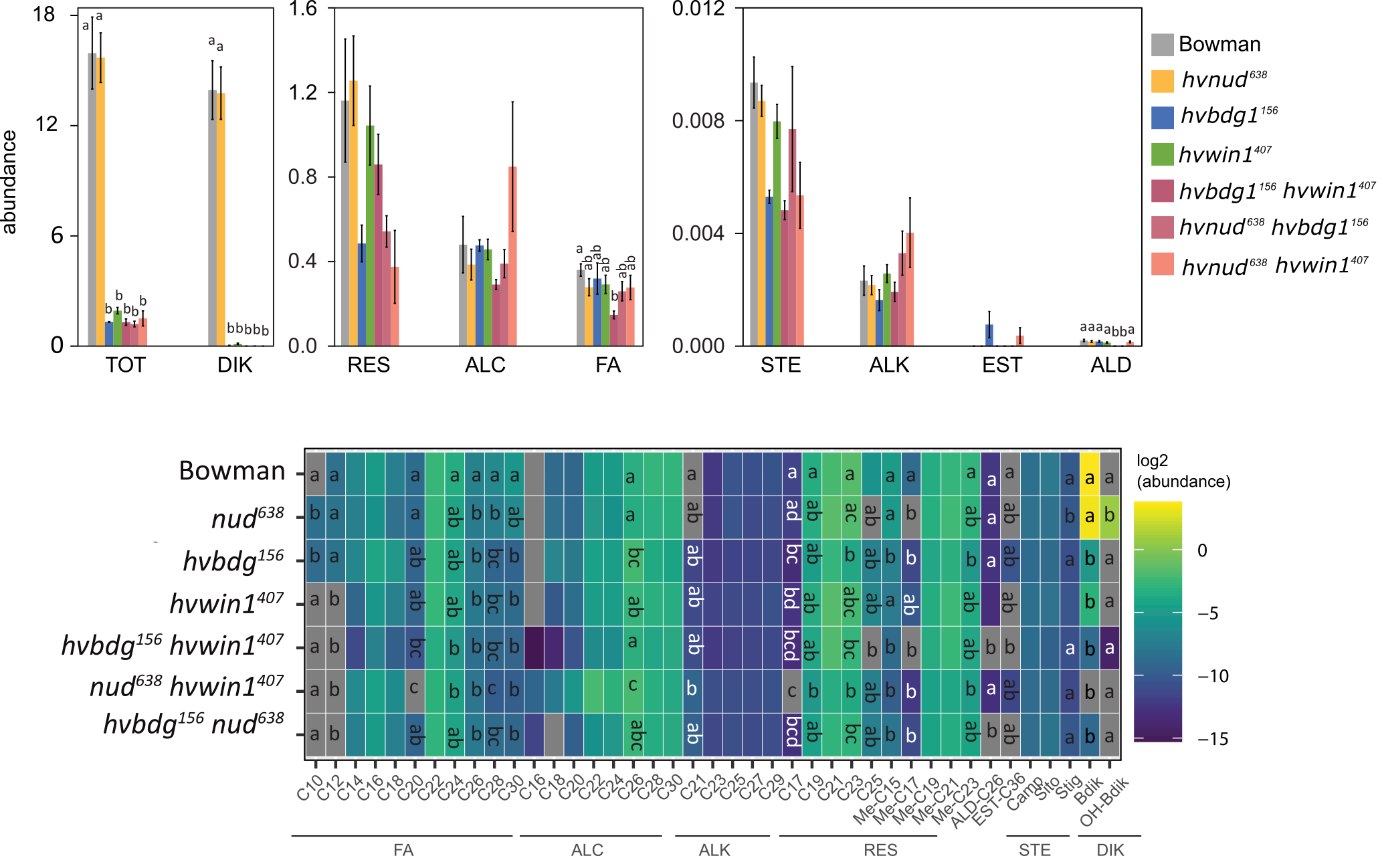


**Fig. S17 Chlorophyll leaching in leaf blades of wild-type Bowman, *hvbdg1^156^*, *hvwin1^407^*, *nud^638^*, and double mutants**. Chlorophyll leaching of detached in *Hordeum vulgare* L. leaf blades into 80% (v/v) ethanol. Bars show the average with standard error (n = 4/genotype). Letters refer to significant differences using an analysis of variance (ANOVA, p < 0.0001) and the post-hoc Tukey’s HSD test using Area Under the Curve (AUC). (n = 4/ genotype).


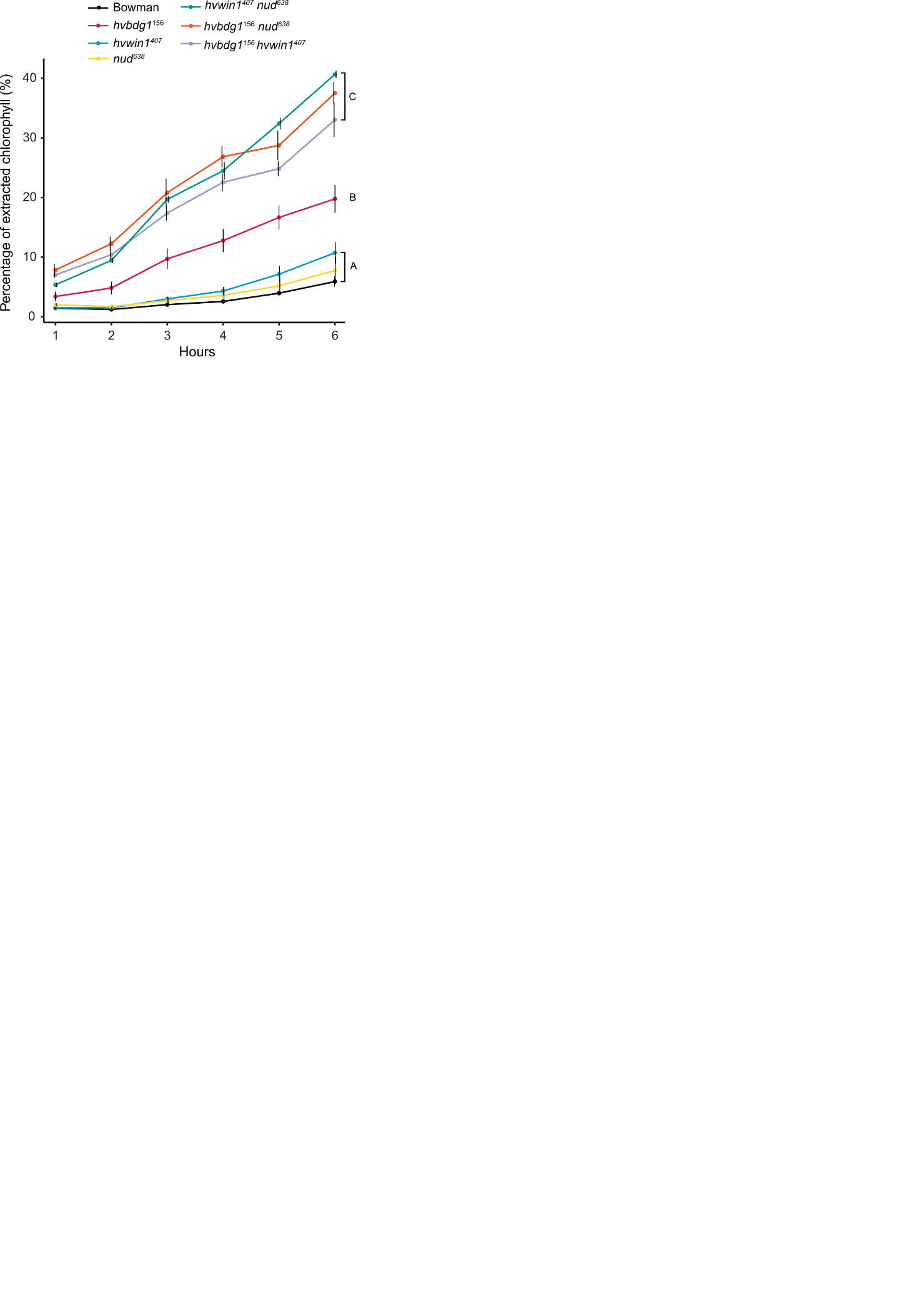


**Fig. S18 NUD and HvWIN1** **expression measured by qRT-PCR in developing second leaf blades.** Results from barley Hordeum vulgare L. cv. Bowman, hvnud^638^ and hvwin1^407^, expressed as relative quantity (RQ). (a) NUDUM (NUD) expression in the leaf base. (b) HvWIN1 (HvWIN1) expression in the leaf base. (c) NUD expression in the mid-leaf. (d) HvWIN1 expression in the mid-leaf. Bars indicate mean expression relative to Bowman. Black circles represent independent biological replicates, each the average of three technical repeats. (n = 3/ genotype).

**
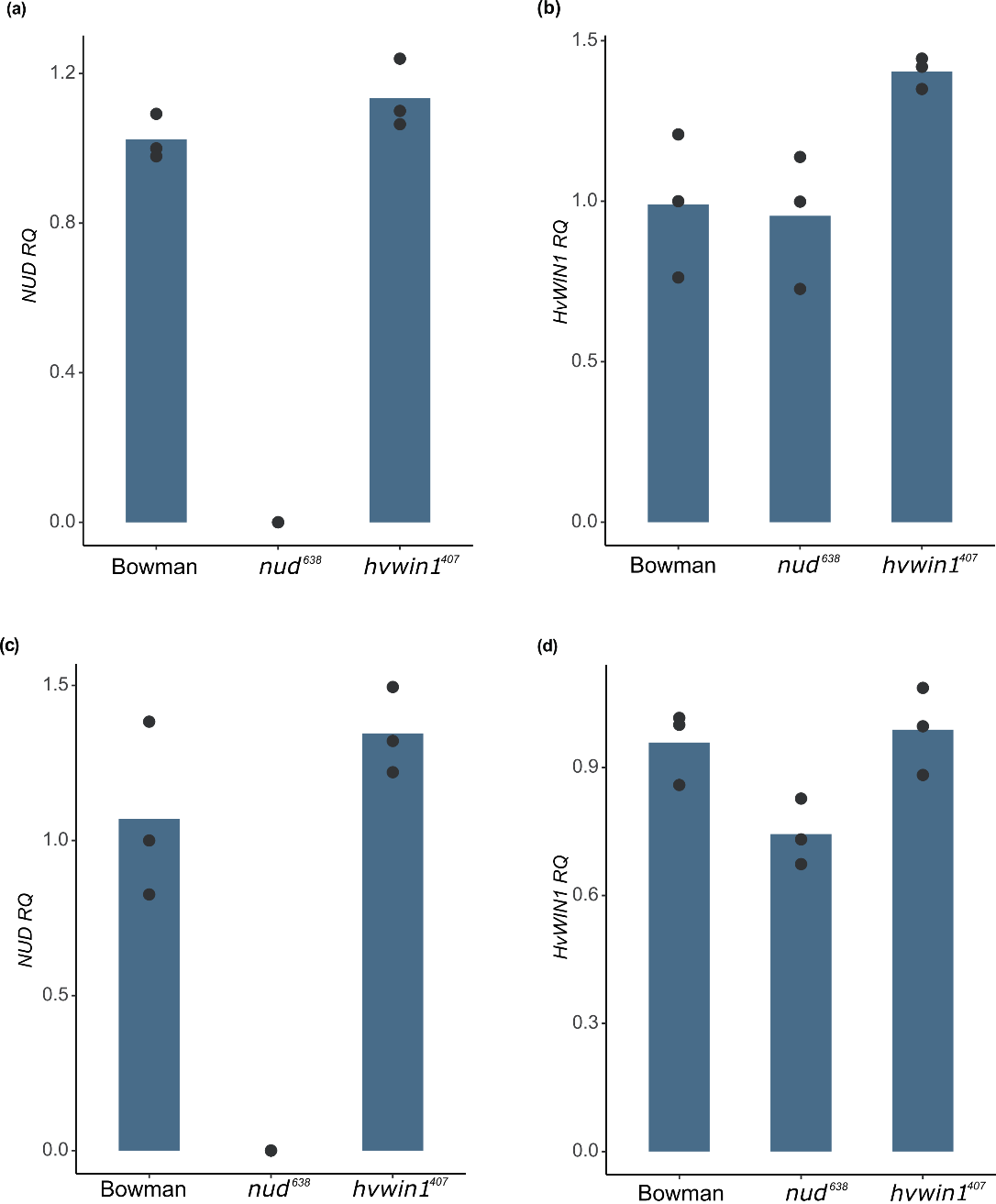
**

**Fig. S19 *HvBDG1* expression measured by qRT-PCR in mid-leaf blade sections of barley.** Results from *Hordeum vulgare* L. *cv.* Bowman, *hvnud^638^* and *hvwin1^407^*, expressed as relative quantity (RQ). Bars indicate mean expression relative to Bowman. Black circles represent independent biological replicates, each the average of three technical repeats. (n = 3/ genotype).


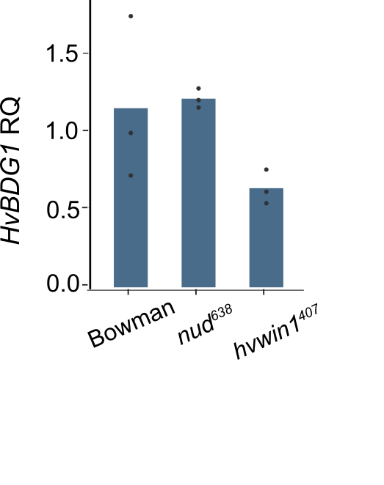


**Fig. S20 Regulatory relationships between barley SHINE transcription factors, HvBDG1 and surface feature**. NUD and HvWIN1 are essential regulators for two late stage and agronomically relevant cuticular elaborations in barely (*Hordeum vulgare* L.), the wax bloom and hull adhesion, respectively, but retain functional, independent overlap in leaf cuticle integrity and hull adhesion. Each of these functions involve upregulation of *HvBDG1* and other direct or indirect targets, shared and distinct, depending on the stage and tissue.


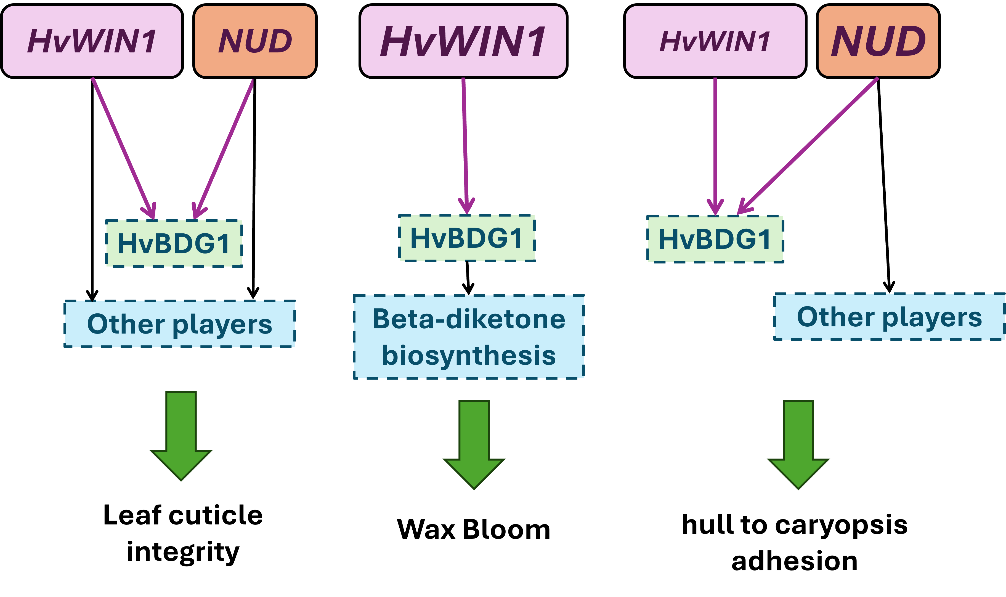


**Note S1** **Detailed lipid compound analyses**. Derivatised soluble surface lipids from flag leaf sheaths of barley (*Hordeum vulgare* L.) were analysed using an Agilent 7890B GC System coupled to a LECO Pegasus BT4D time-of-flight mass spectrometer and equipped with a PAL3 autosampler (LECO UK). Separation was achieved on a DB-5MS capillary column (Agilent Technologies, UK) using helium as the carrier gas at a constant flow rate of 1.5 mL/min. A 1 µL aliquot was injected in split mode (1:40) at an inlet temperature of 250 °C. The oven temperature was held at 100 °C for 2.1 min, ramped at 25 °C min⁻¹ to 330 °C, and held for 5 min. The transfer line and ion source temperatures were set at 325 °C and 230 °C, respectively. Mass spectra were acquired in electron ionisation mode (70 eV) over an m/z range of 35–900 at 10 spectra s⁻¹. Compounds were identified by comparison with the NIST Mass Spectral Library version 2.4 and with literature (Adamski et al., 2013; Campoli et al., 2024).

**Note S2** **Detailed validation and characterisation of HvBDG1 protein models.**  We generated protein models for barely (*Hordeum vulgare* L.) Bonus HvBODYGUARD1 (HvBDG1) using ReFOLD-refined IntFOLD-TS structural prediction with AlphaFold2 and trRosetta2. We observed that ReFOLD refinement improved the prediction by 2.1% resulting in a DeepUMQA-X model quality assessment with TM score of 0.865 and Global- Inter-residue distance deviation (lDDT) of 0.718 (Fig 1). Global lDDT is brought down by an ≈ 76-128aa Nʹ terminal region consisting of unstructured glycine-rich linkers, two, short alanine-rich α-helices and a serine-rich region that may provide scaffolding for molecular recognition and adopt a stable structure upon binding a target protein/substrate or environmental changes.
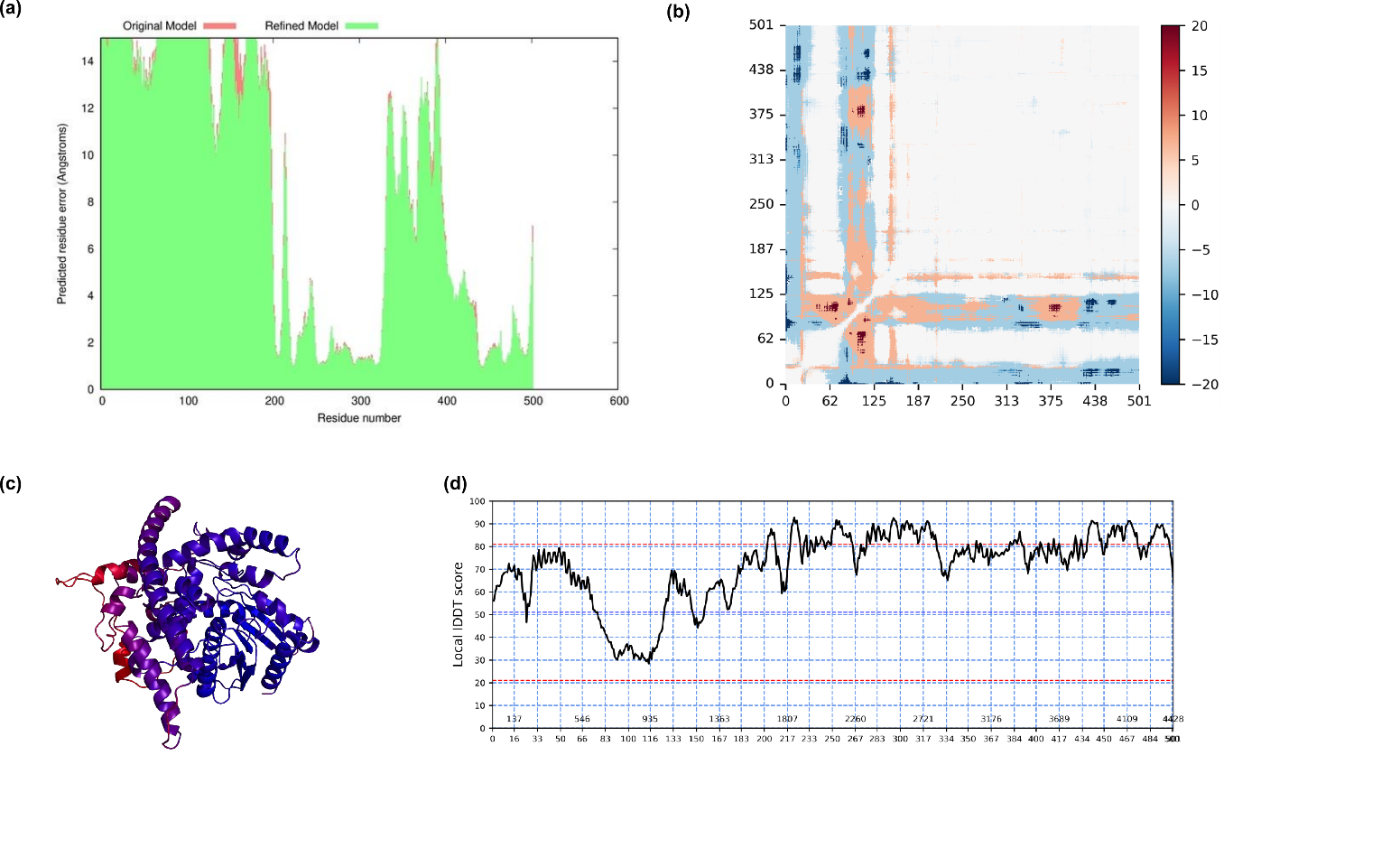
 **Figure 1. Model quality assessments.** (a) Predicted residue error (Å), for the original (orange) and refined (green) models. (b) Inter-residue distance deviation (IDDT) from this assessment. (c) the lDDT mapped onto the model prediction using a low-high/red-blue scale and D shows the per-residue atomic lDDT. To assess stereochemical quality, we conducted Ramachandran plot analysis based on a reference set of 118 high-resolution structures (resolution ≤ 2.0 Å, R-factor ≤ 20%) (Fig. 2). The analysis revealed that 91.9% of residues (406 amino acids) were located within the most favoured regions (A, B, L), while 7.9% (35 residues) occupied additionally allowed regions (a, b, l, p). No residues were found in generously allowed regions (~a, ~b, ~l, ~p), and only one residue (0.2%) was located in a disallowed region. This outlier corresponds to S299, a catalytically important residue, suggesting that its strained backbone conformation may facilitate stabilization of the reaction intermediate. Additional residues with unfavourable Ramachandran Z-scores (< 3.00) include I18, L265, S122, V116, V284, and V463, potentially reflecting local conformational flexibility or functional constraints.
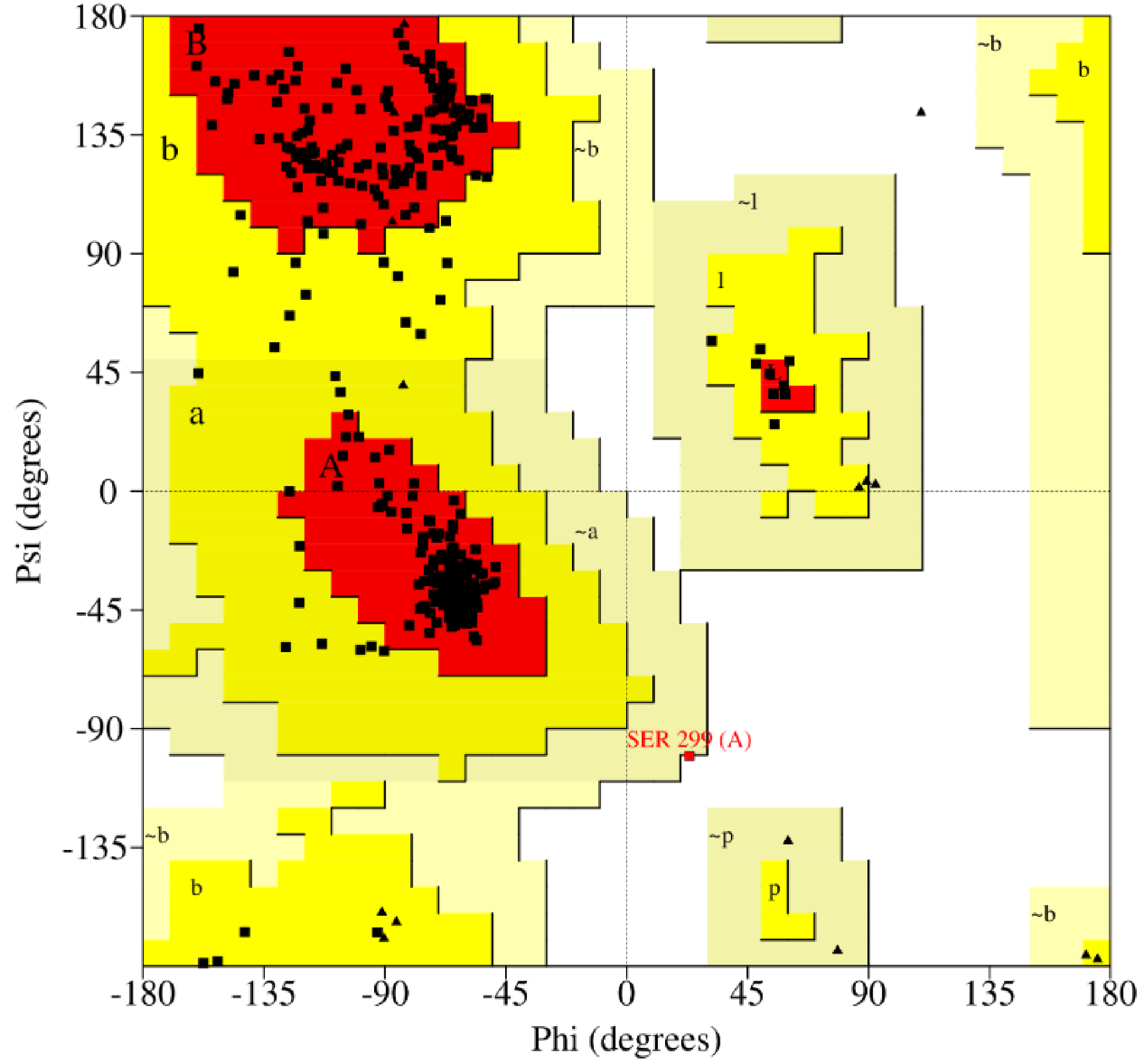
 **Figure 2: Ramachandran Plot Analysis of Bonus HvBDG1.** Regions are coloured based on most favoured (red), additional allowed (yellow), generously allowed (light yellow) and disallowed (white). The catalytically active residue S299 is highlighted in red. We next generated protein models for the mutant HvBDG1^156^ H407/R protein, again using ReFOLD-refined IntFOLD-TS structural prediction utilising AlphaFold2 and trRosetta2. In this case, ReFOLD refinement improved the prediction by 2.0% resulting in a DeepUMQA-X model quality assessment with TM-score of 0.840 and Global-lDDT of 0.667 (Fig 3). Global lDDT was brought down in the same manner as the wild-type Bonus prediction by an ≈ 76- 128aa Nʹ terminal region.
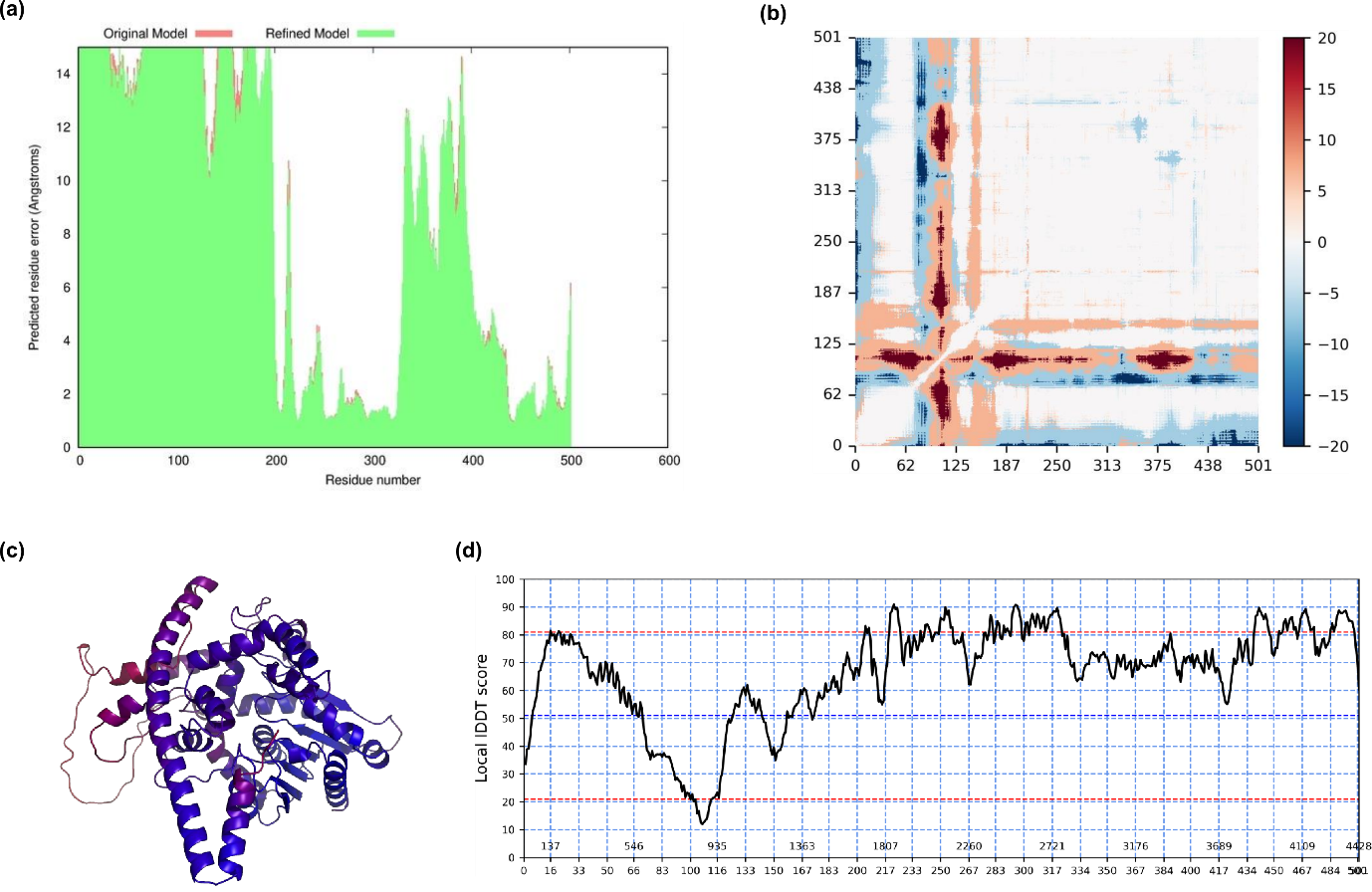
**Figure 3. Model quality assessments of HvBDG1^156^ H407/R.** Models of barely (*Hordeum vulgare* L.) HvBODYGUARD1 (HvBDG1). (a) Predicted residue error (Å), for the original (orange) and refined (green) models. (b) Inter-residue distance deviation (IDDT) from this assessment. (c) the lDDT mapped onto the model prediction using a low-high / red-blue scale and D shows the per-residue atomic lDDT.

**Note S3** **Identification of durum wheat BDG1.** Using barely (*Hordeum vulgare* L.) *BODYGUARD1* coding regions (CDS) from gene model transcript *HORVU.MOREX.r3.7HG0644300.1* as a query for BLASTn analysis of the durum wheat (*Triticum turgidum ssp. durum*) *cv*. Svevo reference genome (assembly Svevo.v1; Maccaferri et al. 2018) using Ensembl Plants (Harrison et al., 2024), identified two genomic regions with e-values <e-^27^, corresponding to the positions of two gene models: *TRITD7Av1G017740* (termed here *TdBDG1-A1*) on the short arm of chromosome 7A at 32.1 Mb (longest High-scoring Sequence Pair: length = 331, 2.5e-^161^) and *TRITD4Av1G244120* (*TdBDG1-A2*) on the long arm of chromosome 4A at 685.5 Mb (longest High-scoring Sequence Pair: length = 331, 2.5e^-161^). Equivalent BLASTn search of the bread wheat *cv*. Chinese Spring genome (assembly RefSeq v1.0; IWGSC, 2018) identified three genomic regions with e-values <e-^27^, corresponding to the positions of three gene models: *TraesCS7A02G068800* on the short arm of chromosome 7A at 34.9 Mb (longest High-scoring Sequence Pair: length = 331, 3.4e^-161^), *TraesCS4A02G419700* on the long arm of chromosome 4A at 690.5 Mb (longest High-scoring Sequence Pair: length = 331, 3.4e^-161^), and *TraesCS7D02G063500* on the short arm of chromosome 7D at 34.5 Mb (longest High-scoring Sequence Pair: length = 331, 5.7e^-166^). The translocated bread wheat 7B chromosomal segment known to be homoeologous to bread wheat chromosomal regions spanning 0-75 Mb on 7A and 0-60 Mb on 7D (Zhou et al., 2020) span the locations of *TaBDG1-A* (7A at 35.9 Mb) and *TaBDG1-D* (7D at 34.351 Mb) are found, respectively, confirming the reciprocal chromosomal translocation T(4AL;7BS)1 present hexaploid wheat (Zhou et al., 2020) and durum wheat (Dvorak et al., 2018) resulted in relocation of the ancestral 7B homoeologue to the long arm of chromosome 4A.

**Note S4** **Differentially expressed genes in barley** (*Hordeum vulgare* L.) *nud^638^* and *hvwin1^407^* **mutants compared to Bowman.** Consistent with roles in grain cuticle modification, we detected genes associated with wax and cutin biosynthesis, lipid transport and cell wall remodelling commonly misregulated in both *nud^638^* and *hvwin1^407^*. Downregulated genes included *CYP86A4* (*HORVU.MOREX.r3.2HG0188260*), encoding a cytochrome P450 hydroxylase required for cutin biosynthesis (Li-Beisson et al., 2009), *CUTIN SYNTHASE2* (*CUS2*; *HORVU.MOREX.r3.6HG0629070*) required for cutin polymerisation and nanoridge maintenance in Arabidopsis and tomato (Yeats et al., 2012; Hong et al., 2017; Sagado et al., 2020), *CUTICLE DESTRUCTING FACTOR CDEF1* (*HORVU.MOREX.r3.3HG0288710*), an esterase shown to have cutinase activity in Arabidopsis (Takahashi et al., 2010), and GDSL *occluded stomatal pore 1* (*OSP1*; *HORVU.MOREX.r3.5HG0468830*) required for wax biosynthesis (Tang et al., 2020). Downregulated DEGs specific to *nud^638^* at either stage (Fig 6f) included cuticular machinery genes encoding β-ketoacyl-CoA synthases (KCSs) catalysing the first step in fatty acyl elongation: HvKCS1 (*HORVU.MOREX.r3.4HG0392320*) whose defective alleles underlie *cer-zh* (Li et al., 2018), homologues of *KCS5* (*HORVU.MOREX.r3.6HG0612530*) and *KCS6* (*HORVU.MOREX.r3.7HG0670360*), as well as the barley *β-ketoacyl-CoA reductase 1* homolog (*KCR1*; *HORVU.MOREX.r3.7HG0653780*), consistent with fewer fatty acids in *nud^638^*. We also detected downregulation of *HvABCG31* (*HORVU.MOREX.r3.3HG0240110*), encoding a putative transporter responsible for the reduced cutin barley mutant *eibi1*, and the gene *HvCER3.2* (*HORVU.MOREX.r3.7HG0741770*; Chen et al., 2011), whose Arabidopsis homologue encodes part of a VLC alkane synthase complex (Bernard et al., 2012), as well as genes for a glycosylphosphatidylinositol (GPI)-anchored lipid transfer protein, *LTPG2* (*HORVU.MOREX.r3.3HG0296370*), linked to cuticular wax export/accumulation in Arabidopsis (Kim *et al*., 2012), additional homologues of *CUS2* (*HORVU.MOREX.r3.5HG0486520*) and CDEF1 (HORVU.MOREX.r3.3HG0286360), and a homologue of CDEF2 (HORVU.MOREX.r3.1HG0077180), which has recently been implicated in tomato cutin remodelling along with CDEF1 (Philippe et al., 2025; bioRxiv 2025.01.09.632122). Upregulated genes in *nud^638^* include homologues of *HOTHEAD* (*HORVU.MOREX.r3.5HG0475790*)*,* a gene associated with cutin synthesis in Arabidopsis and rice (Kurdyukov et al., 2006b; Xu et al., 2017), and the *HOTHEAD* paralog *IPE1* linked to cutin synthesis in maize (*HORVU.MOREX.r3.2HG0186860*; Chen *et al*., 2017), as well as homologues encoding epidermal and integument developmental regulators, such as GLABROUS11 (HDG11; *HORVU.MOREX.r3.1HG0006070*; Khosla et al., 2014) and the MYB transcription factor KANADI4 (*HORVU.MOREX.r3.5HG0513030*; McAbee et al., 2006), in addition to ARA12 (HORVU.MOREX.r3.5HG0489760), a subtilisin-like protease associated with mucilage release in Arabidopsis (Rautengarten et al., 2008), and RABA3 (HORVU.MOREX.r3.7HG0721580), a Rab GTPase required for vesicle trafficking during cell division in Arabidopsis (Chow et al., 2008). Impaired HvWIN1 function causes misexpression of a similar but distinct suite of cuticular genes and other developmental regulators (Fig 6g), including downregulation of a barley *glycerol-3-phosphate acyltransferase6* (*GPAT6*; *HORVU.MOREX.r3.4HG0340350*) related to genes important for cutin accumulation and cell wall properties in tomato (Petit et al., 2016; Fawke et al., 2019) and cutin-dependent nanoridge formation in Arabidopsis (Li-Beisson et al., 2009). We also detected upregulation of *KCS17* (*HORVU.MOREX.r3.5HG0474980*) important for waxes in Arabidopsis seed coats (Kim et al., 2024), two wax synthases (*HORVU.MOREX.r3.2HG0138620* and *HORVU.MOREX.r3.7HG0665520*), a second homologue of *HDG11* (*HORVU.MOREX.r3.6HG0618540*), and *KANADI2* (*HORVU.MOREX.r3.6HG0606010*; McAbee et al., 2006), as well as downregulation of *BEL1* (*HORVU.MOREX.r3.1HG0052320*; Reiser et al., 1995). The *HvWIN1* gene itself was also upregulated, suggesting autoregulation (Table S7).

**Video S1 RFP tagged HvBDG1 protein localises to mobile spherical bodies within the cell.** Cellular localisation of transiently expressed RFP-HvBDG1 (magenta) in *Nicotiana benthamiana*leaves 3 days post-infiltration (DPI). Puncta expressing RFP-HvBDG1 can be seen moving within the cytosol close to the plasma membrane. Scale bar: 10um.

**Video S2 RFP tagged HvBDG1 proteins in mobile spherical bodies may move along the ER.** Cellular localisation of transiently expressed RFP-HvBDG1 (magenta) in leaves of a *Nicotiana benthamiana*line expressing an ER marker (HDEL-GFP, green). Puncta containing RFP-HvBDG1 signal can be seen moving along the ER tubules. Scale bar: 10um.

**Supplementary References**

Adamski NM, Bush MS, Simmonds J, Turner AS, Mugford SG, Jones A, Findlay K, Pedentchouk N, von Wettstein‑Knowles P, Uauy C. (2013) The Inhibitor of wax 1 locus (Iw1) prevents formation of β‑ and OH‑β‑diketones in wheat cuticular waxes and maps to a sub‑cM interval on chromosome arm 2BS. *Plant Journal*, **74**, 989–1002.

Bernard A, Domergue F, Pascal S, Jetter R, Renne C, Faure JD, Haslam RP, Napier JA, Lessire R, Joubès J. (2012) Reconstitution of plant alkane biosynthesis in yeast demonstrates that Arabidopsis ECERIFERUM1 and ECERIFERUM3 are core components of a very‑long‑chain alkane synthesis complex. *Plant Cell*, **24**, 3106–3118.

Campoli C, Eskan M, McAllister T, Liu L, Shoesmith J, Prescott A, Ramsay L, Waugh R, McKim SM. (2024) A GDSL‑motif esterase/lipase affects wax and cutin deposition and controls hull–caryopsis attachment in barley. *Plant and Cell Physiology*, **65**, 999–1013.

Chen GT, et al. (2011) An ATP‑binding cassette subfamily G full transporter is essential for the retention of leaf water in both wild barley and rice. *Proceedings of the National Academy of Sciences of the USA*, **108**, 12354–12359.

Chow CM, Neto H, Foucart C, Moore I. (2008) Rab‑A2 and Rab‑A3 GTPases define a trans‑Golgi endosomal membrane domain in Arabidopsis that contributes substantially to the cell plate. *Plant Cell*, **20**, 101–123.

Dvořák J, et al. (2018) Reassessment of the evolution of wheat chromosomes 4A, 5A and 7B. *Theoretical and Applied Genetics*, **131**, 2451–2462.

Fawke S, Torode TA, Gogleva A, Fich EA, Sørensen I, Yunusov T, et al. (2019) Glycerol‑3‑phosphate acyltransferase 6 controls filamentous pathogen interactions and cell wall properties of the tomato and *Nicotiana benthamiana* leaf epidermis. *New Phytologist*, **223**, 1547–1559.

Harrison PW, et al. (2024) Ensembl 2024. *Nucleic Acids Research*, **52**, D891–D899.

Hong L, Brown J, Segerson NA, Rose JKC, Roeder AHK. (2017) CUTIN SYNTHASE 2 maintains progressively developing cuticular ridges in Arabidopsis sepals. *Molecular Plant*, **10**, 560–574.

International Wheat Genome Sequencing Consortium (IWGSC) (2018) Shifting the limits in wheat research and breeding using a fully annotated reference genome. Science, **361**, eaar7191.

Khosla A, Paper JM, Boehler AP, Bradley AM, Neumann TR, Schrick K. (2014) HD‑ZIP proteins GL2 and HDG11 have redundant functions in Arabidopsis trichomes, and GL2 activates a positive feedback loop via MYB23. *Plant Cell*, **26**, 2184–2200.

Kim H, Lee SB, Kim HJ, Min MK, Hwang I, Suh MC. (2012) Characterization of glycosylphosphatidylinositol‑anchored lipid transfer protein 2 (LTPG2) and overlapping function between LTPG/LTPG1 and LTPG2 in cuticular wax export or accumulation in *Arabidopsis thaliana*. *Plant Cell Physiology*, **53**, 1391–1403.

Kurdyukov S, et al. (2006) Genetic and biochemical evidence for involvement of HOTHEAD in the biosynthesis of long‑chain α,ω‑dicarboxylic fatty acids and formation of extracellular matrix. *Planta*, **224**, 315–329.

Li C, et al. (2018) The β‑ketoacyl‑CoA synthase HvKCS1, encoded by *Cer‑zh*, plays a key role in synthesis of barley leaf wax and germination of barley powdery mildew. *Plant and Cell Physiology*, **59**, 811–827.

Li‑Beisson Y, et al. (2009) Nanoridges that characterize the surface morphology of flowers require the synthesis of cutin polyester. *Proceedings of the National Academy of Sciences of the USA*, **106**, 22008–22013.

Maccaferri M, et al. (2019) Durum wheat genome highlights past domestication signatures and future improvement targets. *Nature Genetics*, **51**, 885–895.

McAbee JM, Hill TA, Skinner DJ, Izhaki A, Hauser BA, Meister RJ, Reddy GV, Meyerowitz EM, Bowman JL, Gasser CS. (2006) ABERRANT TESTA SHAPE encodes a KANADI family member, linking polarity determination to separation and growth of Arabidopsis ovule integuments. *Plant Journal*, **46**, 522–531.

Petit J, et al. (2016) The glycerol‑3‑phosphate acyltransferase GPAT6 from tomato plays a central role in fruit cutin biosynthesis. *Plant Physiology*, **171**, 894–913.

Philippe G, Sørensen I, Guérault A, Cross MJ, Domozych DS, Clausen MH, Rose JKC. (2025) Spatiotemporal variation in cutin polymerization and remodeling mediated by GDSL‑hydrolase enzymes during tomato fruit development. *bioRxiv*.

Rautengarten C, Usadel B, Neumetzler L, Hartmann J, Büssis D, Altmann T. (2008) A subtilisin‑like serine protease essential for mucilage release from Arabidopsis seed coats. *Plant Journal*, **54**, 466–480.

Reiser L, Modrusan Z, Margossian L, Samach A, Ohad N, Haughn GW, Fischer RL. (1995) The BELL1 gene encodes a homeodomain protein involved in pattern formation in the Arabidopsis ovule primordium. *Cell*, **83**, 735–742.

Sagado P, Heredia‑Guerrero H, Heredia A, Domínguez E. (2020) Cutinsomes and CUTIN SYNTHASE1 function sequentially in tomato fruit cutin deposition. *Plant Physiology*, **183**, 1622–1637.

Tang J, Yang X, Xiao C, Li J, Chen Y, Li R, Li S, Lü S, Hu H. (2020) GDSL lipase occluded stomatal pore 1 is required for wax biosynthesis and stomatal cuticular ledge formation. *New Phytologist*, **228**, 1880–1896.

Takahashi K, Shimada T, Kondo M, Tamai A, Mori M, Nishimura M, Hara‑Nishimura I. (2010) Ectopic expression of an esterase, which is a candidate for the unidentified plant cutinase, causes cuticular defects in *Arabidopsis thaliana*. *Plant and Cell Physiology*, **51**, 123–131.

Xu Y, Liu S, Liu Y, Ling S, Chen C, Yao J. (2017) HOTHEAD‑like HTH1 is involved in anther cutin biosynthesis and is required for pollen fertility in rice. *Plant and Cell Physiology*, **58**, 1238–1248.

Yeats TH, et al. (2012) CUTIN SYNTHASE1 catalyses polymerisation of plant cutin at the cell surface. *Proceedings of the National Academy of Sciences of the USA*, **109**, 17133–17137.

Zhou Y, et al. (2020) Triticum population sequencing provides insights into wheat adaptation. *Nature Genetics*, **52**, 1412–1422.
